# Novel domain expansion methods to improve the computational efficiency of the Chemical Master Equation solution for large biological networks

**DOI:** 10.1186/s12859-020-03668-2

**Published:** 2020-11-11

**Authors:** Rahul Kosarwal, Don Kulasiri, Sandhya Samarasinghe

**Affiliations:** 1 Centre for Advanced Computational Solutions (C-fACS), Lincoln University, Lincoln, Christchurch, New Zealand; 2 Complex Systems, Big Data, and Informatics Initiative (CSBII), Lincoln University, Lincoln, Christchurch, New Zealand

**Keywords:** Biochemical reaction networks, chemical master equation, stochastic, intelligent state projection, Bayesian likelihood node projection

## Abstract

**Background:**

Numerical solutions of the chemical master equation (CME) are important for understanding the stochasticity of biochemical systems. However, solving CMEs is a formidable task. This task is complicated due to the nonlinear nature of the reactions and the size of the networks which result in different realizations. Most importantly, the exponential growth of the size of the state-space, with respect to the number of different species in the system makes this a challenging assignment. When the biochemical system has a large number of variables, the CME solution becomes intractable. We introduce the intelligent state projection (*ISP*) method to use in the stochastic analysis of these systems. For any biochemical reaction network, it is important to capture more than one moment: this allows one to describe the system’s dynamic behaviour. *ISP* is based on a state-space search and the data structure standards of artificial intelligence (*AI*). It can be used to explore and update the states of a biochemical system. To support the expansion in *ISP*, we also develop a Bayesian likelihood node projection (*BLNP*) function to predict the likelihood of the states.

**Results:**

To demonstrate the acceptability and effectiveness of our method, we apply the *ISP* method to several biological models discussed in prior literature. The results of our computational experiments reveal that the *ISP* method is effective both in terms of the speed and accuracy of the expansion, and the accuracy of the solution. This method also provides a better understanding of the state-space of the system in terms of blueprint patterns.

**Conclusions:**

The *ISP* is the de-novo method which addresses both accuracy and performance problems for CME solutions. It systematically expands the projection space based on predefined inputs. This ensures accuracy in the approximation and an exact analytical solution for the time of interest. The *ISP* was more effective both in predicting the behavior of the state-space of the system and in performance management, which is a vital step towards modeling large biochemical systems.

## Background

In systems biology, it is crucial to understand the dynamics of large and complicated biochemical reaction networks. Recent advances in computing and mathematical techniques mean it is easier for biologists to deal with enormous amounts of experimental data, right down to the level of a single molecule of a species. Such information reveals the presence of a high level of stochasticity in the networks of biochemical reactions. In biochemical reaction networks, stochastic models have made significant contributions to the fields of systems biology [1, 2], neuroscience [3], and drug modeling [4].

In a complex system, biochemical reactions are often modeled as reaction rate equations (RREs) using ordinary differential equations (ODEs). Examples of this kind of work include the biochemical networks of Alzheimer's disease (AD) [5]; the pathways in the fungal pathogen *Candida albicans* [6]; and the COVID-19 coronavirus pathogen network [7]. In each of these examples, the behavior of different pathways is still largely unknown. All these models only contain species with small copy numbers and widely different reaction rates; the probabilistic descriptions of time evolution of molecular concentrations (or numbers) are more suited for understanding the dynamics of such systems. One probabilistic approach for modeling a biochemical reaction network is to deduce a set of integro-differential equations known as chemical master equations (CMEs) [8, 9]. CMEs describe the evolution of the probability distribution over the entire state-space of a biochemical system that jumps from one set of states to another set of states in continuous time: they are a continuous time version of Markov chains (CTMCs) [8, 10] with discrete states. By defining the Markov chain [10, 11], we can consider the joint and marginal probability densities of the species in a system that changes over time [12].

In such cases, the development of *RREs* with molecular numbers becomes very important. The biochemical reaction network can be defined in terms of the discrete state$X\equiv {\left({x}_1,\dots, {x}_{\widetilde{\mathrm{N}}}\right)}^T$ vector of non-negative integers ${x}_{\widetilde{\mathrm{N}}}$ for the given conditions, where $\widetilde{\mathrm{N}}$ ≥1. {*X*(*t*) : *t* ∈ *K*; φ} defines a stochastic process, where *K* is the indexing scheme and φ is the sample space. Following the derivation in [9], for every reaction, there exists a reaction channel, *R*_*M*_, which determines the unique reaction in the system with a propensity function *k*_*M*_. The specific combinations of the reactant species in *R*_*M*_ will react during an infinitesimal [*t*, *t* + *dt*) time interval. The average probability *a*_*μ*_(*X*(*t*))*dt* of a particular *R*_*M*_ fires within [*t*, *t* + *dt*) is the multiplication of the numbers of reactant species, denoted by square brackets, by *k*_*M*_. For example,

$$ {\displaystyle \begin{array}{ll}{\boldsymbol{R}}_{\mathbf{1}}:C\ \overset{k_1}{\to }\ A& {\boldsymbol{a}}_{\mathbf{1}}:{k}_1\left(\left[C\right]\right)\\ {}{\boldsymbol{R}}_{\mathbf{2}}:A+E\overset{k_2}{\to }\ T,& {\boldsymbol{a}}_{\mathbf{2}}:{k}_2\left(\left[A\right].\left[E\right]\right)\\ {}{\boldsymbol{R}}_{\mathbf{3}}:T\ \overset{k_3}{\to }\ C,& {\boldsymbol{a}}_{\mathbf{3}}:{k}_3\left(\left[T\right]\right)\end{array}} $$

In the case where the reactants are of the same type, for example $A+A\overset{k_2}{\to }\ T$, then ${a}_2:{k}_2\left(\frac{\left[A\right]\left[\left(A-1\right)\right]}{2}\right)$. The set consisting of all the reaction channels, *R*_*M*_, is the union of sets of *fast* reactions and *slow* reactions [12]. They are categorized into sets of *R*_*M*(*fs*)_ and *R*_*M*(*sr*)_ reactions, respectively, based on their propensity values. Therefore,

1 $$ {R}_M={R}_{M(fs)}\bigcup {R}_{M(sr)}. $$

A reaction is faster than others if its propensity is of several orders of magnitude larger than the other propensity values (see the list of abbreviations and notations at the end).

### Chemical master equation

In this paper, we consider a network of biochemical reactions at a constant volume. The network consists of $\widetilde{\mathrm{N}}$ ≥1 different species $\left\{{S}_1,\dots {S}_{\widetilde{\mathrm{N}}}\right\}$. They are spatially homogeneous and interact through *M* ≥1 reaction channels in thermal equilibrium. The number of counts of each different species defines the state of the system. If all the species are bounded by *S*, then the approximate number of states in the system would be ${S}^{\widetilde{\mathrm{N}}}$ [13]. Each state $X\equiv {\left({x}_1,\dots {x}_{\widetilde{\mathrm{N}}}\right)}^T$. ${x}_{\widetilde{\mathrm{N}}}$, denotes the number of molecules (counts) of each species. For every state, *X*, the probability satisfies the following CME [8],

2 $$ \frac{\partial {P}^{(t)}(X)}{\partial t}=\sum \limits_{\upmu =1}^M{a}_{\upmu}\left(X-{v}_{\upmu}\right){P}^{(t)}\left(X-{v}_{\upmu}\right)-\sum \limits_{\upmu =1}^M{a}_{\upmu}(X){P}^{(t)}(X) $$

where *P*^(*t*)^(*X*) = the probability function, representing the time-evolution of the system, given that *t* ≥ *t*_0_ and the initial probability is, ${P}^{\left({t}_0\right)}\left({X}_0\right)$,

*M* = elementary chemical reaction channels *R*_1_, … .. *R*_*M*_,

*a*_*μ*_ = chemical reaction propensity of channel *μ* = {1, 2, …. *M*}, and

*v*_*μ*_ = the stoichiometric vector that represents a change in the molecular population of the chemical species due to the occurrence of one *R*_*M*_ reaction. The system transitions to a new state: *X* + *v*_*μ*_ records the changes in the number of counts of different species when the reactions occur.

We note that *a*_*μ*_(*X* − *v*_*μ*_)*dt* is the probability for state (*X* − *v*_*μ*_) to transition to state *X* through chemical reaction, *R*_*M*_, during [*t*, *t* + *dt*), and ${\sum}_{\mu =1}^M{a}_{\mu }(X) dt$ is the probability for the system to shift from state *X* as a result of any reaction during *dt*. If ${\mathbf{X}}_J=\left\{{X}_1,\dots \dots .{X}_{S^{\widetilde{\mathrm{N}}}}\right\}$ is the ordered set of possible states of the system indexed by {1, 2…*K*} having ${S}^{\widetilde{\mathrm{N}}}$ elements, then Eq. (2) represents the set of ordinary differential equations (ODEs) that determines the changes in probability density *P*^(*t*)^= (*P*^(*t*)^(*X*_1_), … ${P}^{(t)}\left({X}_{S^{\widetilde{\mathrm{N}}}}\right)\Big){}^T$. Once **X**_*J*_ is selected, the matrix-vector form of Eq. (2) is described by an ODE:

3 $$ \frac{\partial {P}^{(t)}}{\partial t}=A.{P}^{(t)}, $$

where the transition rate matrix is *A* = [*a*_*i*,*j*_]. If each reaction leads to a different state, ${X}_{i^{\prime }}$, then the elements in submatrix *A*_*i*,*j*_ are given as:

4 $$ {A}_{i,j}=\left\{\begin{array}{c}-\sum \limits_{\mu =1}^M{a}_{\mu}\left({X}_i\right),\mathrm{if}\ i=j\\ {}{a}_{\mu}\left({X}_i\right),\mathrm{if}\ {X}_{i^{\prime }}={X}_i+{v}_{\mu}\\ {}0,\mathrm{otherwise}\end{array}\right. $$

This equation represents the infinitesimal generator of the Markov process [10, 14, 15]. Rows and columns are ordered in lowercase letters, *i* and *j* respectively. The entry of *a*_*i*,*j*_ of the matrix determines the propensity for the chemical system to transition from one state to another state, given that *i* ≠ *j*, are non-negative. The diagonal terms of the matrix are defined by *a*_*jj*_, when *i* = *j* and the matrix has a zero-column sum, so its probability is conserved. From Eq. (3) we can derive the ${P}^{\left({t}_f\right)}$ probability vector at the final time, *t*_*f*_, of interest given an initial density of ${P}^{\left({t}_0\right)}$:

5 $$ {P}^{\left({t}_f\right)}=\mathit{\exp}\left({t}_fA\right).{P}^{\left({t}_0\right)}, $$

where the matrix exponential function is defined by the convergent Taylor series as [16, 17]

6 $$ \mathit{\exp}\left({t}_fA\right)=I+\sum \limits_{n=1}^{\infty}\frac{{\left({t}_fA\right)}^n}{n!}. $$

However, algorithms, such as in [13, 18–20] truncate Eq. (6) infinite summation to approximate Eq. (3) at the cost of a truncation error.

### Initial value problem

If *v*_*μ*_ or *v*_*M*_, for *μ* or *M* = {1, 2, …. *M*} are the stoichiometric vectors for *R*_*M*_ reaction channels, then we will define the stoichiometric matrix for the system by *V*_μ_ or *V*_*M*_ = [*v*_1_; *v*_2_; ……*v*_μ_]^*T*^. If φ is the sample space and *X*_0_ ∈ φ is the initial state of the system, **X**_*J*_ denotes the only set of states in φ. To solve *P*^(*t*)^(*X*) in Eq. (2) for *X* ∈ φ, we define the *P*^(*t*)^ vector as(*P*^(*t*)^(*X*))_*X* ∈ φ_ or ${\left({P}^{(t)}(X)\right)}_{X\in {\mathbf{X}}_J}$ for a finite set of states, then $\frac{\partial {P}^{(t)}}{\partial t}$ is defined as a vector ${\left(\frac{\partial {P}^{(t)}}{\partial t}\right)}_{X\in \upvarphi}$. Solving the CME involves finding the solution of the initial value problem over a time period using the differential equation Eq. (3) when *t* > 0, whereas, ${P}^{\left({t}_0\right)}$ is the initial distribution at *t* = 0. Here, the sample space φ can be infinite for large biochemical systems. Finding the solution for Eq. (3) for the given parameters with a finite set of states **X**_*J*_ is a major problem for CME’s because in large biochemical systems the size of *A* will be extremely large.

For example, consider an enzymatic reaction network [13] described by reactions ${R}_1:S+E\overset{k_1}{\to }\ C$, ${R}_2:C\overset{k_2}{\to }\ S+E$, ${R}_3:C\ \overset{k_3}{\to }\ P+E$. This network of reactions involves four species: namely, *S*− substrate, *E*− enzyme, *C*− complex and *P*− product molecules. The *X* ≡ (*x*_1_, *x*_2_, *x*_3_, *x*_4_)^*T*^ ≡ (*S*, *E*, *C*, *P*)^*T*^ represents any state of the system, with *X*_0_ ≡ (*S*_0_, *E*_0_, *C*_0_, *P*_0_) given as the initial state. The stoichiometric vectors are given by *v*_1_ = (−1, −1, 1, 0), *v*_2_ = (1, 1, −1, 0), *v*_3_ = (0, 1, −1, 1). Therefore, for (*x*_1_, *x*_2_, *x*_3_, *x*_4_) ${x}_{\widetilde{\mathrm{N}}=4}$, the propensity functions are:

$$ {\displaystyle \begin{array}{l}{R}_1:{a}_1\left(\left[{x}_1\right],\left[{x}_2\right],\left[{x}_3\right],\left[{x}_4\right]\right)={k}_1\times {x}_1(t)\times {x}_2(t)\\ {}{R}_2:{a}_1\left(\left[{x}_1\right],\left[{x}_2\right],\left[{x}_3\right],\left[{x}_4\right]\right)={k}_2\times {x}_3(t)\\ {}{R}_3:{a}_1\left(\left[{x}_1\right],\left[{x}_2\right],\left[{x}_3\right],\left[{x}_4\right]\right)={k}_3\times {x}_3(t)\end{array}} $$

The set of states reachable from *X*_0_ is finite in number. With multiple explosions of the number of states in a large model, the size of *A* increases exponentially.

As seen in Eq. (5), solving Eq. (2) becomes a problem when the model’s dimensions grow due to the increase of species present in the system. This is particularly true for large biochemical models. The approximate estimate of ${S}^{\widetilde{N}}$ shows how the size of the problem increases. This explosion in size is known as the *curse of dimensionality* [9, 13]. The CME solution given in Eq. (5) has two major parts: (a) the expansion of the state-space, and (b) the approximation of the series. For the expansion of state-space, Finite State Projection (*FSP*) [21] and Sliding Windows (*SW*) [18] are used to find the domain. Methods like Krylov subspace [13] and Runge Kutta [22] are commonly used for approximation (of the series) of the CME Eq. (5).

Although CME has been employed and solved explicitly for relatively small biological systems [13, 18–20, 23, 24], computationally complaisant but accurate solutions are still unknown for most significant systems and for large systems which have an infinite (or a very large) number of states. This lack of closed-form solutions has driven the system biology research towards *Monte-carlo Algorithms* (*MC*) [25] to capture dynamics. One algorithm, the *Stochastic Simulation Algorithm* (*SSA*) by Gillespie [9], has been used in the CME. Although the original *FSP* state-space expansion has been used in research [21, 26], it has some drawbacks [21]. The *FSP* [21] and its variants [20, 24, 26, 27] are based on *r-step reachability* [26]. While *SW* [18] is also a *FSP* based method, it employs a stochastic simulation algorithm (*SSA*) to find the domain. This is more effective than *FSP* and suitable for stiff problems. Add-on weighting functions like *GORDE* [28] and likelihoods [24] methods are used to improve the expansion. *FSP GORDE* [28] removes the states with small probabilities before the calculation of Eq. (5). This practice saves computational time, meaning that *FSP GORDE* performs faster than conventional *FSP r-step reachability.* However, removing the probabilities before the calculation of Eq. (5) increases the steps error and affects the accuracy of the final solution at *t*_*f*_ regardless of whether the state-space is small or large. If one is interested in solving stiff and/or large systems, it will greatly affect the solution.

The *FSP* variant, *Optimal Finite State Projection* (*OFSP*), [20] based on *r-step reachability*, performs better in terms of producing optimal order domain. It is faster than both *FSP* and *FSP GORDE*. However, it is infeasible to use *SW* for large CME problems because creating hyper-rectangles is a very difficult task. At least four-times the number of *SSA* simulations are required to minimize the error by half, because of very low convergence rates of routines in *MC* . The original *SSA* takes a long time, because one simulation may have several different *R*_*M*_. Recently, the *SSA*’s efficiency has been greatly enhanced by researchers through various schemes such as *τ* leaps (adaptive) [29, 30]. Thus, we compare the *OFSP* and *SSA* (*τ* leaps adaptive) against the *ISP* in terms of finding the domain, accuracy and computational efficiency. Key to solving the CME remains in finding the right projection size (domain) for large models which would then ensure efficient approximation.

In this paper, we focus primarily on developing the expansion strategy, namely the *Intelligent State Projection* (*ISP*) method, to mitigate several problems: the accuracy of the solution, the performance of the method and projection size. The *ISP* has two variants: the *Latitudinal Search* (*LAS*) and the *Longitudinal-Latitudinal Search* (*LOLAS*). It treats the Markov chain of a biochemical system as a Markov chain tree structure and states as objects of class *node*. Based on the dimension of the system, search is performed in a latitudinal way for different model sizes using the *ISP LAS* method. Whereas, bidirectional search is applied using *ISP LOLAS*, which quickly expands the state-space up to a specified bound limit. To support the expansion strategy, we also develop the *Bayesian Likelihood Node Projection* (*BLNP*) function, based on Bayes’ theorem [31, 32]. It is adjoined with the *ISP* variants to determine the likelihood of events at any interval at the molecular population level. *BLNP* provides confidence to the expansion strategy by assigning probability values to the occurrence of future reactions and prioritizing the direction of expansion. The *ISP* embedding *BLNP* function inductively expands the multiple states with the likelihood of occurrence of fast and slow reactions [12]. It also defines the complexity of the system by predicting the pattern of state-space updation, and the depth of the end state from the initial state. When used for any size of biological networks, *LAS’* memory usage is proportional to the entire width of expansion; it is less than *ISP LOLAS*. Both methods are feasible and differentiated for various types of biological networks. However, the computational time for both variants depend on the nature of the model and the size of the time step used. At any point, the amount of memory in use is directly proportional to the neighboring states reachable through a single *R*_*M*_ reaction. *ISP LOLAS* uses considerably less memory, even when it retracts to the initial node to track new reactions, then revisiting the depth many times.

## Results

Having discussed the CME solution, we now discuss the modeling and integration of the biochemical reaction systems for the *ISP* methods, as well as the assumptions underlying these methods. Using *ISP*, we tested its ability to reproduce the model to measure dynamics of the key parameters in the models. The *ISP* method is a novel, easy-to-use, technique for modeling and expanding the state-space of biochemical systems. It features several improvements in modeling and computational efficiency.

The computational experiment (initializing and solving the model) was conducted on the carbon-neutral platform of Amazon® Web Service Elastic Computing (EC2), instance type large (m5a), running on HVM (hardware virtual environment) virtualization with variable ECUs. We used multicore environment 16vCPU @ 2.2GHz, AMD EPYC 7571 running Ubuntu 16.04.1 with relevant dependencies, and 64GB memory with 8GB Elastic Block Storage (EBS) type General Purpose SSD (GP2) formatted with Elastic File System (EFS). The performance mode was set to General Purpose with input-output per second (IOPS = 100/3000). We used the type bursting throughput mode (see Supplementary Information (SI) 1).

### Intelligent state projection

The main aim of the proposed algorithm is to expand the **X**_*K*_ iteratively, such that **X**_*K*_ contains a minimum number of states carrying the maximum probability mass of the system. To create the sample space for *ISP*, a Markov chain tree Ѭ [33] was used to visualize a biochemical system to exhibit the transition matrix as directed trees [10, 11] of its associated graph. Additionally, the Markov chain tree Ѭ generates sample space of the system to represent Markov processes associated with the Markov chain and the transition matrices of biochemical reaction networks. In following section, we visualize the Markov chain of the biochemical system as a Markov chain graph (tree) for *ISP* compatibility.

#### Markov chain as a Markov chain tree

We define the Markov chain tree, Ѭ, [33] as infinite and locally finite. It is a special type of graph with a prominent vertex called a parent node without loops or cycles. If graph *G*_*mc*_ is a state-space of the finite state Markov chain with $\left\{P\left({X}_i,{X}_{i^{\prime }}\right)\ \right|\ {X}_i,{X}_{i^{\prime }}\in {G}_{mc}\Big\}$ transition probabilities meeting the condition $\sum \limits_{X_{i^{\prime }}}P\left({X}_i,{X}_{i^{\prime }}\right)=1$, then the induced Markov chain tree is a combination of valued *G*_*mc*_ random variables with the distributions inductively defined from $P\left({X}_i,{X}_{i^{\prime }}\right)$ with an initial state, *X*_*i*_ ∈ *G*_*mc*_. That being the case, it is easy to expand this class of Markov random field through a Markov chain tree structure for biochemical systems. Furthermore the Markov chain tree and the Markov processes can be equated as explained in [34] for the stochastic analysis.

Since we are interested in aperiodic states in the expansion of state-space, we shall assume the reducibility or simplification of the *G*_*mc*_; namely for each ${X}_i,{X}_{i^{\prime }}\in {G}_{mc}$ through Ѭ. Therefore, let us concentrate on the case where *G*_*mc*_ is considered as a locally finite connected graph. The transition probabilities of each state are not equal due to the propensities and parameters of different reactions in the biochemical system. Consequently, a Markov chain tree, Ѭ, can be used to visualize a biochemical system process to exhibit a transition matrix as directed trees of its associated graph [10, 11]. It can also be used to generate a sample space for the system to represent the Markov processes and the transition matrices of biochemical reaction networks. We discuss the details needed to represent Markov models on trees and working with graphs for state-space later.

**Fig. 1:**
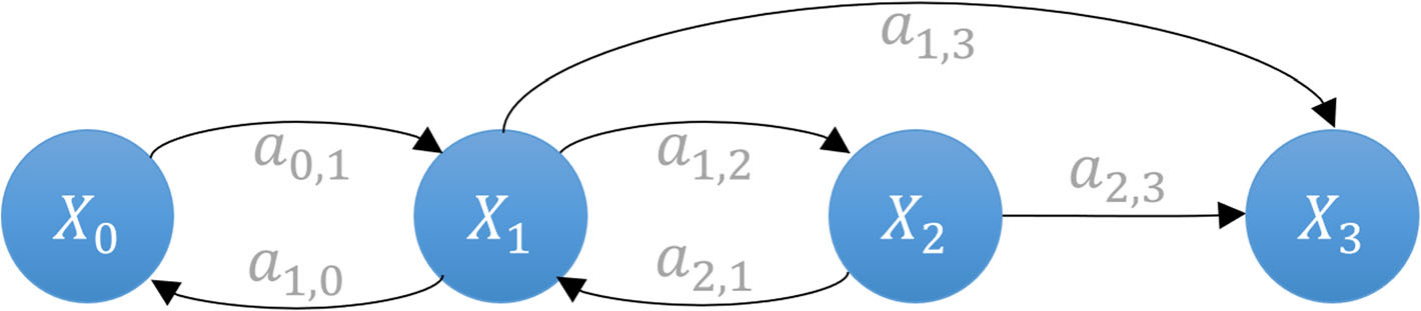
Markov chain graph showing forward and reversible reactions through four different states

If **X**_*J*_ is the finite set of cardinality {1, 2……. *K*} of a Markov chain Ѫ_*c*_, then *A* is the transition probability matrix associated with **X**_*J*_. A state-space is, substantially, a class of a set of states containing the unique state of the system. The arcs between the states represent the transitions from the initial state to the end state. This transition is defined as transient and communicating class in graphs. When all the transitions are combined, every state-space takes the form of a graph and creates the state-space of the system, as shown in Fig. 1 below.

We can now associate chain Ѫ_*c*_ with the directed graph *G*_*mc*_ = (**X**_*J*_, *V*_*μ*_), where *V*_*μ*_ = [*v*_1_; *v*_2_; ……*v*_μ_]. *v*_μ_ defines the transition from state *X*_*i*_ to ${X}_{i^{\prime }}$ and is denoted as ${v}_{\upmu}=\left\{\left({X}_i,{X}_{i^{\prime }}\right);{a}_{i,j}>0\right\}$. For every transition $\left({X}_i,{X}_{i^{\prime }}\right)\in {\mathbf{X}}_J$, then weight $\omega \left({X}_i,{X}_{i^{\prime }}\right)$ is *a*_*i*, *j*_.

Suppose *G*_*mc*_ has a cycle, which starts and terminates at some state, *X*_*i*_ ∈ **X**_*J*_. If there is a transition from *X*_*i*_ to ${X}_{i^{\prime }}$, we add a unique transition by creating a cycle from *X*_*i*_ back to itself and then consider the original transition from *X*_*i*_ to ${X}_{i^{\prime }}$. This contradicts the uniqueness of the walk in tree [35]. In terms of the CTMC of a biochemical system process, the change in molecular population is defined by a stoichiometric vector, so, in *G*_*mc*_, there must be at least one intermediate state that will send the system back to the previous state to create the cycle. This process categorizes the *forward* and *backward* reactions given the initial state, *X*_0_, of the system. The transient class of the transition leads the system to a unique state that defines the *forward* reaction in the system. In contrast, the communicating class of a transition defines the reversible reaction in the system. We define such systems as transient class systems and communicating class systems. Large biochemical systems are usually a combination of both classes.

A biochemical system is visualized as a tree Ѭ [33] to enable the expansion of the state-space. A tree, Ѭ, is a special form of graph in data structure constituting a set of nodes and a collection of edges (or arcs), each of which connects to an ordered pair of nodes. *G*_*mc*_ is considered a directed tree, Ѭ. It is rooted with *N*_0_ = (*X*_0_, ƌ_*l*_) if it contains a unique walk to *N*_*i*_ = (*X*_*i*_, ƌ_*l*_ + 1) and does not contain any cycles. Meanwhile, if *X*_*i*_ ∈ **X**_*J*_\{*X*_0_} has exactly one outgoing transition away from *X*_0_ it is called an arborescence. If it makes its transition towards *N*_0_ = (*X*_0_, ƌ_*l*_) it is called an anti-arborescence. An arborescence is a subset ⊆*V*_*μ*_ that has one edge out of every node that contains no cycles and has maximum cardinality. For example, if set *U* = {5, 7, 8, 10} contains 4 elements, then the cardinality of ∣*U*∣ is 4.

**Fig. 2:**
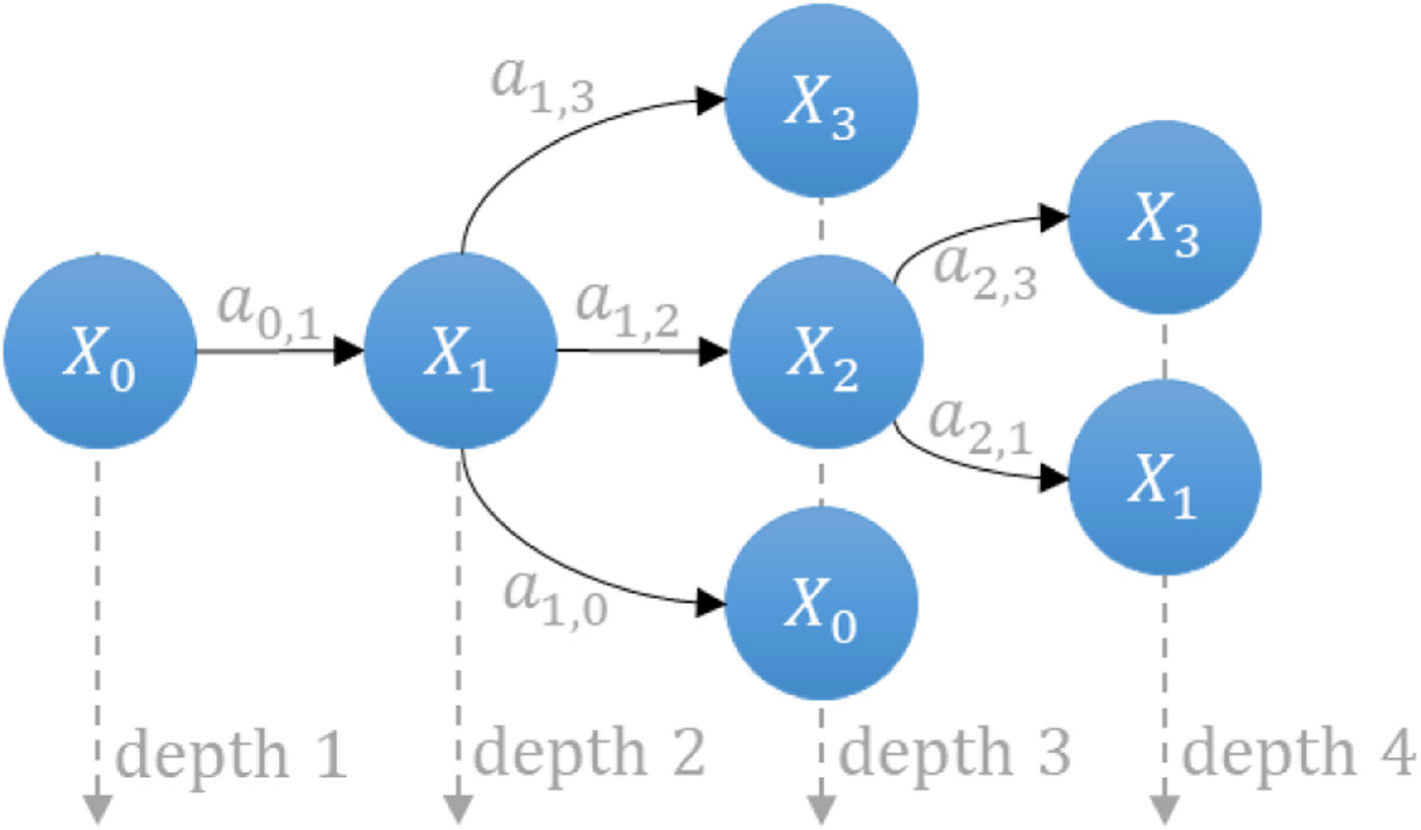
Equivalent tree of a Markov chain graph, as shown in Fig. 1. This is a special form of graph which has no cycle and no self-loops. It depicts the state-space of the system in the form of a tree (*DAG*)

If *X*_*i*_ and ${X}_{i^{\prime }}$are the states other than the initial *X*_0_ state, there is a transition from *X*_*i*_ to ${X}_{i^{\prime }}$, so *X*_*i*_ has at least one transition. Now, suppose *X*_*i*_ has two walks, (*X*_*i*_, *X*_*i* + 1_) and (*X*_*i*_, *X*_*i* + 2_). Concatenating these walks to the walks (*X*_*i* + 1_, ${X}_{i^{\prime }}$) and (*X*_*i* + 2_, ${X}_{i^{\prime }}$), respectively, we have two distinct changes in state from *X*_*i*_ to ${X}_{i^{\prime }}$ in *G*_*mc*_. However, in Ѭ, this concatenation is not considered, which makes them Directed Acyclic Graphs (*DAG*) (see Fig. 2). Most of the biochemical models *G*_*mc*_ can be visualized as *DAG*s irrespective of the nature of the reactions present in the model. Figure 2 shows the equivalent *G*_*mc*_tree of shown in Fig. 1. The trees are less complex as they have no cycles, no self-loops. Yet they are still connected, meaning they can depict the state-space.

The weight of the tree containing all *e* edges is defined by , where $\omega (e)=\omega \left({X}_i,{X}_{i^{\prime }}\right)={a}_{i,j}$ is the weight of an edge starting from *X*_*i*_ and ending at ${X}_{i^{\prime }}$ when  [36]. For systems which have both *forward* and *backward* reactions, if **n**_*J*_ is the total number of nodes indexed by {1, 2…*K*} the same as states, then **n**_*K*_ is the set of nodes carrying **X**_*K*_, and ${\mathbf{n}}_K^{\prime }$ is the set of nodes carrying ${\mathbf{X}}_K^{\prime }$ given *N*_0_ root node of the tree Ѭ, then the walk from one node to another node is given by:

7 $$ \left\{f\left({N}_i,{N}_{i^{\prime }}\right),f\left({N}_{i^{\prime }},{N}_i\right)\ |\ {N}_0\right\}\in {\mathbf{X}}_J, $$

Ѭ is formed by superimposing the forward transitions between the states *X*_*i*_ and ${X}_{i^{\prime }}$, with the reverse orientation. Where ${X}_{i^{\prime }}$ and *X*_*i*_, indicate backward reactions, these are graphically denoted as an individual edge from *N*_*i*_ = (*X*_*i*_, ƌ_*l*_) to *N*_*i*'_ =  (*X*_*i*_, ƌ_*l*_ + 11) to *N*_*i*_ = (*X*_*i*_, ƌ_*l*_ + 2) in a tree. The *N*_*i*_ of ƌ_*l*_ + 2 can be renamed as a new node. *N*_*i* + 1_, remains as it is at a different depth from the *N*_*i*_ of ƌ_*l*_ but contains the same state *X*_*i*_. In the expansion, repeated states are not considered in the domain; therefore, any node which carries a similar state is considered the same, regardless of the level and indexing. Consideration of trees for the state-space expansion in *ISP* not only helps to reduce the complexity but also improves the accuracy of the solution of Eq. (5) by identifying nodes which carry probable states. If the Markov chain graph starts in state *X*_*i*_ ∈ **X**_*J*_, then the mean number of transits to any state ${X}_{i^{\prime }}$ converging to $\overline{a_{X_i,{X}_{i^{\prime }}}}$ is given by the (*i*, *i*^′^)^th^ value of

8 $$ \overline{\mathrm{A}}=\underset{n\to \infty }{\mathit{\lim}}\left(\frac{1}{n}\right)\sum \limits_{k=0}^{n-1}{A}^k. $$

*U* is the set of all arborescences. Let ${U}_{X_i,{X}_{i^{\prime }}}$ is the set of all arborescences which have a transition from *X*_*i*_ to ${X}_{i^{\prime }}$ and $\left\Vert {U}_{X_i,{X}_{i^{\prime }}}\right\Vert$ is the sum of the weights of the arborescences in ${U}_{X_i,{X}_{i^{\prime }}}$ then according to the Markov chain tree theorem [33],

9 $$ \overline{a_{X_i,{X}_{i^{\prime }}}}=\frac{\left\Vert {U}_{X_i,{X}_{i^{\prime }}}\right\Vert }{\left\Vert U\right\Vert } $$

$\overline{a_{X_i,{X}_{i^{\prime }}}}$ is probabilistic in nature. This nature is not only restricted to the systems which have irreducible Markov chains, in which graph *G*_*mc*_ is strongly connected while carrying probable state-spaces, but also for the systems that can be simplified by converting to a Markov chain tree and then by reducing that tree by ignoring the states which have low probabilities in space φ.

#### Expansion criterion for state space

As previously mentioned, the states are indexed using {1, 2……. *K*} in the domain denoted by set **X**_*J*_. To derive the time, based on the state-space expansion conditions, the probability exponential form of the CME Eq. (5) is evaluated for approximation up to the desired final time *t*_*f*_ in steps. To focus on the probable states that contribute most to the probability mass in the domain, we first define the set of non-probable states (those which have the least probability mass) as ${\mathbf{X}}_K^{\prime }$, which are to be bunked. The number of states will usually be infinite, without selecting probable states for the domain. By doing this we can avoid recalculating the probabilities and decrease the computational efforts by keeping the domain small. This bunking can also be applied to the initial distribution of the system at *t*_0_. If submatrix ${A}_j^{\prime }$ contains the non-probable set ${\mathbf{X}}_K^{\prime }$ of states, then the probability of set will be,

10 $$ {P}^{(t)}\left({\mathbf{X}}_K^{\prime}\right)=\mathit{\exp}\left(t.{A}_j^{\prime}\right).{P}^{(t)}\left({X}_0\right). $$

The criterion for defining the non-probable states is determined by the *τ*_*m*_ tolerance value. ${A}_j^{\prime }$ will only be considered to have non-probable states if,

11 $$ {A}_j^{\prime }=\left\{\begin{array}{l}\mathrm{nonprobable}\kern0.5em \mathrm{states},\\ {}\mathrm{else},\\ {}\mathrm{probable}\kern0.5em \mathrm{states},\end{array}\right.\kern0.5em {\displaystyle \begin{array}{c}\mathrm{if}\kern0.5em {P}^{(t)}\left({\mathbf{X}}_K^{\prime}\right)<{\tau}_m\\ {}\mathrm{if}\kern0.5em {P}^{(t)}\left({\mathbf{X}}_K^{\prime}\right)\ge {\tau}_m\end{array}} $$

Similarly, submatrix *A*_*j*_ has a probable set **X**_*K*_ of states if *P*^(*t*)^(**X**_*K*_) ≥ *τ*_*m*_ otherwise, the states from **X**_*K*_ are bunked to ${\mathbf{X}}_K^{\prime }$ if *P*^(*t*)^(**X**_*K*_) < *τ*_*m*_. For any iteration, if ${P}^{(t)}\left({\mathbf{X}}_K^{\prime}\right)\ge {\tau}_m$ then (from Eq. (11)) some states from ${\mathbf{X}}_K^{\prime }$ return to **X**_*K*_ in the next iteration to increase the accuracy of the approximate solution (Ӕ). The column sum of the approximate solution (Ӕ) of these states is defined as:

12 $$ \mathrm{\hbox{\AE}}={I}^T\mathit{\exp}\left({t}_f{A}_j\right).{P}^{(t)}\left({X}_0\right), $$

where, *I* = (1, …1)^*T*^ is of an appropriate length. Declaring some states as non-probable and removing them before calculating the probabilities as seen in [28] will decrease the accuracy of Ӕ with the cumulative step errors. This can be validated from the state probabilities that have been ignored in the domain:

13 $$ \mathrm{\hbox{\AE}}=1-{P}^{(t)}\left({\mathbf{X}}_K^{\prime}\right). $$

We define the step error in terms of the probabilities bunked. If ${e}_{rror}\propto {P}^{(t)}\left({\mathbf{X}}_K^{\prime}\right)$ then,

14 $$ {e}_{rror}=1-{I}^T\mathit{\exp}\left({t}_f{A}_j\right).{P}^{(t)}\left({X}_0\right) $$

15 $$ {e}_{rror}=1-\mathrm{\hbox{\AE}} $$

Every expansion step explores at least one new state and change {**X**_*K*_} but not necessarily $\left\{{\mathbf{X}}_K^{\prime}\right\}$ if:

16 $$ {P}^{(t)}\left({\mathbf{X}}_K\right)\ge {\tau}_m>{P}^{(t)}\left({\mathbf{X}}_K^{\prime}\right), $$

is satisfied. For ideal systems with a given initial probability of ${P}^{\left({t}_0\right)}\left({X}_0\right)$, the $\left\{{\mathbf{X}}_K^{\prime}\right\}$ should be *null* and so ${P}^{\left({t}_f\right)}\left({\mathbf{X}}_K^{\prime}\right)=0$. For such systems $\left\{{\mathbf{X}}_K\right\},\left\{{\mathbf{X}}_K^{\prime}\right\}\ \epsilon\ \left\{{\mathbf{X}}_J\right\}$ for final projection and,

17 $$ {P}^{\left({t}_f\right)}\left({\mathbf{X}}_J\right)={P}^{\left({t}_f\right)}\left({\mathbf{X}}_K\right)+{P}^{\left({t}_f\right)}\left({\mathbf{X}}_K^{\prime}\right), $$

18 $$ {P}^{\left({t}_f\right)}\left({\mathbf{X}}_J\right)={P}^{\left({t}_f\right)}\left({\mathbf{X}}_K\right)+0. $$

${P}^{\left({t}_f\right)}\left({\mathbf{X}}_J\right)$ in Eq. (18) is the solution of Eq. (3) after the state-space is expanded to **X**_*K*_. However, for large biochemical systems, Eq. (18) may not hold completely true, due to the nature ((*fast* (*R*_*M*(*fs*)_) and *slow* (*R*_*M*(*sr*)_)) of some reactions present in the system; therefore, the condition in Eq. (11) will pass the states from ${\mathbf{X}}_K^{\prime }$ to **X**_*K*_. The states with the lowest probabilities will be bunked when:

19 $$ {P}^{(t)}\left({\mathbf{X}}_K^{\prime}\right)<<{P}^{\left({t}_f\right)}\left({\mathbf{X}}_K\right), $$

This improves the solution. Removing without calculating the probabilities of some states is one of the lags of the current methods [18, 20, 21, 24, 26–28]; it is a result of achieving the truncated domain and saving computation time. To address this, we set a ${P}^{(t)}\left({\mathbf{X}}_K^{\prime}\right)$ leakage point based on:

20 $$ {P}^{(t)}\left({\mathbf{X}}_K\right)\ge {\tau}_m(leak)>{P}^{(t)}\left({\mathbf{X}}_K^{\prime}\right), $$

where, *τ*_*m*_(*leak*) for systems will reform Eq. (16) as:

21 $$ {P}^{(t)}\left({\mathbf{X}}_K\right)\ge {\tau}_m\ast 0.4>{P}^{(t)}\left({\mathbf{X}}_K^{\prime}\right), $$

which would then zip the ${\mathbf{X}}_K^{\prime }$ further by leaking the highest probabilities to **X**_*K*_ so that the probability sum is no longer conserved. The motivation of setting this ration is to reconsider (up to 40% of ${\mathbf{X}}_K^{\prime }$) the bunked states to improve the Ӕ solution and decrease the expansion step error. While modeling the biochemical system, if the *slow* and *fast* reaction [12] criterion is considered during expansion, then *τ*_*m*_(*leak*) will be defined as,

22 $$ =\left\{\begin{array}{c}\begin{array}{c}{\tau}_m\ast \frac{\left(\mathrm{no}.\mathrm{of}\ {R}_{M(sr)}\right)}{\left(\mathrm{no}.\mathrm{of}\ {R}_{M(fs)}\right)},\kern0.5em \mathrm{if}\ \mathrm{no}.\mathrm{of}\ {R}_{M(sr)}<\mathrm{no}.\mathrm{of}\ {R}_{M(fs)}\\ {}\mathrm{else},\kern24.50em \\ {}{\tau}_m\ast \frac{\left(\mathrm{no}.\mathrm{of}\ {R}_{M(fs)}\right)}{\left(\mathrm{no}.\mathrm{of}\ {R}_{M(sr)}\right)},\kern0.5em \mathrm{if}\ \mathrm{no}.\mathrm{of}\ {R}_{M(sr)}>\mathrm{no}.\mathrm{of}\ {R}_{M(fs)}\ \end{array}\\ {}\mathrm{else},\kern24.50em \\ {}{\tau}_m\ast 0.4,\kern7.75em \ \mathrm{if}\ \mathrm{no}.\mathrm{of}\ {R}_{M(fs)}=\mathrm{no}.\mathrm{of}\ {R}_{M(sr)}.\end{array}\right. $$

We consider Eq. (21) criterion for all the computational experiments in this study. The conditions in Eqs. (21) and (22) will lead to an optimal set of states as,

23 $$ {\mathbf{X}}_K\longleftarrow {\mathbf{X}}_K-{\mathbf{X}}_K^{\prime }, $$

at *t*_*d*_ in the domain. When **X**_*K*_ is updated at every *t*_*step*_ before reaching *t*_*f*_, this creates several intermediate domains which we define as *Bound*. At *t*_0_, the domain only has the initial state of the system; therefore, we define the *Bound* as:
24

**Fig. 3:**
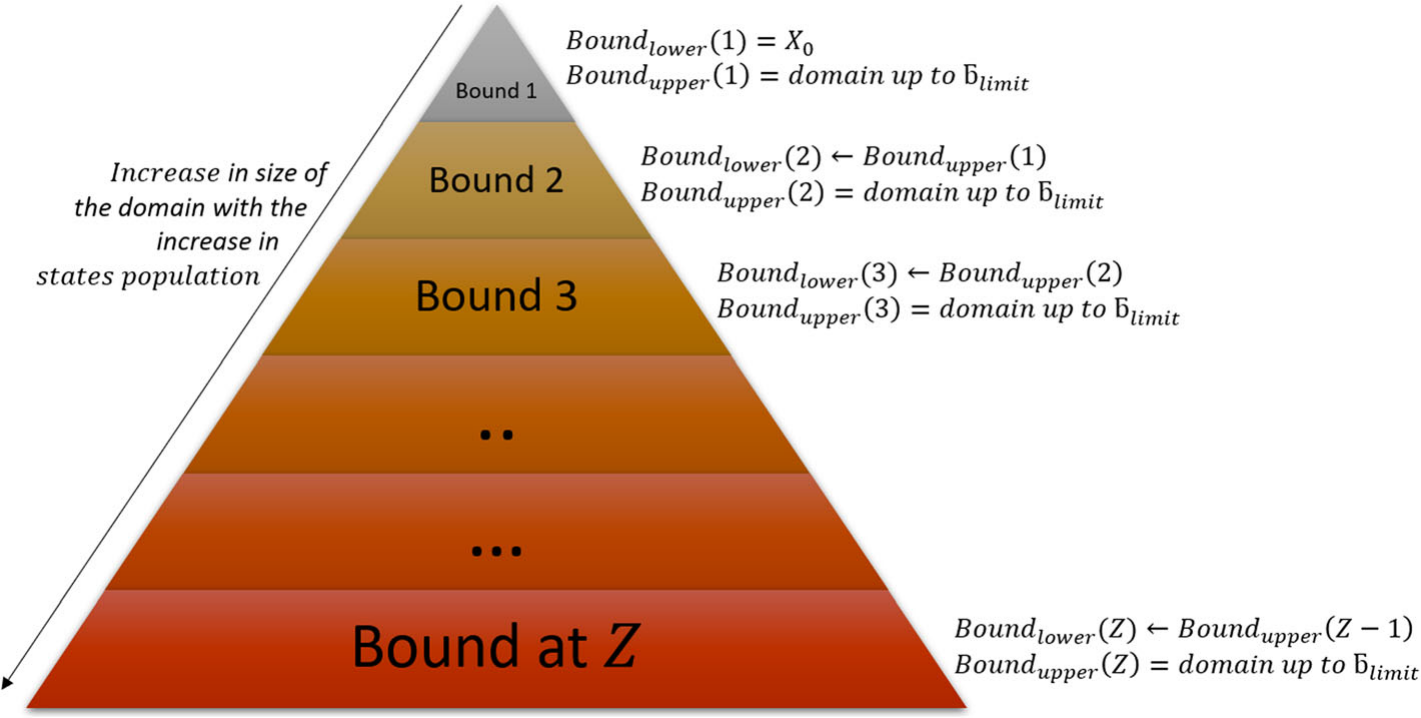
General framework of *2D pyramid* domain showing increases in domain size concomitant with the increase in state with an increase in the bounds. ***Bound***_***lower***_(**1**) represents the initial condition, whereas ***Bound***_***upper***_(***Z***) represents the final domain which carries the explored set of states of the system

After a single *t*_*step*_ of expansion, if **X**_*K*_ is updated with a new state or set of states, it creates:
25

at *t*_*d*_. Here, ƌ_*l*_ denotes the depth level of the latest state or set of states that has been added in the domain to form *Bound*_*upper*_. This *Bound*_*upper*_ is re-labeled and considered as *Bound*_*lower*_ for the next *t*_*step*_ of the expansion. If the expansion is to be limited in the number of *Bounds*, then every *count*(**ƃ**_*limit*_) leads to:
26

where, ƃ_*limit*_ is the bound limit. For example, if ƃ_*limit*_ ***=*** 2, then *the count*(ƃ_*limit*_) will be from $0\overset{\mathrm{to}}{\to }1\overset{\mathrm{to}}{\to }2$. If the *count*(ƃ_*limit*_) is increased to ƃ_*limit*_ for *I*_*tr*_^th^ iterations, then *Bound*_*upper*_ in the current iteration will be *Bound*_*lower*_ for the next iteration. Every *Bound*_*lower*_ state will be the strict subset of every consecutive *Bound*_*upper*_ given as:

27 $$ {Bound}_{lower}(Z)\subset {Bound}_{upper}(Z). $$

and the upper bound as:

28 $$ {Bound}_{upper}(Z)=\left\{ Domain\  at\ {Z}^{th} iteration,{a}_l\right\}, $$

where *Z* is the number of *Bounds* (or intermediate domains). The *2D pyramid* domain in Fig. 3 graphically illustrates the increase in the population of states in the domain with the increase in iterations (*I*_*tr*_). The apex of the pyramid represents the initial state *X*_0_ of the system at *Bound*_*lower*_(1) at *t*_0_, whereas the base represents the deepest level where the system ends with the final domain carrying set **X**_*K*_ with the maximum probability mass.

For large biochemical systems, the number of created *Bounds* are based on *I*_*tr*_. They have million/billions of states. Expansion can be terminated by defining time *t*_*f*_ at which the solution is required. To have an auto break-off point in the expansion, it is first necessary to define the criteria that limits *I*_*tr*_ or when no more new states can be searched. Therefore, we define this criterion in the following section. This criterion also applies to biochemical systems which have *fast* and *slow* reactions [12].

#### Cease of criterion after updating

In every expansion step, the domain is validated by Eq. (21) and new states are added in **X**_*K*_ as long as:

29 $$ 1-{I}^T\mathit{\exp}\left({t}_f{A}_j\right).{P}^{(t)}\left({X}_0\right)\ge {\tau}_m, $$

is satisfied for probable states and stops if Eq. (29) is not satisfied. This leads to a point at *t*_*f*_ where *e*_*rror*_ < *τ*_*m*_, but the expansion can be extended to meet accuracy requirements by re-considering the criteria as:

30 $$ 1-{I}^T\mathit{\exp}\left({t}_f{A}_j\right).{P}^{(t)}\left({X}_0\right)\ge {\tau}_m\ast \frac{\left(\mathrm{no}.\mathrm{of}\ {R}_{M(sr)}\right)}{\left(\mathrm{no}.\mathrm{of}\ {R}_{M(fs)}\right)}, $$

31 $$ 1-{I}^T\mathit{\exp}\left({t}_f{A}_j\right).{P}^{(t)}\left({X}_0\right)\ge {\tau}_m\ast \frac{\left(\mathrm{no}.\mathrm{of}\ {R}_{M(fs)}\right)}{\left(\mathrm{no}.\mathrm{of}\ {R}_{M(sr)}\right)}, $$

32 $$ 1-{I}^T\mathit{\exp}\left({t}_f{A}_j\right).{P}^{(t)}\left({X}_0\right)\ge {\tau}_m(leak), $$

before steps to *t*_*f*_. However, the size of **X**_*K*_ obtained through Eqs. (30), (31) and (32) at *t*_*f*_ will be greater compared to the size of **X**_*K*_ obtained by Eq. (29) at *t*_*f*_, as the latter will have fewer states. In Eqs. (30), (31) and (32), with the increase in the size of *A*_*j*_, the value of the left-hand side will also increase, resulting in an improvement in Ӕ. When considering any Markov process of a biochemical system of any size in which the probability density expands according to Eq. (3) then Eqs. (30), (31) and (32) will approximate the solution within ${\tau}_m\ast \frac{\left(\mathrm{no}.\mathrm{of}\ {R}_{M(sr)}\right)}{\left(\mathrm{no}.\mathrm{of}\ {R}_{M(fs)}\right)}$, ${\tau}_m\ast \frac{\left(\mathrm{no}.\mathrm{of}\ {R}_{M(fs)}\right)}{\left(\mathrm{no}.\mathrm{of}\ {R}_{M(sr)}\right)}$ and *τ*_*m*_(*leak*), respectively, of the true solution of the CME, which is Eq. (3).

### Computational experimental results

The *ISP* method is initialized and parameterized using the initial conditions of the models. Due to a large number of mathematical operations and equations, simultaneous parameter predictions with a limited number of experimental values is often complicated for dynamic systems. Therefore, the consistency with the available experimental data was ensured at each step of the *ISP*. This method has led to the successful development of several functions that integrate large number of processes supporting extensive expansion of the state-space.

**Fig. 4:**
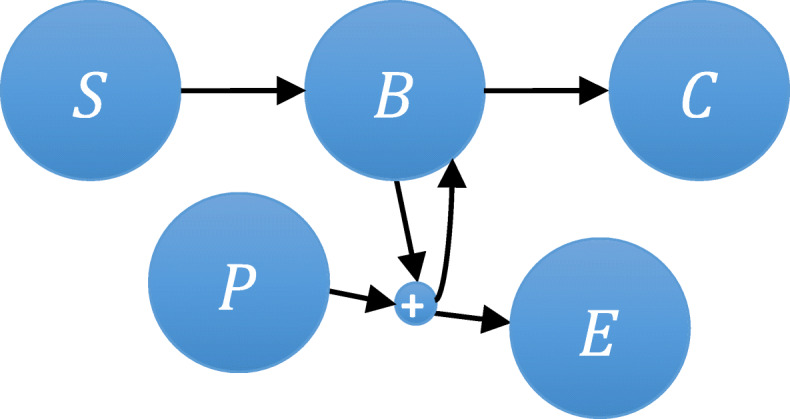
Catalytic reaction network with five $\widetilde{\mathrm{N}}=5$ species *S*, *B*, *C*, *P*, and *E* in a network defining reaction, as given in Eq. (33)

To demonstrate the *ISP LAS* algorithm, we first consider the catalytic reaction system [37] defined by the reactions

33 $$ S\ \overset{k_1}{\to }\ B\overset{k_2}{\to }\ C,\kern0.5em B+P\ \overset{k_3}{\to }\ B+E $$

depicted as a network in Fig. 4 as:

In this biochemical system (dimension = 5), reactant *P* will transform into product *E* via complex *B* when reactant *S* acts as a catalyst for the reaction and produces *C*. We rewrite this catalytic reaction system as a network of three reactions:

34 $$ {\boldsymbol{R}}_{\mathbf{1}}:S\overset{k_1}{\to }B, $$

35 $$ {\boldsymbol{R}}_{\mathbf{2}}:B\overset{k_2}{\to }C, $$

36 $$ {\boldsymbol{R}}_{\mathbf{3}}:B+P\overset{k_3}{\to }B+E; $$

with the initial copy counts *S*_0_= 50, *P*_0_= 80, *B*_0_ = *C*_0_ = *E*_0_ The reaction rate parameters are *k*_1_= 1, *k*_2_= 1000, *k*_3_= 100. These species counts are used as a state-space to define the model and these copy counts are tracked as $\left(\left[S\right],\left[B\right],\left[C\right],\left[P\right],\left[E\right]\right)\in \widetilde{N}:= \left({x}_0,{x}_1,{x}_2,{x}_3,{x}_4\right)$.

In reaction *R*_1_, the copy count of *S* is reduced by 1, which increases the copy count of *B* by 1. In reaction *R*_2_ the copy count of *B* is reduced by 1, which increases the copy count of *C* by 1. In contrast, reaction *R*_3_ decreases the *B* and *P* counts by 1 and increases the *B* and *E* counts by 1. As in *R*_3_, *B* acts as a catalyst to convert *P* to *E* and *B* is retained in the same reaction. We can now define the transitions associated with *R*_1_, *R*_2_, *R*_3_ in the stoichiometric vector *V*_*M*_ matrix as:

37 $$ {V}_M=\left[\begin{array}{c}{v}_1\\ {}{v}_2\\ {}{v}_3\end{array}\right]=\left[\begin{array}{c}-1\\ {}\ 0\\ {}\ 0\end{array}\ \begin{array}{c}\ 1\\ {}-1\\ {}\ 0\end{array}\ \begin{array}{c}0\\ {}1\\ {}0\end{array}\ \begin{array}{c}\ 0\\ {}\ 0\\ {}-1\end{array}\ \begin{array}{c}0\\ {}0\\ {}1\end{array}\right]. $$

For *LAS* method compatibility, the associated Markov chain of this model is converted into a Markov chain tree with the states in terms of the nodes with additional information such as the number of *R*_*M*_ reactions required to reach the state. In the growing Markov chain tree, the transition between the nodes:

38 $$ {N}_i\ \overset{v_{\mu}\left({X}_0(t),{X}_1(t),\dots .,{X}_K(t)\right)}{\to }\ {N}_{i+1}, $$

is defined in the typical form of the dictionary *Dict*. We express the propensity functions of the three reactions in terms of the states $\left(\left[S\right],\left[B\right],\left[C\right],\left[P\right],\left[E\right]\right)\in \widetilde{\mathrm{N}}$. Node *N*_1_ = (*X*_0_, ƌ_1_) carries the initial state *X*_0_ of the system at an initial depth of level 1. Further, **n**_*J*_ = (**X**_*K*_, ƌ_1, 2, …_) is expanded and the states updated by following the *LAS* order. The corresponding propensities *∆a*_*i*,*j*_ are updated in the *A*_*i*,*j*_ matrix in every iteration, based on the *LAS* updating trend (for example, see SI 2). The system began with *S*_0_= 50, *P*_0_= 80. Gradually all the reactants are transformed to products, *E* and *B*. The system ends in **n**_*J*_ = (*X*_1, 2, ……14666_, ƌ_*l*_).

**Fig. 5:**
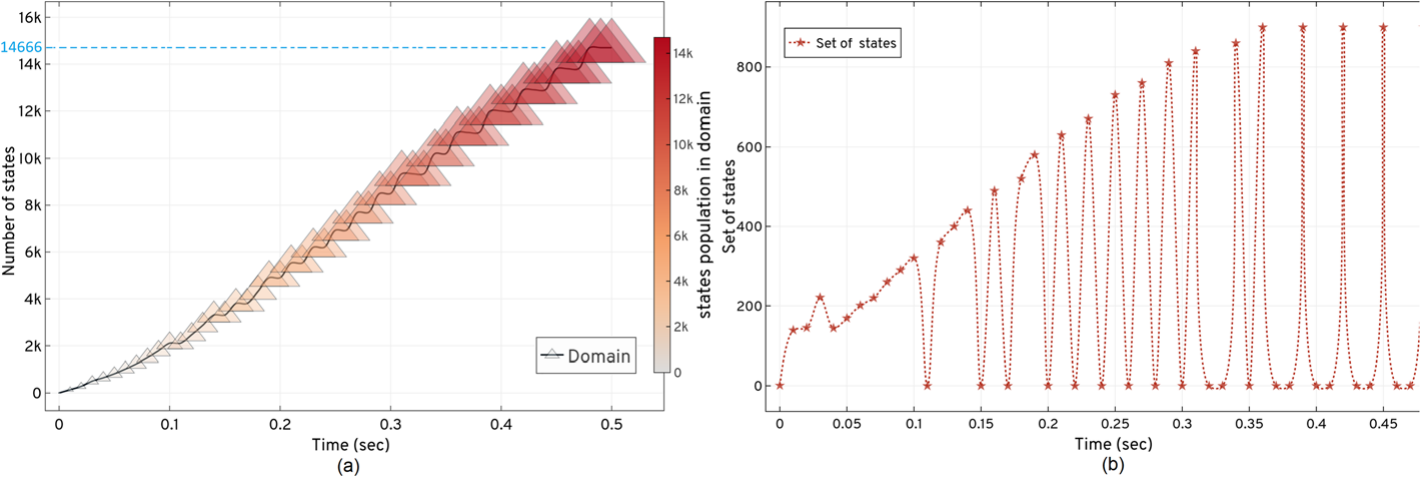
Expansion and updation of the states and set of states explored for the catalytic reaction system using the *LAS* method. (**a**) demonstrates that state-space expansion increases the number of new states in the domain. The size and colour of  shows the increase in the size of the domain with the states’ population. In (**b**), *LAS* unfolds the state-space pattern to update the states in the domain and expands 14666 probable states in 0.5 *secs*

Figure 5 shows the *LAS* method’s response when solved with *τ*_*m*_ = 1*e* − 6 for *t*_*f*_ = 0.5 *sec*. Due to the nature of the model reaction rates, small steps *t*_*step*_ = 0.01 *sec* are taken to capture the moments based on non-negative, non-zero states for the domain. *LAS* successfully creates the domain of an optimum order, with 14666 states at *t*_*f*_, by introducing the new states to the domain with time, as shown in Fig (a) in Fig. 5. This pattern demonstrates that the frequency (the number of states at any time *t*) of expansion increases in depth when the number of active reactions increase in the system. With the addition of probable states, the domain contains enough probability mass to approximate the solution up to *t*_*f*_. The states are updated in sets as seen in Fig (b) of Fig. 5, for the catalytic system after every iteration.

**Fig. 6:**
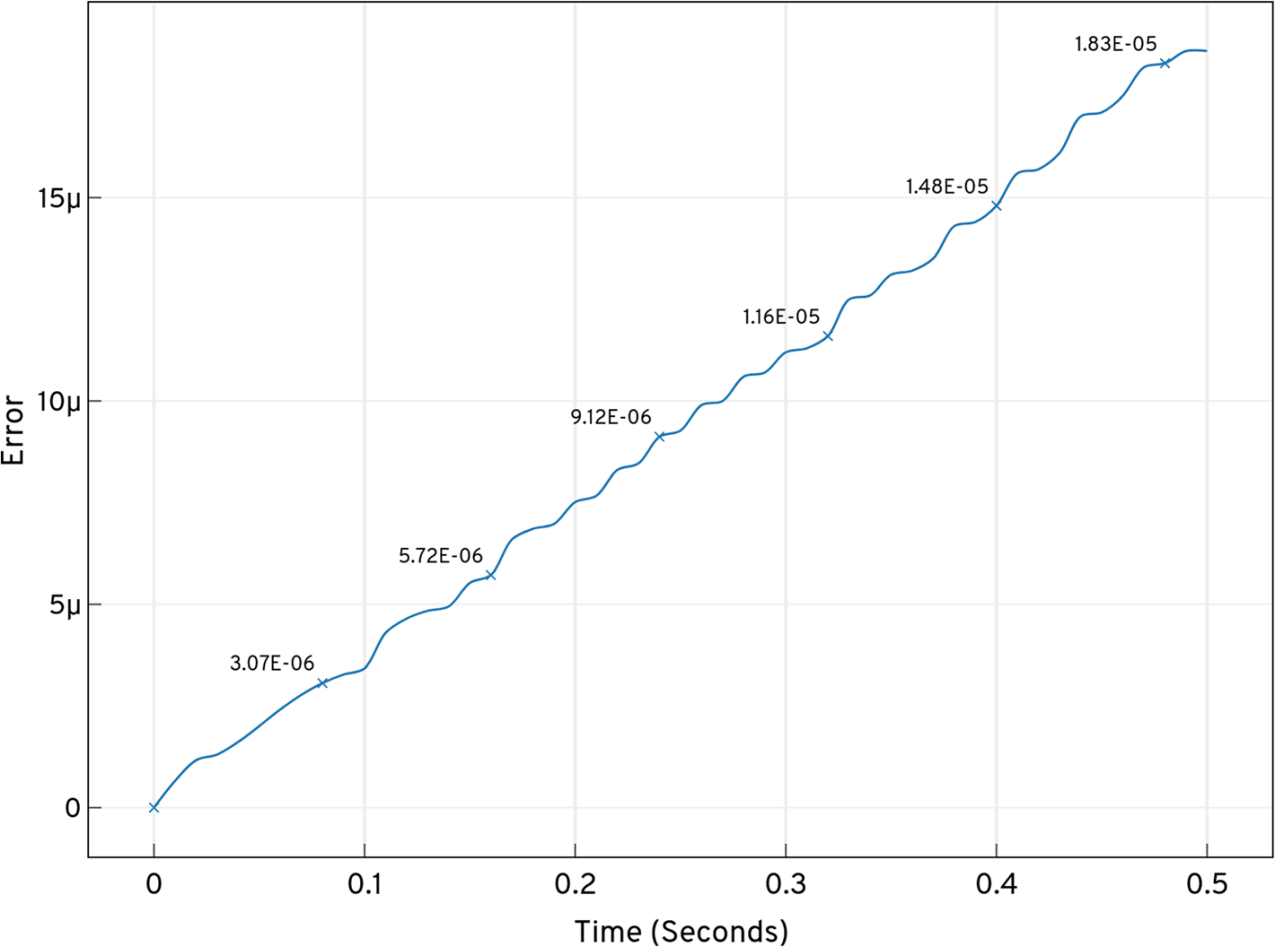
Total probability of states bunked at *t*^′^ from the domain of the catalytic system produced by *ISP LAS* iteration while expanding and solving the CME

The state-space pattern in Fig (b) of Fig. 5 can be used as a *blueprint* of the catalytic systems’ state-space to compare with other model’s *blueprints* for their characteristics and occurrence of reactions. Such a pattern can be used to predict the behavior of large network state-space expansions when the set of occurrences of the initial reactions are similar in different systems. The solution of Eq. (5), up to *t*_*f*_, for the domain created by *LAS*, is shown in Table 1. The system’s conditional probabilities based on its species are shown in Fig. 6.

**Table 1:** *LAS* expansion response and solution at *t*_*f*_ for the catalytic system

***t***_***f***_ ***=*** 0.5, ***t***_***step***_ ***=*** 0.01	Run-time (sec)	Domain	Expansion time (sec)	Error at ***t***_***f***_
***ISP LAS***	4677	14666	0.5	1.865e − 05

In three test runs, the *ISP LAS* run time for the catalytic system was 4677 *secs* when solving Eq. (5) with 14666 states. The probability of the species in Figure SI 17 (see SI 9) shows the nature of the reactions affecting each species’ count in the system. The involvement of species *B* in all the reactions results in its highest probability at *t*_*f*_. Species *B* also acts as a catalyst for *R*_3_, converting species *P* to *E*; therefore, both have equal probabilities at the time of solution.

Figure 6 shows the total probability bunked at *t*^′^ while progressing with the expansion. Bunking produces an error (w.r.t approximation), with time when the number of states increases with the expansion. *LAS* produces minimal error of order 10^−5^, as given in Table 1.

**Fig. 7:**
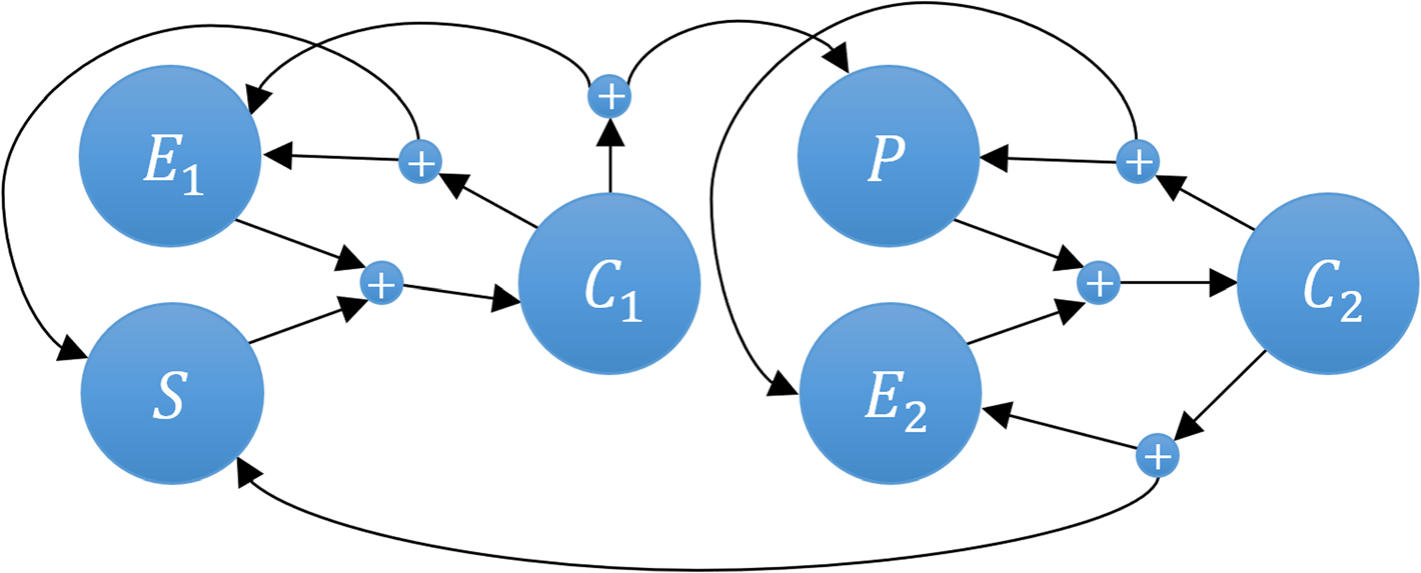
Coupled enzymatic reactions network. The figure shows six $\widetilde{\mathbf{N}}=\mathbf{6}$ species, ***S***, ***E***_**1**_, ***C***_**1**_, ***P***, ***E***_**2**_, ***C***_**2**_, in a network, defining reactions, as given in Eqs. (39) and (40)

To demonstrate the *ISP LOLAS* algorithm, we consider the coupled enzymatic reactions defined by the reactions

39 $$ S+{E}_1\overset{k_1}{\to }\ {C}_1\ \overset{k_2}{\to }\ S+{E}_1,\kern0.5em {C}_1\ \overset{k_3}{\to }\ P+{E}_1 $$

40 $$ P+{E}_2\overset{k_4}{\to }\ {C}_2\overset{k_5}{\to }\ P+{E}_2,\kern0.5em {C}_2\ \overset{k_6}{\to }\ S+{E}_2 $$

depicted as a network in Fig. 7 as:

This biochemical system (dimension = 6) describes two sets of enzymatic reactions transforming species *S* into species *P* and transforming species *P* back into *S*. We rewrite C reactions system as a network of six reactions:

41 $$ {\displaystyle \begin{array}{ll}{\boldsymbol{R}}_{\mathbf{1}}:S+{E}_1\overset{k_1}{\to }\ {C}_1,& {\boldsymbol{R}}_{\mathbf{2}}:{C}_1\ \overset{k_2}{\to }\ S+{E}_1\\ {}{\boldsymbol{R}}_{\mathbf{3}}:{C}_1\ \overset{k_3}{\to }\ P+{E}_1,& {\boldsymbol{R}}_{\mathbf{4}}:P+{E}_2\overset{k_4}{\to }\ {C}_2\\ {}{\boldsymbol{R}}_{\mathbf{5}}:{C}_2\ \overset{k_5}{\to }\ P+{E}_2,& {\boldsymbol{R}}_{\mathbf{6}}:{C}_2\ \overset{k_6}{\to }\ S+{E}_2\end{array}} $$

with initial copy counts *S*= 50, *E*_1_= 20, *E*_2_= 10, *C*_1_ = *C*_2_ = *P*= 0 and reaction rate parameters of *k*_1_ = *k*_4_= 4, *k*_2_ = *k*_5_= 5, *k*_3_ = *k*_6_= 1. These species counts are used as a state-space to define the model. These copy counts are tracked as:

$$ \left(\left[S\right],\left[{E}_1\right],\left[{C}_1\right],\left[P\right],\left[{E}_2\right],\left[{C}_2\right]\right)\in \widetilde{\mathrm{N}}:= \left({x}_0,{x}_1,{x}_2,{x}_3,{x}_4,{x}_5\right). $$

As in the previous example, we can now define the transitions associated with *R*_1_, *R*_2_, *R*_3_, *R*_4_, *R*_5_, *R*_6_ in the stoichiometric vector *V*_*M*_ matrix as:

42 $$ {V}_M=\left[\begin{array}{c}{v}_1\\ {}{v}_2\\ {}{v}_3\\ {}{v}_4\\ {}{v}_5\\ {}{v}_6\end{array}\right]=\left[\begin{array}{cccccc}-1& -1& 1& 0& 0& 0\\ {}1& 1& -1& 0& 0& 0\\ {}0& 1& -1& 1& 0& 0\\ {}0& 0& 0& -1& -1& 1\\ {}0& 0& 0& 1& 1& -1\\ {}1& 0& 0& 0& 1& -1\end{array}\right] $$

For the *LOLAS* method, the associated Markov chain of this model is converted to a Markov chain tree with the states in terms of nodes with additional information, such as the number of *R*_*M*_ reactions required to reach the state. In growing Markov chain tree, the transition between the nodes:

43 $$ {N}_i\ \overset{v_{\mu}\left({X}_0(t),{X}_1(t),\dots .,{X}_K(t)\right)}{\to }\ {N}_{i+1}, $$

is defined in the typical form of the dictionary *Dict*. We express the propensity functions of the six reactions in terms of the states $\left(\left[S\right],\left[{E}_1\right],\left[{C}_1\right],\left[P\right],\left[{E}_2\right],\left[{C}_2\right]\right)\in \widetilde{\mathrm{N}}$.

Node *N*_1_ = (*X*_0_, ƌ_1_) carries the initial state *X*_0_ of the system at the initial depth (level 1). Then **n**_*J*_ = (**X**_*K*_, ƌ_1, 2, ……_) is further expanded and the states updated by following the *LOLAS* order. The corresponding propensities *∆a*_*i*,*j*_ are updated in the *A*_*i*,*j*_ matrix in every iteration, based on the given *LOLAS* updation trend (for example, see SI 2). Initially, the system started with *S*= 50, *E*_1_= 20, *E*_2_= 10. Gradually all reactant species were transformed into products resulting in the system ending in **n**_*J*_ = (*X*_1, 2, ……8296_, ƌ_*l*_).

**Fig. 8:**
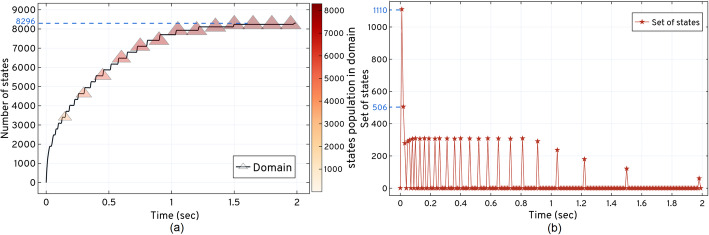
Expansion and updating of the states and set of states explored for the dual enzymatic reaction network using the *ISP LOLAS* method.  shows the increase in the domain size with the states’ populations.  shows the point in time where new set of states are explored and updated in the domain

Figure 8 shows the *LOLAS* method response when solved with *τ*_*m*_ = 1*e* − 6 for *t*_*f*_ = 2.0 *sec*. Due to the nature of the model reaction rates, small steps *t*_*step*_ = 0.01 *sec* are taken to capture the moments. These are based on non-negative, non-zero states for the domain. *LOLAS* successfully creates the domain of an optimum order with 8296 states at *t*_*f*_ by introducing the new states to the domain with time, as shown in Fig (a) of Fig. 8. In Fig (b) of Fig. 8, demonstrates that the frequency (the number of states at any time *t*) of expansion increases in depth when the number of active reactions increases in the system. With the addition of probable states, the domain contains enough probability mass to approximate the solution up to *t*_*f*_.

Fig (a) of Fig. 8 depicts state-space expansion which increases the number of additions of new states in the domain. In Fig (b) of Fig. 8, *ISP LOLAS* unfolds the state-space pattern to update states in the domain and expands 8296 probable states in 2.0 *sec*. As a blueprint of the dual enzymatic reaction network, the state-space pattern in Fig (b) of Fig. 8 can be compared with other model blueprints in terms of its characteristics and reactions. Such a pattern is considered to predict the behavior of a large network state-space expansion when the set of occurrences of the initial reactions are similar in different systems. The solution of Eq. (5), up to *t*_*f*,_ for the *LOLAS*-created domain is shown in Table 2. The system’s conditional probabilities based on species are shown in Figure SI 18 (see SI 10)

**Table 2:** *LOLAS*’ expansion response and solution at *t*_*f*_ for the dual enzymatic reaction network

***t***_***f***_ ***=*** 2.0, ***t***_***step***_ ***=*** 0.01	Run-time (sec)	Domain	Expansion time (sec)	Error at ***t***_***f***_
***ISP LOLAS***	1614.22	8296	2.0	5.953e − 05

In three test runs, *ISP LOLAS*^′^ run time for the dual enzymatic reaction network was ≈1614 *secs* when solving Eq. (5) with 14666 states. The probability of the species in Figure SI 18 (see SI 10) shows the nature of the reactions which affect each species’ count in the system. At *t*_*f*_, the probabilities of *E*_2_ and *C*_2_ remain high compared to *E*_1_ and *C*_1_ at different molecular counts. This results in a low probability of *P* compared to *S*. We know that this network transforms species *S* into species *P* and then transforms species *P* back into *S*. Based on the current probabilities of the species at *t*_*f*_, the future probability of *P* will increase. *S* will remain the same or decrease. With this change, the probabilities of *E*_2_ and *C*_2_ decrease in comparison to *E*_1_ and *C*_1_.

**Fig. 9:**
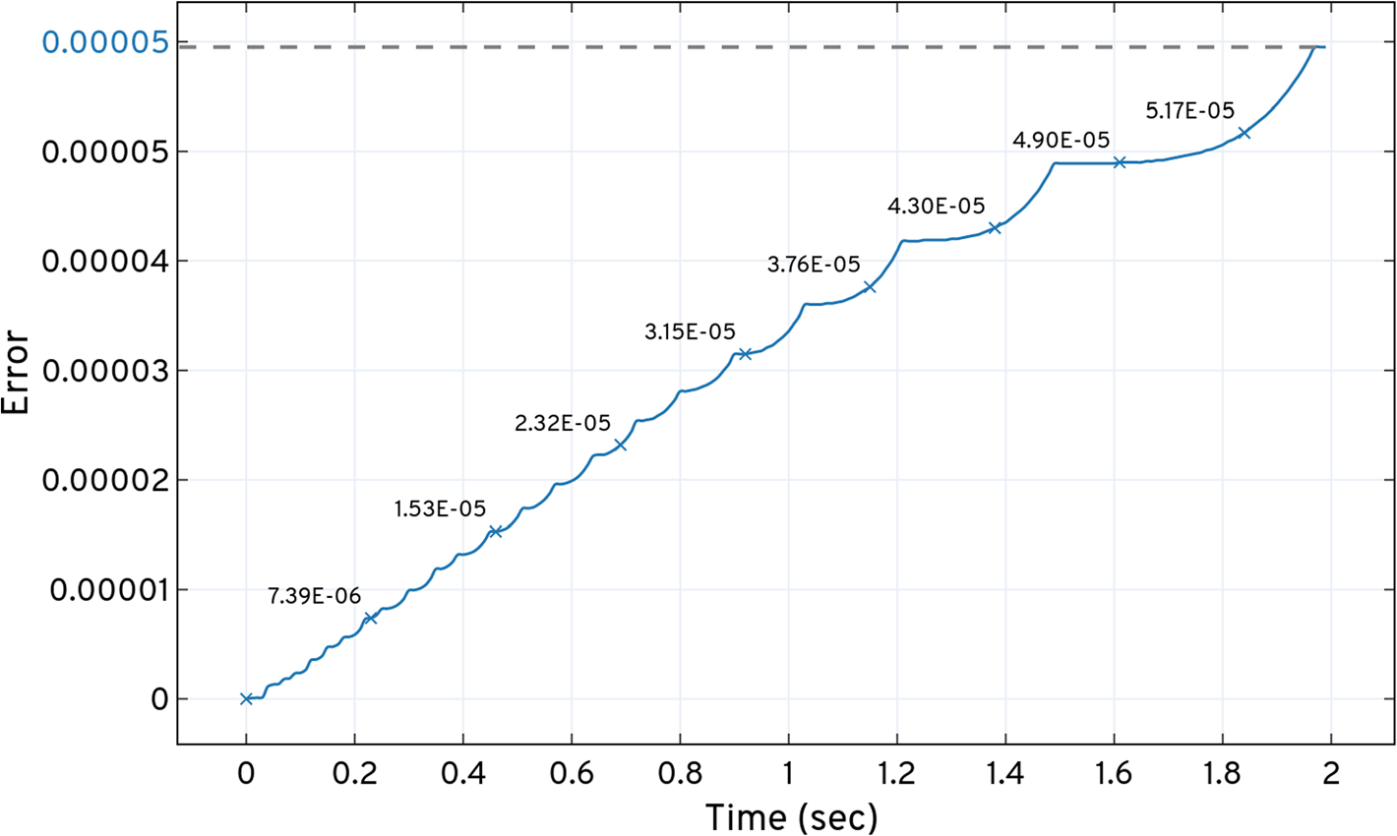
Total probability of states bunked at *t*^′^ from the domain produced by dual enzymatic reactions system in the *ISP LOLAS* iteration while expanding and solving the CME

Figure 9 shows the total probability bunked at *t*^′^ while progressing with the expansion. The bunking produces an error (w.r.t approximation) with time when the number of states increases with the expansion and provided that, *LOLAS* produces a minimal error of order, 10^−5^, as given in Table 2.

We extend the application of our *ISP* method to simulate a large model of the G1/S network [38] under the condition of DNA-damage. We want to determine the number of states at different points in time and predict the conditional probabilities of the protein species based on events leading to the formation of different complexes in the system.

The G1/S model (dimension = 28) with a DNA-damage signal transduction pathway is considered to be very stiff in nature, so while some molecular counts of certain proteins increase very rapidly others do so slowly. This makes it tough to solve, even for a short time period. The model is solved for *t*_*f*_ = 1.5 *sec* with ƃ_*limit*_ = 1, *τ*_*m*_ = 1*e* − 6, *t*_*step*_ = 0.1. The systematic exploration of nodes carrying probable states are undertaken in a similar way as discussed in Table SI 4 of SI 3 and depicted (see Figure SI 7 of SI 3) in six stages (denoted as $\hat{\mathrm{S}}$), representing *R*_*M*_ reactions with propensity, *a*_μ_, with the arcs as transitions.

The nodes are expanded up to *t*_*f*_ to enable identification of the reaction channels responsible for variations in the proteins. From the transitioning factor of the *2*^*nd*^*-tier*, we can see that every node has an average of at least ≈97 possible child nodes carrying states. Further, *Dict* is expanded for *n*-tiers of the child nodes to add more states to the domain. Additionally, **n**_*J*_ = (**X**_*K*_, ƌ_*l* = 1,2,……_) is expanded and updated, as per the *ISP LOLAS* trend (see Table SI 5 of SI 3).

**Fig. 10:**
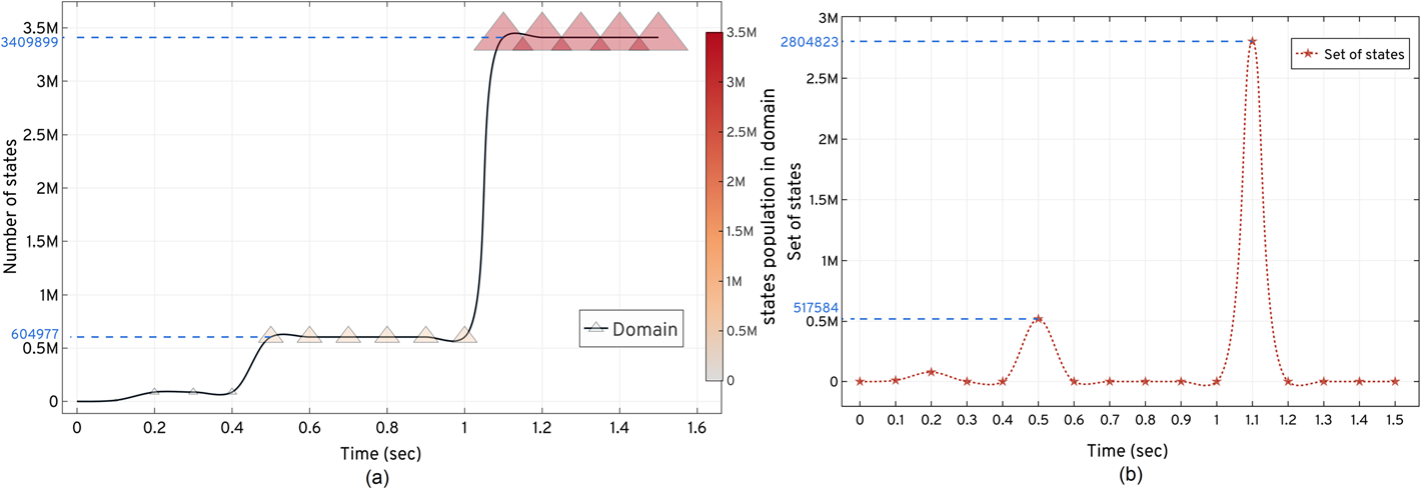
The expansion and updating of the states and set of states explored for the G1/S model based on the *ISP LOLAS* method.  shows the increase in the domain size with the states’ populations.  shows the point in time where new set of states are explored and updated in the domain

The *ISP LOLAS* method response for the number of states in the domain and time, *t*, is shown in Fig. 10. The initial response suggests that only a few reactions were active until *t*= 0.4 *sec.* After that time, more reactions triggered that explosively take the exploration above 0.5 million states in 0.5 *sec*. For such a large model, this combination of explosion states was expected as proteins undergo several excursions due to the number of reactions in fractions of time, *t*. The second explosion of states occurs after 1.0 *secs* when almost all the reactions (involving the species, given in SI 4.1) become active in the network. The size and colour of the *2D pyramid* in Fig (a) of Fig. 10 shows the increase in the size of the domain with the state explosions. The number of sets of states that create the bounds at *t* are shown in Fig (b) of Fig. 10. With the exploration of the set of 517584 states, the *Bound*(3)_*upper*_ = {*X*_0, 1, 2…..604677_} is formed at 0.5 *sec* carrying 604677 states. Some states were bunked at 0.5 *secs* resulting in approximation errors that reach 2.42e − 06 at 0.6 *sec*. At *t*_*f*_, the *LOLAS* ends up with a domain defined by *Bound*(4)_*upper*_ = {*X*_0, 1, 2…..3409899_} carrying 3409899 states with 3.52e − 06 approximation errors.

Fig (a) of Fig. 10 demonstrates that the state-space expansion increases the number of additions of new states in the domain. *ISP LOLAS* quickly expands the state-space up to ≈3.5 million states in 1.5 *secs*. In Fig (b) of Fig. 10, *ISP LOLAS* unfolds the state-space pattern to update states in the domain and expands 3409899 states up to *t*_*f*_.

The corresponding propensities, *∆a*_*i*, *j*_, are updated in the *A*_*i*, *j*_ matrix in every iteration, based on the *ISP LOLAS* update trend. The system started with the initial state of the protein species and gradually, as protein levels change in the system, it exploits the copy counts that shift the system to a new state. The change in protein levels causes the system to transform into new states: here we see the manifestation of the Markov process of the system. The *ISP LOLAS* captures this process and defines several bounds of the domain at different time intervals, as indicated by the pyramid in Fig. 3. To investigate the expansion of states more closely, the order of bounds at different time intervals, and the number of states present in the bounds, are provided in Table 3. The size of bound created in each duration reveals that for every step, the growth of the domain is eight-to-ten times the previous size of the domain.

**Table 3:** Lower and upper bounds of the domain for the G1/S model given by the *ISP LOLAS* trend based on bound limit *ƃ*_*limit*_

***Z***	***Bound***(*Z*)_***lower***_	***Bound***(*Z*)_***upper***_	States	Duration
1	*Bound*(1)_*lower*_ = {*X*_0_} formed at *t*= 0.0 *sec* *Approximation* = 1	*Bound*(1)_*upper*_ = {*X*_0, 1, 2…..9808_} formed at *t*= 0.1 *sec* *Approximation* = 0.999999867	9808	0.0 – 0.1 *sec*
	*ƃ*_*limit*_ = 1, *count*(*ƃ*_*limit*_) = 0, 1,			
2	*Bound*(2)_*lower*_ = *Bound*(1)_*upper*_ formed at *t*= 0.1 *sec* *Approximation* = 0.999999847	*Bound*(2)_*upper*_ = {*X*_0, 1, 2…..87393_} formed at *t*= 0.2 *sec* *Approximation* = 0.999999173	87393	0.1 – 0.2 *sec*
	*ƃ*_*limit*_ = 1, *count*(*ƃ*_*limit*_) = 0, 1			
3	*Bound*(3)_*lower*_ = *Bound*(2)_*upper*_ formed at *t*= 0.4 *sec* *Approximation* = 0.999999157	*Bound*(3)_*upper*_ = {*X*_0, 1, 2…..604677_} formed at *t*= 0.5 *sec* *Approximation* = 0.99999701	604677	0.4 – 0.5 *sec*
	*ƃ*_*limit*_ = 1, *count*(*ƃ*_*limit*_) = 0, 1			
4	*Bound*(4)_*lower*_ = *Bound*(3)_*upper*_ formed at *t*= 1.1 *sec* *Approximation* = 0.99999699	*Bound*(4)_*upper*_ = {*X*_0, 1, 2…..3409899_} formed at *t*= 1.5 *sec* *Approximation* = 0.99999648	3409899	1.1 – 1.5 *sec*
	*ƃ*_*limit*_ = 1, *count*(***ƃ***_*limit*_) = 0, 1			

The set of nodes *N*_1_, *N*_2_, . . …*N*_3409900_ carries unique states representing the set of *state*(*n*_3409900_) = (*X*_0, 1, 2, …3409899_) that forms the state-space of the model. It is important to note that some proteins are synthesized and promoted by the network itself, as evidenced by some reactions of the pathway, which increase the frequency of the repeated states. However, *ISP LOLAS* validation does not consider them for the domain. Equation (5)’s solution, up to *t*_*f*_ for the domain, created by *ISP LOLAS*, is shown in Table 4.

**Table 4:** *ISP LOLAS*’ expansion response and solution at *t*_*f*_ for the G1/S model

***t***_***f***_ ***=*** 1.5 ***sec***, ***t***_***step***_ ***=*** 0.1	Run-time (sec)	Domain	Expansion time (sec)	Error at ***t***_***f***_
***ISP LOLAS***	1372	3409899	1.5	3.52e − 06

**Fig. 11:**
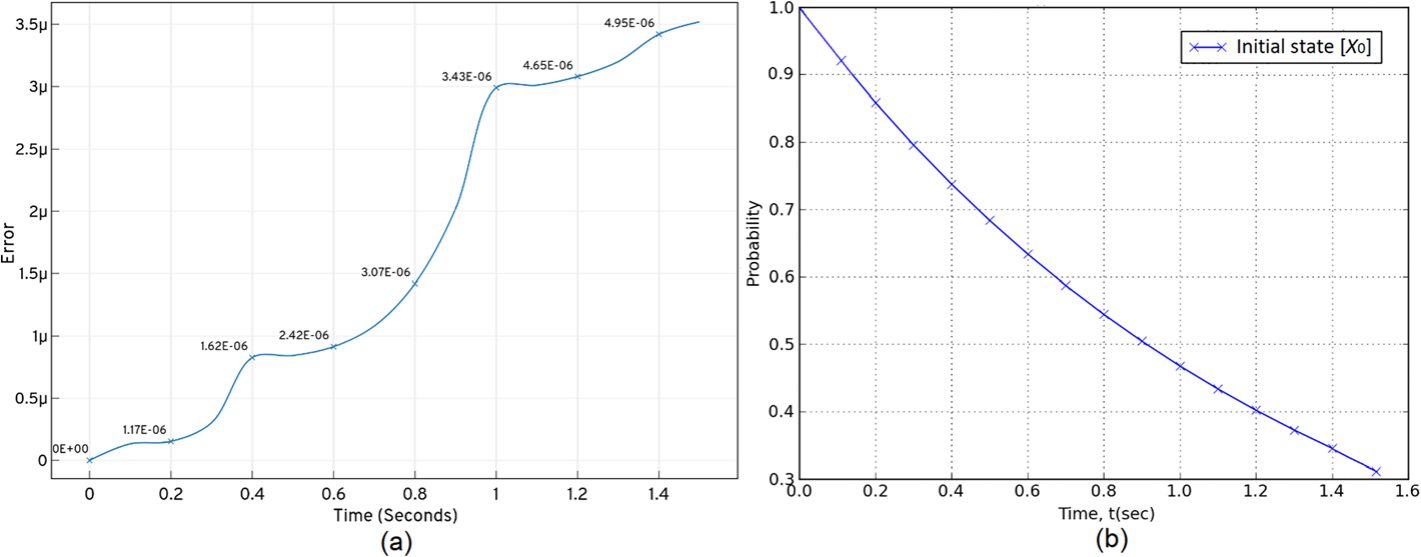
The *ISP LOLAS*′ response for total probability bunked at ***t***^′^ from the domain and checkpoint for examining the initial state probability over time. (**a**) shows how ***ISP*** maintains accuracy by keeping low errors. (**b**) shows the decline in the probability of the system remaining in the initial state in the presence of DNA damage

Over three test runs, the *ISP LOLAS*′ run time for the G1/S model was 1372 *secs* for solving Eq. (5), with the optimal domain having 3409899 states. The *ISP LOLAS* response given in Fig. 11, shows the system’s probabilities bunked at *t*^′^ during the expansion (w.r.t approximation), when the number of states increases with the expansion, and provided that *ISP LOLAS* produces minimal errors of the order of 10^−6^, as given in Table 4 and Fig (a) of Fig. 11. We set the checkpoint to examine the initial state’s probability over time. The response in Fig (a) of Fig. 11 indicates that the probability of the system remaining in the initial (normal) state decreases with time in the presence of DNA damage, which triggers the change in protein levels.

The conditional probabilities of the species’ systems are given in Fig. 14 and SI 8. In the case of DNA-damage, large numbers of the most notable parameters increase, compared to normal conditions (in cell cycle progression). The increase is predominantly related to *x*_14_ (p21) having a high initial probability, see Fig(14) of Figure SI 19 (see SI 11). The feedback (negative) of *x*_24_ (p53) increases its probability, see Fig(24) in Figure SI 19 (see SI 11), such as the association rate of *x*_16_ (p21/CycE/CDK2 − P), the rate of synthesis of *x*_14_ (p21) by *x*_24_ (p53), the rate of degradation of *x*_14_ (p21), and the rate of synthesis of *x*_24_ (p53) by DNA-damage signal. The conditional probabilities of the two key proteins, *x*_10_ (p27) and *x*_1_ (CycE), are affected by the change in the cell’s response to the level of the DNA-damage signal, see Fig (10) and Fig (3) in Figure SI 19 (see SI 11). The parameters related to *x*_10_ (p27), as well as *x*_1_ (CycE), greatly affect the probability of *x*_21_ (E2f) with time, see Fig (21) in Figure SI 19 (see SI 11). The impact of *x*_1_ (CycE) involves additional parameters related to CycA, because the release of supplementary *x*_21_ (E2f) depends on *x*_20_ (Rb − PP/E2f) hyperphosphorylation by the activation *x*_7_ (CycE/CDK2 − P), which affects the probability of *x*_21_ (E2f).

When the release of *x*_21_ (E2f) is affected, the probability of *x*_1_ (CycE) increases, see Fig (3) in Figure SI 19 (see SI 11). This leads to the progression to the S-phase, followed by the temporary suspension of cell cycle progression. The increase in probability of *x*_24_ (p53) shows cell support to repair the DNA damage. The parameters and the probabilities relating to *x*_14_ (p21) and *x*_24_ (p53) become important in the case of DNA damage. When combined, the conditional probability of these parameters indicates the involvement of the DNA-damage signal in the transition of G1/S.

## Discussion

In this section, we discuss *ISP* performance, focusing specifically on the speed and accuracy of the expansion, domain size and accuracy of the solution in comparison with other methods.

### Comparison with other methods

An approximation of 10^−5^ is used to find the approximate number of realizations required by the *SSA* for the 10^−4^ global error. Realizations were computed until the difference was less than 10^−4^ between the known distribution and the empirical distribution.

Approximately 10^6^ and 10^5^ runs were required to obtain the right distribution for the catalytic and dual enzymatic reaction networks, respectively. In the catalytic system, we observe (see Table 5) that both versions of *ISP* are faster than the *OFSP* of *r-step reachability* and the *SSA* of sliding windows. We attribute this greater efficiency to *LOLAS* having fewer states and less computational time than the *OFSP* method. *LOLAS* has better accuracy at *t*_*f*_. Similary, the *ISP* was much faster than the *SSA*, and the total number of realizations required from the *SSA* to have an error at *t*_*f*_ still large than that of *LOLAS* is 10^6^. In the dual enzymatic network, we observe (see Table 5) that both versions of *ISP* are faster than the *OFSP* of *r-step reachability* and the *SSA* of sliding windows; we attribute the improvement to both *ISP* variants having an efficient domain with a small approximation error and less computational time than that of the *OFSP* method and better accuracy at *t*_*f*_. Similarly, both *ISP* variants were much faster than *SSA*, as the total number of realizations required to have an empirical distribution with the error at *t*_*f*_ is ≈12 times more than the domain produced by *ISP*.

**Table 5:** Comparison of the solution of the catalytic reaction system based on *ISP*, *OFSP* and *SSA*

***t***_***f***_ ***=*** 0.5, ***t***_***step***_ ***=*** 0.01	***ISP***		***OFSP***	***SSA***
	***LAS***	***LOLAS***		
**Catalytic reaction system (*t***_***f***_ ***=*** **0.5,** ***t***_***step***_ ***=*** **0.01)**				
**Run-time (sec)**	4677	2706	8767	17428
**Domain at** ***t***_***f***_	14666	13089	14665	10^6^ Runs
**Expansion time**	0.5	0.5	0.5	-
**Error at** ***t***_***f***_	1.865e − 05	1.532e − 05	1.917e − 05	≈9.81 x 10^−3^
**Dual enzymatic reaction system (*t***_***f***_ ***=*** **2.0,** ***t***_***step***_ ***=*** **0.01)**				
**Run-time (sec)**	2386	1614	2804	6374
**Domain at** ***t***_***f***_	8282	8296	8266	10^5^ Runs
**Expansion time**	2.0	2.0	2.0	-
**Error at** ***t***_***f***_	7.470e − 05	5.953e − 05	1.060e − 04	≈9.94 x 10^−3^

**Fig. 12:**
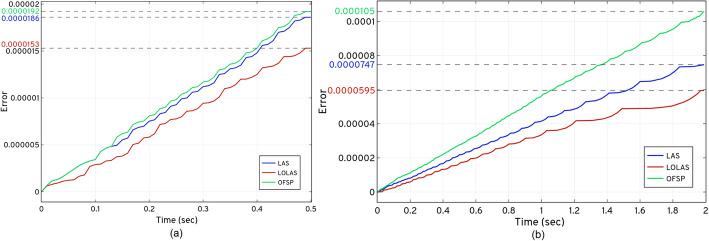
Comparing *ISP* (*LAS* and *LOLAS*) with *OFSP*, based on the solution of the catalytic and dual enzymatic reaction networks

We also compared the error at *t*_*f*_ to determine the solution’s efficiency. As seen in the results, the increase in the step error in *OFSP* affected the solution at *t*_*f*_. Figure 12 (see Fig (a) and (b)), compares the *ISP* (*LAS* and *LOLAS*) with *OFSP* on the basis of the approximation error at *t* during the expansion of the catalytic and dual enzymatic reaction networks, respectively. Addressing the step error in *ISP* and the selection of the probable states results in a more efficient solution at *t*_*f*_ compared to *OFSP*.

**Fig. 13:**
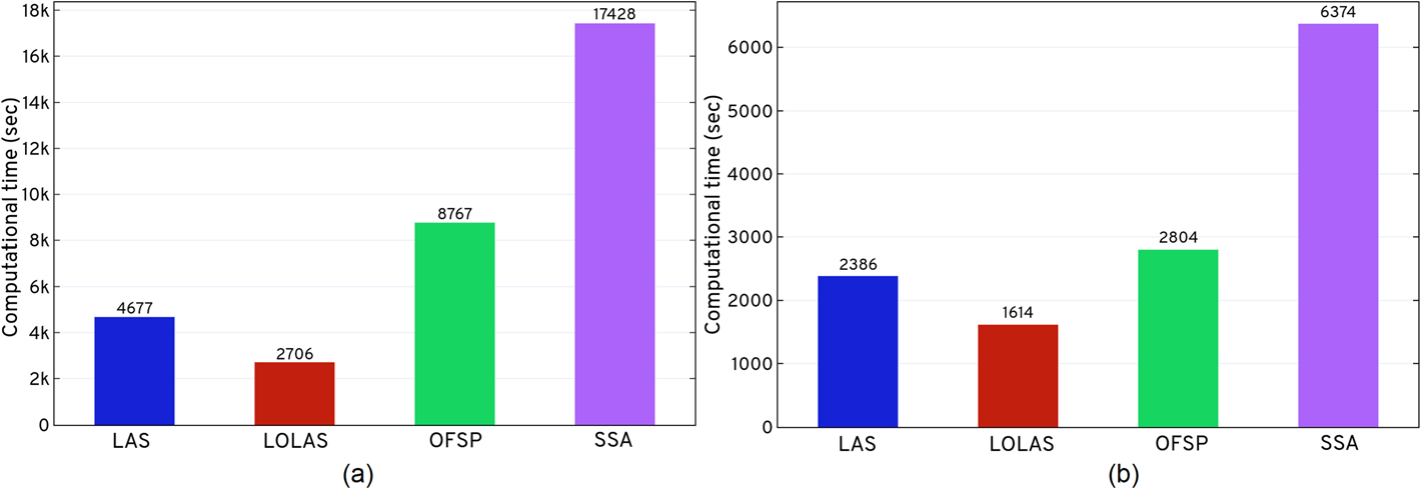
Comparing computational time for *ISP* (*LAS* and *LOLAS*) with *OFSP* and *SSA* by computational time. All methods were applied to the catalytic and dual enzymatic reaction networks that were previously integrated in the experimental results section

The typical firing nature of reactions in the catalytic system makes them stiff. Therefore, the selection of states becomes difficult to approximate. While some species in the system increase abruptly, others do so very slowly because the kinetic parameters (*k*_1_= 1, *k*_2_= 1000, *k*_3_= 100) have large differences: this triggers reactions at different rates. Reaction *R*_1_, is categorized as a *slow* reaction in the network: it affects the fast reaction, *R*_2_. As the computation results of Table 5 show, the *ISP* found that only 13089 probable states were required to solve the system up to *t*_*f*_. This not only saves computational time (see Fig. 13) compared to *OFSP* and *SSA*, and improves the solution’s accuracy. In *OFSP*, applying the compression at every step or after a few steps is still computationally expensive for a model like the catalytic reaction system, as seen in Table 5 and Fig (a) of Fig. 13.

The network shown in Fig. 7 consists of two interlinked enzymatic reaction systems. These systems transform species *S* and *P* into each other via the other species, making the system stiff in nature. The selection of states thus becomes difficult for approximation. This is due to some species (*S* and *E*_1_) in the system increasing abruptly, while others take longer to increase. Some of the kinetic parameters (*k*_1_ = *k*_4_= 4, *k*_2_ = *k*_5_= 5) have large differences from other kinetic parameters (*k*_3_ = *k*_6_= 1): this triggers reactions at different rates. Categorized as the fastest reaction in the network, R affects species S, C, E. It is followed by other reactions involving other species. As the computation results in Table 5 show, the *ISP LAS* indicated that only 8282 probable states are required to solve the system up to *t*_*f*_. Likewise, *ISP LOLAS* identified that only 8296 probable states are required to solve the system up to *t*_*f*_ This saves computational time (see Fig. 13), compared to *OFSP* and *SSA*, and improves the solution’s accuracy. In *OFSP*, applying the compression at a defined step or after a few steps is still computationally expensive for models like the dual enzymatic reaction system, as seen in Table 5 and Fig (b) of Fig. 13.

The total computation effort required at every step, when compressing the number of states up to *t*_*f*_, is approximately equal to the total computation effort required when the compression is applied in the gaps in some steps on a set of states up to *t*_*f*_. Moreover, the state-space will remain the same at *t*_*f*_ , regardless of when the compression is applied.

**Fig. 14:**
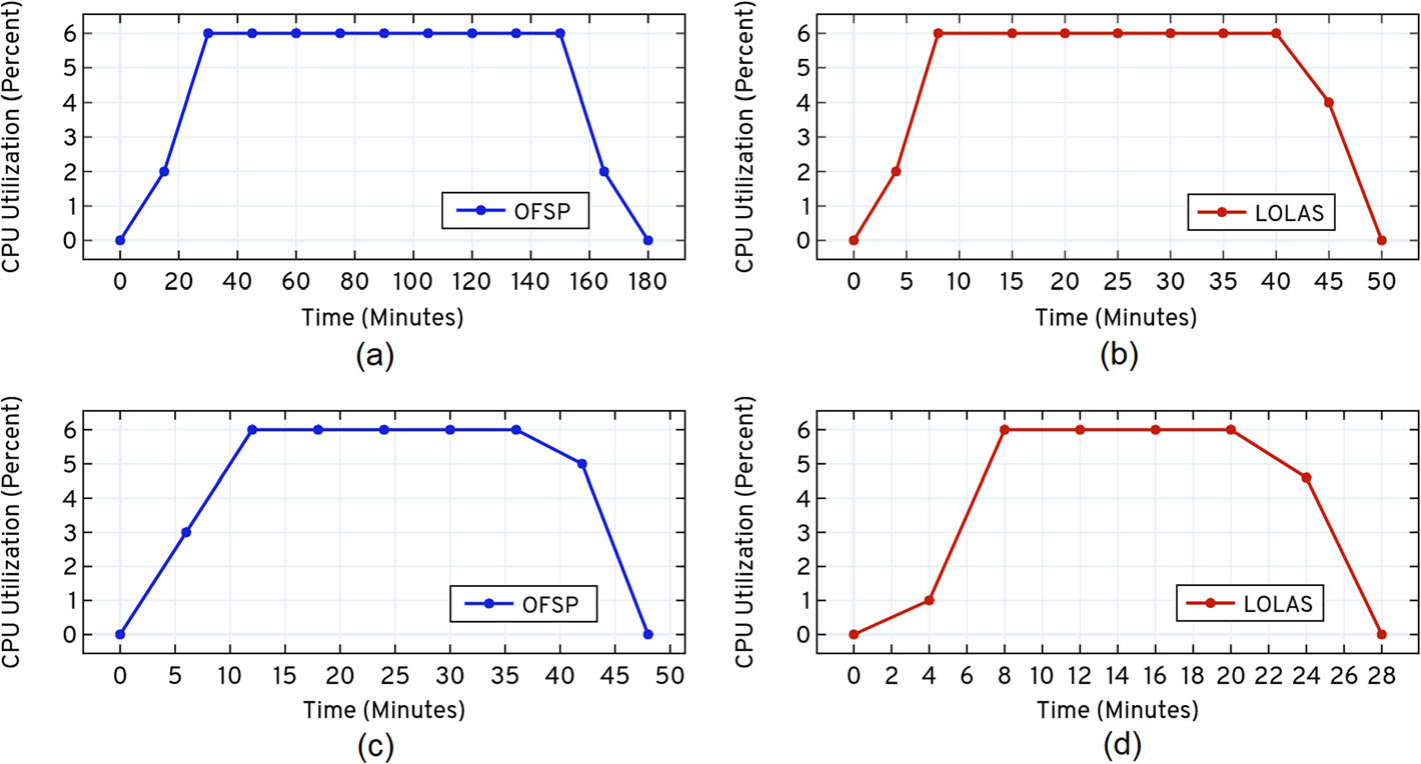
AWS® CPU utilization percentage, when the catalytic reaction system is solved up to *t*_*f*_= 0.5 *sec*, and the dual enzymatic reaction system solved up to *t*_*f*_= 2.0 *sec*, using *OFSP* and *LOLAS*. The performance analysis was carried out using CloudWatch® (Statistic: Average, Time Range: Hour, Period: 5 Minutes)

A comparison of the computational times in Table 5 shows that both versions of *ISP* are significantly faster than other methods. Figure 14 shows the CPU utilization (%) of *LOLAS* and *OFSP* with respect to run-time (minutes). The dedicated throughput (see SI 1.1) between EC2 and EBS was not used to solve the model. The average CPU exertion is about 60%, which is a considerable workload for a given model. The expansion and approximation began when CPU use was at ≈1.6422% in the catalytic reaction system and ≈1.23% in the dual enzymatic reaction system, at *t*= 0 *sec.* It increases up to 60.0% and then drops down to zero at *t*_*f*_.

### Theoretical interpretation of methods

Although, *SSA* recognizes the support and wastes no time in searching for the right domain and creating independent realizations which can be run parallel on multi-core environment, solving the system via Eq. (5) is quicker than creating realizations via stochastic simulation [39–41]. This is because the *N-term approximation* [42, 43] of the probability distribution to create the required number of realizations is always less than, or equal to, the minimal support approximation up to same error. These realizations were computed until the difference was less than the prescribed approximation between the known distribution and the empirical distribution. In terms of the system dimension, which is usually defined by the number of species in the system, the approximation of Eq. (5) in *ISP* becomes smaller problem to solve compared to approximation through *SSA*. This enables *ISP* to perform better.

In contrast, *OFSP* creates a hyper-rectangle and applies truncation to guarantee the minimal order domain for the approximation. *OFSP* truncates the state space after every few steps to ensure the minimum size of the domain and enable greater computational speed. However, differences in reaction firing changes the probability of some states at a later stage; therefore, truncating the state space in *OFSP* after every few steps or at every step would remove probable states from the domain, which in turn would affect the accuracy of the solution. As a result of this, *OFSP*^′^*s* overall performance is compromised. In contrast, *ISP* first explores the states based on guided exploration through the *BLNP* function (see method section (a)) and then leaks the set of states ${\mathbf{X}}_K^{\prime }$ which have the lowest probabilities in the bunker at *t*^′^ without removing them (see Eq. (20)). It recalls these sets of states when the probabilities of these states increase at later time.

In *ISP*, the time and space complexity (refer to SI 7) of removing and accessing the states in the bunking and recalling process is optimum, compared to the overall time and space complexity of the truncation step in *OFSP* [20]. As seen in Table 5, the number of states present in the domain for the catalytic reaction network in *ISP LAS* is approximately equal to number of states present in the domain produced by *OFSP*. Additionally, the number of probable states in the domain for the dual enzymatic reaction system in *ISP LAS* is quite more as compared to the domain produced by *OFSP*. However, better complexity and the guided selection of probable states for the domain produces low approximation errors and means that *ISP LAS* performs better overall than *OFS*. Similarly, *ISP LOLAS* outperforms *OFSP* in finding the optimum domain due to its bi-directional exploratory nature (see methods section (c)). This feature helps *ISP LOLAS* to achieve a more accurate solution (see Table 5 and Fig. 12) as well as a quicker computational time (see Fig. 13). These benefits are also due to fact that *ISP* visualizes the state-space as a Markov chain graph or a tree (see Markov chain as a Markov chain tree section) which ultimately decreases the complexity in the expansion phase.

## Conclusions

This paper has introduced a novel approach, *ISP*,to model biochemical systems. This new approach addresses both performance and accuracy problems in CME solutions. Provided all probable states are not added into the domain, up to the desired *t*_*f*_, variants of *ISP* (*LAS* and *LOLAS*) provide systematic ways of expanding the state-space. We have demonstrated the effectiveness of our methods with several experiments using real biological models: the catalytic reaction system, the dual enzymatic reaction system, and the G1/S model (large model). The results and the algorithm’s responses reveal improvements in how different sized biological networks can be modeled: even state-spaces with 3409900 nodes (see Table 3) carrying states up to ≈3.5 million can be explored within a reasonable time. The results also show that the domain laid out by *ISP* had an optimal order and was successful in finding probable system states, all the while maintaining high levels of accuracy and efficient computational timing.

We have compared the *ISP* results against two popular methods: *OFSP* (*r-step reachability*) and *SSA* (*τ* leaps adaptive). *ISP* outperformed the other methods, in computational expense, accuracy and projection size. The *ISP* was more effective in terms of predicting the behavior of the state-space of the system and in performance management, which is a vital step in modeling large biochemical systems. Unlike other methods, the *ISP* keeps the lowest states probabilities in the bunker without removing (as removed in *OFSP*) them, before calculation (as removed without calculation in *FSP GORDE*). It computes the probabilities at *t* without computing large numbers of realizations (as done in *SSA*).

The diverging nature of the *ISP* response, with respect to *OFSP* in Fig. 19, also shows that the solution improved with *t* and at a higher *t*_*f*_. For example, in the large model (case study 2), the computation time was 1372 *sec* and the solution was 3.52e − 06 at *t*_*f*_, which was lower than the small model results (the catalytic reaction system). These results show *ISP*’s compatibility with the distinct size of biochemical models.

These examples have demonstrated that *ISP* is a very promising technique for system’s biology. For stiff models, such as the G1/S and *Candida albicans* models, the *ISP* yielded plenty of information. Likewise, it provided opportunities for stochastic analysis of large models. *ISP* can be used to compute the probabilities of the species up to the required time. One could also use *ISP* to conduct *robustness* and *sensitivity analysis* on the dynamics of biochemical systems and to keep track of what reactions are more active in the system at a particular time. *ISP* is also able to determine the complexity of the system by defining the bounds with number states and keep track of the nested state-space patterns (called the *ISP* model blueprint) that were updated at the end of each step. Outlining the patterns of expansion of states to predict the projection folds can be used for updating the new states.

We anticipate that the current structure of the *ISP* variants can be employed for different classes and varieties of biological systems. Additionally, they can be used to compute the configurations with many reactions, as long as the notable part of the state-space density is present between *Bound*(*Z*)_*lower*_ and *Bound*(*Z*)_*upper*_. When there was a high probability of the molecular population of the species undergoing several excursions in a fraction of time, then the *ISP* uses a small *t*_*step*_ to capture these moments. While such computations were still challenging in the expansion phase for typical models they can be addressed more closely in combination with the second part of the CME solution: that is, the approximation phase. There are several methods which can be used to address these challenges.

Approximation methods, such as the *Krylov sub-space*, can be used to effectively compute the matrix exponential times of a vector. While it was mathematically attractive to aggregate the states or decompose the large sparse matrix into a small dense matrix using the *Krylov sub-space,* this method may not be computationally efficient in the absence of an efficient domain. Performance can also be enhanced by employing the fast math functions, compatible with the multicore environment. We have clearly outlined the core ideas behind the *ISP* variants. We have highlighted the differences and similarities between them and other methods that cover the computational and theoretical considerations that are essential before any of the approximation methods becomes feasible for an efficient CME solution.

## Methods

**Fig. 15:**
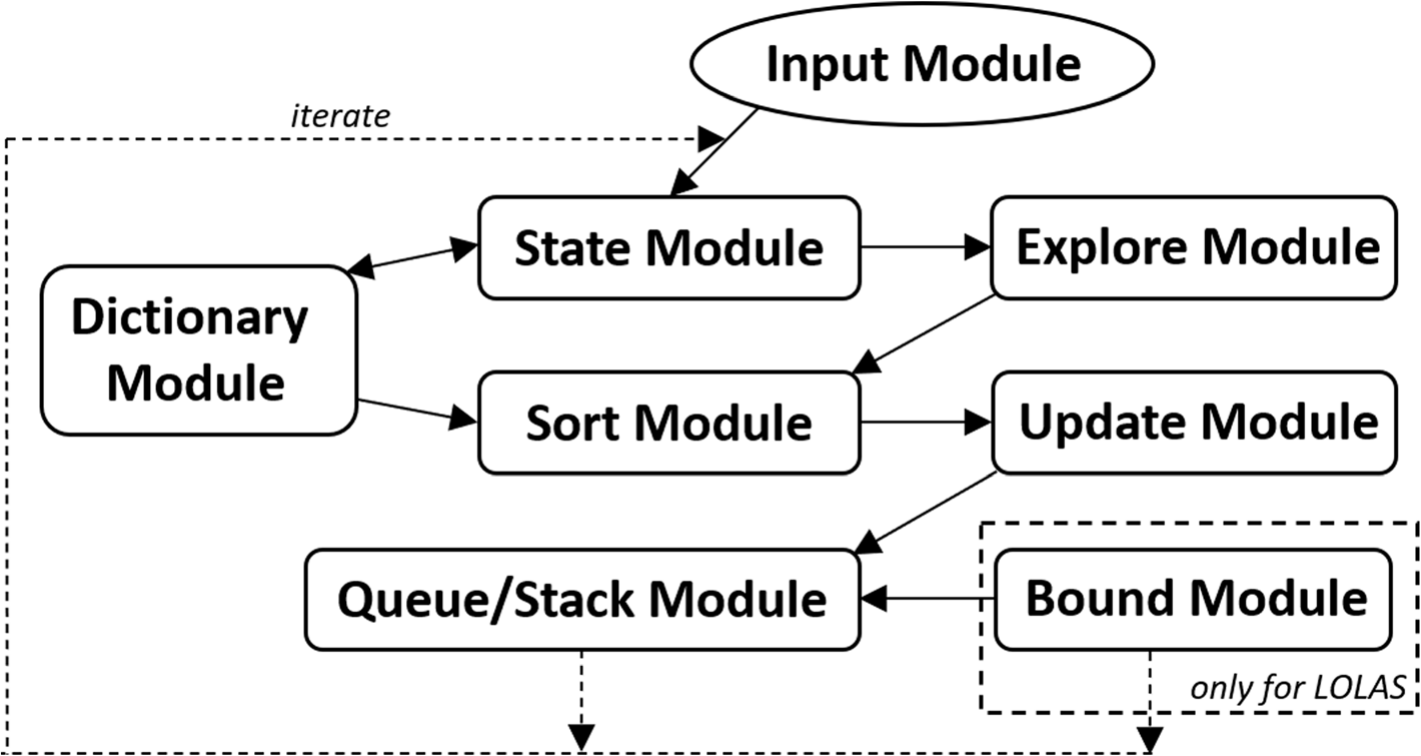
Comprehensive *ISP* method flow chart. A description of the modules (*steps*), sub-modules and the list of components are discussed in SI 5

To understand and predict the dynamics of the state-space response in biochemical systems, we have developed an analytical numerical method called *ISP*. This method integrates the reactions’ propensities describing the Markovian processes through set of nodes governing set of states of the system. The two variants of *ISP*, named *LAS* and *LOLAS*, consist of several modules that incorporate sets of inputs and functions within several compartments. Figure 15 depicts all the *ISP* modules. The integrated form is discussed later in Tables 7 and 8.

These modules and sub-modules constitute the *ISP* method. They track key changes in the components that follow changes in the reaction propensities by population and activation of the species. The modules also describe the dynamics of the biochemical system. The method also permits the time form quantification of state-space based on the size and model dimension.

The *ISP* states expansion strategy is based on the *Artificial Intelligence* (*AI*) standards [44–47], state-space search and relative successor (*S*_*uc*_) operator or function which perform operations on inputs. *AI* refers to the study of intelligent agents [48] of a system that perceives and takes action to successfully achieve goals. Most of the problems can be formulated as searches. They can be solved by reducing to one of searching a graph or a tree. To use this approach, we must specify the successor operator which defines the sequence of actions leading from initial to goal state at different time intervals, that lead to the solution.

In terms of *AI*, we define the *state-space* as a set of states in the system we can get to by applying *S*_*uc*_ to explore new states of a biochemical network. *S*_*uc*_ can be applied explicitly, which maps the current state to the output states, or defined implicitly, in that it acts on the current state and transforms it into a new state. In the *state-space* graph for biochemical networks, we do not define the goal state (or end state) explicitly. Instead, it is defined by *S*_*uc*_ implicitly in intervals based on the nature (*fast, slow, reversible and irreversible*) of the reactions in the system, the duration of expansion and the introduction of stochasticity into the system. This should systematically expand the state-space from **X**_*K*_ at *t* to **X**_*K* + 1_ at *t* + 1 by going through each node **n**_*J*_ at depth *ƌ*_*l*_ of the Markov chain graph to evaluate the Markov processes; the expansion aims to occupy most of the probability mass during [**X**_*K*_ + **X**_*K* + 1_], and the Markov processes can be solved for probability distribution at *t* + 1.

Where **X**_*J*_ is the finite set of states and *G*_*mc*_ = (**X**_*J*_, *V*_*μ*_) is the Markov chain graph on **X**_*J*_ associated with *A* = [*a*_*i*,*j*_], given *X*_0_ as the initial state and **X**_*K*_ as the set of the explored state, where *X*_0_ ∈ **X**_*K*_ then the implicit successor is defined as,

44 $$ {S}_{uc}\to {V}_{\mu}\left({\mathbf{X}}_K(t)\right). $$

Equation (44) gives the new states of the system, where, *V*_*μ*_ is the set of stoichiometric vectors *v*_μ_ function defining the state transitions from any present state *X*_*i*_ ∈ **X**_*K*_ to new state ${X}_{i^{\prime }}\notin {\mathbf{X}}_K$. The sample space in the graph contains the unique state of the system stored in a transition matrix, which satisfies Eq. (7) conditions. This transition matrix is a compressed row format (CSR) [49, 50] based on an index of rows ⟶ columns delimited by commas generating the dictionary *Dict* of the model which defines the transitions between nodes in the state-space and the mapping of states. Through *S*_*uc*_, we can know nothing more than the neighbors (child nodes) of the current node (states reachable through a single reaction). We then consider these neighbors (child nodes) as our only goal states; there can be many in numbers. In a situation such as this, search trails are referred to as *blind* or *uninformed searches*. In the following section, we discuss the infrastructure of an *uninformed search*, the type of data structure we will be dealing with.

### Infrastructure for searching

A data structure is required to retain the search track in the graph for *problem state-space* expansion. For each node, *N*_*i*_, of the tree, we create a structure consisting of five elements:

1. *N*_*i*_.State: represents state *X*_*i*_ in the state-space corresponding to *N*_*i*_;
2. *N*_*i*_.Parent: represents the parent node of the child node *N*_*i*_;
3. *N*_*i*_.Depth: represents the depth of state state *X*_*i*_;
4. *N*_*i*_.Cost: represents the cost ${C}_{N_i,{N}_i^{\hbox{'}}}$ of the transition from *N*_*i*_ to ${N}_i^{\prime }$ in the state-space;
5. *N*_*i*_.Action: represents the action applied via *S*_*uc*_ on the parent node to reach *N*_*i*_.

To explore new states in the system, we consider the initial state *state*(*N*_1_) = (*X*_0_, *ƌ*_*l*_) as input to the successor, *S*_*uc*_. Once the expansion is initiated, the *Dict* will temporarily (in run-time) store the information for the transition from one node to another in the state-space that binds to the reaction propensities *a*_*μ*_. This shift is denoted by an arrow →, which shows multiple transitions from the parent nodes to the child nodes containing the end state. The set of nodes ${\mathbf{n}}_J=\left\{{N}_1,{N}_2,\dots ..{N}_{S^{\widetilde{\mathrm{N}}}}\right\}$ is a data structure that incorporates the Markov chain graph *G*_*mc*_. We explore all the nodes that store the set of states **X**_*K*_ as well as some additional information about the state, such as the depth and transition cost, from one state to another in the system. If a set of *states*(**n**_*J*_) = **X**_*J*_, then ${C}_{N_i,{N}_i^{\hbox{'}}}$ is the transition cost to reach $state\left({N}_i^{\prime}\right)={X}_{i^{\prime }}$ from *state*(*N*_1_) = *X*_1_ and *depth*(**n**_*J*_) = ƌ_*l*_ defines the depth of the set of nodes in *G*_*mc*_, then the standard relation between a set of nodes and a set of states is given by **n**_*J*_ = (**X**_*J*_, *ƌ*_*l*_) or  and the standard relation between a single node and a single state is given by *N*_*i*_ = (*X*_*i*_, *ƌ*_*l*_) or  if the transition cost is considered.

**Fig. 16:**
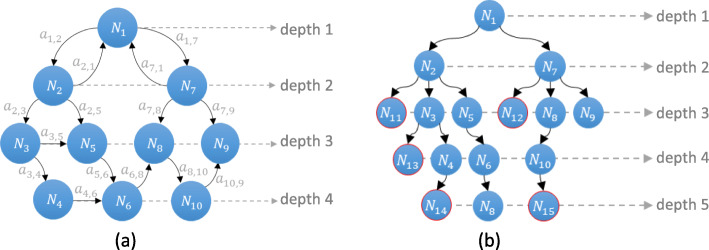
A Markov chain graph and its equivalent tree. (**a**) depicts a Markov chain graph (*G*_*mc*_) with n_*J*_ nodes carrying **X**_*J*_ states. The arcs show transitions between the nodes. Together, they form a Markovian process. (**b**) depicts an equivalent tree Ѭ of *G*_*mc*_ as *DAG* representing the state-space of the system

For example, Fig (a) of Fig. 16 shows the Markov chain graph, *G*_*mc*_, with n_*J*_ = 10, ƌ_*l*_ = 4. Its equivalent tree Ѭ is shown in Fig (b) of Fig. 16 with n_*J*_ = 15, ƌ_*l*_ = 5. In the tree nodes *N*_1_ = *N*_11_ = *N*_12_ carries the same state, *X*_1_ at ƌ_*l*_ = 1, 2 and 3, respectively, where walk *N*_2_ → *N*_11_ and *N*_7_ → *N*_12_ represent the backward reaction of the forward reaction represented by walk *N*_1_ → *N*_2_ and *N*_1_ → *N*_7_, respectively.

The set of nodes with states are represented as
4546

In general, the transition cost, , is defined as:
47

 is the summation of all the propensities *a*_*μ*_ of the *R*_*M*_ reactions that take the system to its final state. For example,  to expand to *state*(*N*_10_) = *X*_10_ of Fig (a) of Fig. 16 is given by


If these are the possible paths for the expansion that expands **X**_*K*_ at every iteration then  will be defined by the only path that has the lowest ${P}^{(t)}\left({\mathbf{X}}_K^{\prime}\right)$. This can be generalized as follows:
48

which means that in order to minimize the expansion cost for the optimal domain **X**_*K*_ at least one path should have states with high probabilities for **X**_*K*_. It is best to follow the path with , which leaks the minimum probabilities of the system.

For large biochemical models, there exist infinite cases when the node is unreachable from the initial or another node; such cases are ignored when  because some probabilities are always dropped in the approximation. Therefore,  as defined by the lowest ${P}^{(t)}\left({\mathbf{X}}_K^{\prime}\right)$ is strictly limited to,
49

**Fig. 17:**
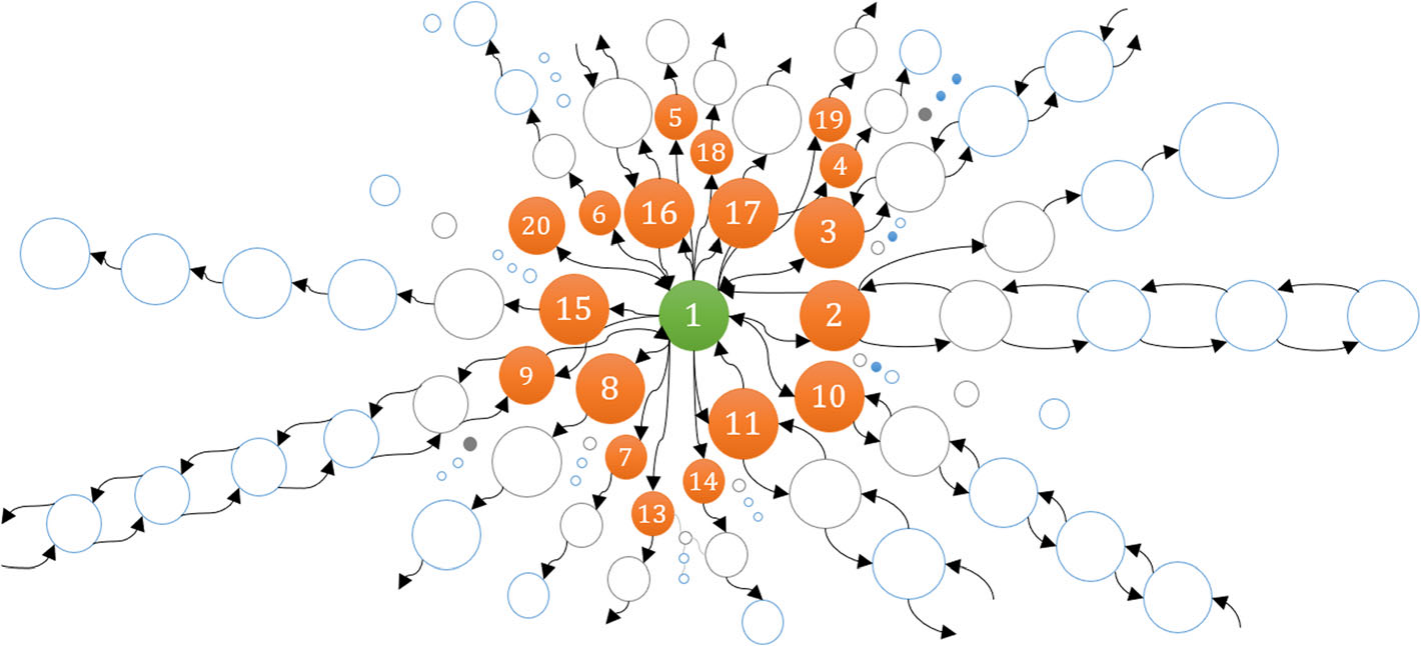
Limits of our visibility in the state-space before expansion. Visualized using a Markov chain graph, where  is the initial node and  are nodes that are directly reachable from the initial node when exactly one ***R***_***M***_ occurs. When a further ***R***_***M***_ occur, the system jumps to other  nodes

Upon expanding the root node *N*_1_, we expand the child nodes carrying new states, and then the child-child nodes are explored. The walk between nodes *N*_*i*_ $\overset{V_{\mu}\left({\mathbf{X}}_K(t)\right)}{\to }$ *N*_*i* + 1_ is defined by dictionary *Dict*. This represents the occurrence of *R*_*M*_ reactions through *M* elementary channels. For Fig (a) of Fig. 16, the typical form of dictionary is given below:

50 $$ D=\left(\left[1\to 2,7\right],\left[2\to 1,3,5\right],\left[3\to 4,5\right],\left[4\to 6\right],\left[5\to 6\right],\left[6\to 8\right],\left[7\to 1,8,9\right],\left[8\to 10\right],\left[9\to Nil\right],\left[10\to 9\right]\right), $$

and is indexed with the propensities, [*a*_*i*,*j*_], for all the *R*_*M*_ reactions. As the propensities are changing by *∆a*_*i*,*j*_, we consider the recent values of *a*_*i*,*j*_ in every iteration of *ISP* that corresponds to the reactions involved. To make the  feasible for any type of biochemical system (*stiff, non-stiff*) to capture probable states, it is important to consider the expansion cost for small *t*_*step*_ (time step). This may be because there are some cases when  to reach two or more different child nodes are equal or very close to each other. In addition, we intend to expand the state-space in the direction of carrying states with high probability mass. To achieve this, we treat or convert our *uninformed search* to an *informed search* infrastructure at run-time to have intuitive knowledge beyond our reach. Figure 17 shows the limits of our visibility in the state-space.

Consequently, it is important to track the reactions which have high propensity function values. As it is difficult to determine the direction of the expansion, in the following section (a), we develop the post successor function on Bayes’ theorem [31, 32] to prioritize the expansion direction based only on those reactions that can be triggered at a particular time point. In sections (b) and (c), we outline the direction strategy with the depth and bounds of the expansion.

#### Bayesian likelihood node projection function

Bayesian methods [32, 51] are based on the principle of linking prior probability with posterior probability through Bayes’ theorem [31, 32]. Posterior probability is the improved form of prior probability, via the likelihood of finding factual support for a valid fundamental hypothesis. Therefore, we employ the standards of Bayes’ theorem to develop a function targeted to ensure the quality of the expansion based on *R*_*M*_ reactions active in the network at any particular moment. For a concise definition for the purpose of fundamentals, refer to SI 6.

To improve the quality of expansion through a projection function, one may find it useful to remove the set of states which have low probabilities before calculating Eq. (3). However, removing these states will compromise accuracy as the step error will increase at every *t*. Moreover, removing these probabilities will greatly affect the solution, as defined at *t*_*f*_ (at which a solution is required), for large dimension systems which have large state-spaces, as the step error will be much higher due to dropping probabilities without solving Eq. (3). In large systems, any species may change its behavior after a certain number of firing of reactions triggering inactive reactions in the network that will affect the probabilities of the states. If a change in behavior increases the probabilities of certain states, then removing them in an earlier stage is not wise.

Through the *Bayesian Likelihood Node Projection* (*BLNP*) function, we seek to predict the posterior probability based on the parent state’s probability and calculate the likelihood of the occurrence of reactions that will take the system from the present state to the future state. Through *BLNP*, we can capture knowledge about the system that will help us to make better predictions about the future state. We are also able to ensure the accuracy of the solution and an optimal domain.

**Fig. 18:**
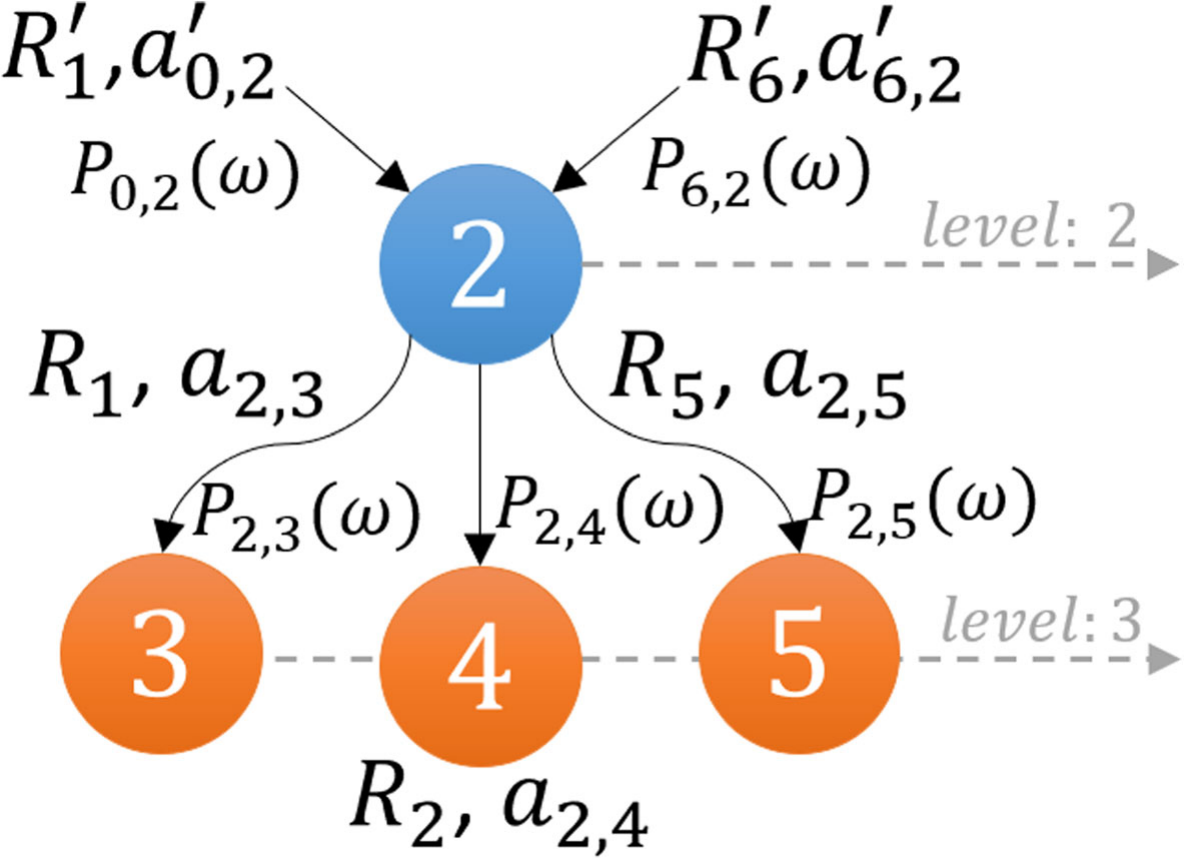
Current *state* (*N*_2_) = *X*_2_, and future *states* (*N*_3, 4, 5_) = (*X*_3, 4, 5_, *d*_*l* = 3_) with corresponding reactions *R*_1_, *R*_2_, *R*_5_ and assumed propensities *a*_2, 3_ = 38, *a*_2, 4_ = 39, *a*_2, 5_ = 40, respectively, at any time *t*, given *b*_0, 2_ = 0.4871, *b*_6, 2_ = 0.5128

**Fig. 19:**
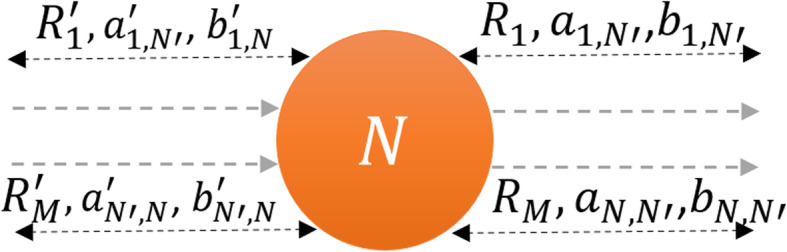
Node *N* as a junction of forward and backward reactions *R*_*M*_, where ${a}_{1,N\prime}^{\prime }$, … .,${a}_{N,N\prime}^{\prime }$ are propensities of the prior reactions. ${b}_{1,N}^{\prime }$, … .,${b}_{N\prime, N}^{\prime }$ are the likelihood of the prior reactions

It is important to decide on the direction of the expansion when choosing the future state of the system, as any reaction can occur and take the system to any new state. To understand this situation more clearly on a node level, we assume a Markov chain graph as shown in Fig. 18 of this system which has almost the same number of species count. In Fig. 19, the expansion is at intermediate position, as the initial state *state*(*N*_0_) = *X*_0_ is already expanded and now the expansion of *state*(*N*_2_) = *X*_2_ can be undertaken. To calculate the likelihood of the occurrence of reactions *R*_1_, *R*_2_, *R*_5_, we consider the propensities *a*_*i*, *j*_ as a parameter. *∆a*_*i*,*j*_ depends on the kinetic parameter of the reaction. To assign weight to our belief, we deduce a function that will calculate the probability of reactions occurring and prioritize the expansion in order from reactions resulting in states with high probabilities to reaction giving states with low probabilities. It is important to note that none of the probabilities will be removed before the calculation of Eq. (5). With this function, the likelihood of occurrence of *R*_*M*_ can be computed.

We consider each node as a junction of the prior reactions $\left\{{R}_1^{\prime}\dots \dots .{R}_M^{\prime}\right\}$ with propensities $\left\{{a}_{1,N}^{\prime}\dots \dots .{a}_{N\prime N}^{\prime}\right\}$ having prior likelihood values $\left\{{b}_{1,N}^{\prime}\dots \dots .{b}_{N^{\prime },N}^{\prime}\right\}$ and future reactions {*R*_1_……. *R*_*M*_} with propensities {*a*_1, *N*′_……. *a*_*N*, *N*′_} having likelihood values {*b*_1, *N*′_……. *b*_*N*, *N*′_}, as given in Fig. 18.

To calculate the likelihood of the reactions, it is necessary to have prior information about the occurrence of reactions. If the expansion is to be done at the initial node *state*(*N*_0_) = *X*_0_ (at level 1), then the prior likelihood value ${b}_{N,N\prime}^{\prime }$ is considered as the initial probability or as ≈1. Once the initial node has been explored, we can calculate the likelihood of the reactions inductively. To calculate the probabilities *b*_1, *N*′_, …. , *b*_*N*, *N*′_ of the occurrence of *R*_1_, …. , *R*_*M*_, we first calculate the weighted probabilities *P*_*N*, 1_(*ω*), … … .,${P}_{N,{N}^{\prime }}\left(\omega \right)$ of a system leaving any state by:
51

and multiply it with the prior probability ${b}_{1,N}^{\prime}\dots \dots .{b}_{N^{\prime },N}^{\prime }$ of the system. This will calculate the likelihood inductively, as *R*_*M*_ is responsible for transforming the system to the present *state*(*N*_*i*_) = *X*_*i*_ at *t*, leading to a function,

52 $$ b\left({N}_{N1,.. NM}|{b}_{1,N\dots .{N}^{\prime },N}^{\prime}\right)=\frac{a_{\mu}\left(X-{v}_{\mu}\right)\ }{\sum \limits_{\mu =1}^M{a}_{\mu}\left(X-{v}_{\mu}\right)}\ast {b}_{N^{\prime },N}^{\prime }{\left(X-{v}_{\mu}\right)}^{\prime } $$

where,

53 $$ {P}_{N,1\dots .N,{N}^{\prime }}\left(\omega \right)=\frac{a_{\mu}\left(X-{v}_{\mu}\right)\ }{\sum \limits_{\mu =1}^M{a}_{\mu}\left(X-{v}_{\mu}\right)}, $$

54 $$ b\left({N}_{N1,.. NM}|{b}_{1,N\dots .{N}^{\prime },N}^{\prime}\right)={P}_{N,1\dots .N,{N}^{\prime }}\left(\omega \right)\ast {b}_{N^{\prime },N}^{\prime }{\left(X-{v}_{\mu}\right)}^{\prime }. $$

Once $b\left({N}_{N1,.. NM}|{b}_{1,N\dots .{N}^{\prime },N}^{\prime}\right)$ is calculated for all the adjacent nodes, the values are arranged in descending order. Every value is bound to one reaction and represents the likelihood of the occurrence of the reaction that takes the system from the present node to the child nodes. Based on likelihood values (highest to lowest), the corresponding reactions are considered one by one and labelled as *true* events for expansion. For example, if a system has *R*_1_, *R*_2_, *R*_3_ reactions that bound to *BLNP* likelihood values in order from highest to lowest, respectively, then three events take the system to new state. When *R*_1_ is considered for expansion, *R*_2_ and *R*_3_ are labeled *false* events and *R*_1_ as the *true* event*.* When the second highest *BLNP* likelihood value is considered, which is for *R*_2_, then it is labeled the *true* event and the others, *R*_1_, *R*_3,_ are labeled false events. Similarly, the last and lowest *BLNP* likelihood value is for *R*_3_, which is labeled as the *true* event and the others as *false* events. All states are added in the domain in order from the 1^st^ *true* event to the 3^rd^ true event. The Eq. (54) of probabilities $b\left({N}_{N1,.. NM}|{b}_{1,N\dots .{N}^{\prime },N}^{\prime}\right)$ is what we call a *BLNP* function.

Figure 18 shows the Markov chain tree for selection present at level 2 (assuming that the initial node is already expanded). Here we calculate the weighted probability of a system leaving *state*(*N*_2_) = *X*_2_ by:

$$ {P}_{2,3}\left(\omega \right)=\frac{a_{\mu}\left(X-{v}_{\mu}\right)\ }{\sum \limits_{\mu =1}^3{a}_{\mu}\left(X-{v}_{\mu}\right)}=0.3247 $$

similarly, *P*_2, 4_(*ω*) = 0.3333 and *P*_2, 5_(*ω*) = 0.3418.

At level 2, the conditional probability of the occurrence of reaction *R*_1_, given the probability of occurrence of reaction *R*_1_ at level 1, is given by:

$$ b\left({N}_{2,3}|{b}_{0,2}^{\prime}\right)=\frac{a_{\mu}\left(X-{v}_{\mu}\right)\ }{\sum \limits_{\mu =1}^3{a}_{\mu}\left(X-{v}_{\mu}\right)}\ast {b}_{0,2}^{\prime}\left(x-{v}_{\mu}\right)^{\prime }, $$

Similarly, the occurrence of reaction *R*_1_ at level 2, given the probability of occurrence of reaction *R*_6_ at level 1, is given by:

$$ b\left({N}_{2,3}|{b}_{6,2}^{\prime}\right)=\frac{a_{\mu}\left(X-{v}_{\mu}\right)\ }{\sum \limits_{\mu =1}^3{a}_{\mu}\left(X-{v}_{\mu}\right)}\ast {b}_{6,2}^{\prime}\left(x-{v}_{\mu}\right)^{\prime }. $$

If at level 1, *state*(*N*_1_) = *X*_1_ and at level 2, *state*(*N*_2_) = *X*_2_ are explored through *R*_1_ then we say that this is a *true* event and temporarily consider other events *false* events with respect to the other reactions. Such a condition holds *true* for the other two cases, when, at level 1, *state*(*N*_1_) = *X*_1_ is explored through *R*_1_ followed by an exploration of *state*(*N*_2_) = *X*_2_ either by *R*_2_ or *R*_5_. Given ${b}_{0,2}^{\prime }{\left(X-{v}_{\mu}\right)}^{\prime }$ and ${b}_{6,2}^{\prime }{\left(X-{v}_{\mu}\right)}^{\prime }$, we calculate the likelihood of all the *R*_*M*_ events, as given in Table 6. The likelihood values of future reactions cannot be equal, as they are based on the probabilities of occurrence of prior reactions.

**Table 6:** Events with the likelihood of future reactions. Here *true* events define the expansion of nodes

${\boldsymbol{b}}_{\boldsymbol{N},{\boldsymbol{N}}^{\prime }}$	***N***_**0**, **2**_	***N***_**6**, **2**_	***b***_***N***, ***N***′_(Value)	***R***_***next***_
$b\left({N}_{2,3}|{b}_{0,2}^{\prime}\right)$	True	False	0.1581	*R*_1, 1_
$b\left({N}_{2,3}|{b}_{6,2}^{\prime}\right)$	False	True	0.1665	*R*_6, 1_
$b\left({N}_{2,4}|{b}_{0,2}^{\prime}\right)$	True	False	0.1623	*R*_1, 2_
$b\left({N}_{2,4}|{b}_{6,2}^{\prime}\right)$	False	True	0.1709	*R*_6, 2_
$b\left({N}_{2,5}|{b}_{0,2}^{\prime}\right)$	True	False	0.1664	*R*_1, 5_
$b\left({N}_{2,5}|{b}_{6,2}^{\prime}\right)$	False	True	0.1752	*R*_6, 5_

From Fig. 18 and Table 6, we can infer, based on the prior reactions for *R*_*M*_, where *M* = {1, 6} that:

**Case 1 (*R***_**1**_**):** At level 2, if the prior reaction is *R*_1_ and holds a *true* event for *N*_0_ → *N*_2_ then:

$$ b\left({N}_{2,5}|{b}_{0,2}^{\prime}\right)>b\left({N}_{2,4}|{b}_{0,2}^{\prime}\right)>b\left({N}_{2,3}|{b}_{0,2}^{\prime}\right) $$

as per *b*_*N*, *N*′_ the likelihood of occurrence of reactions will be in the order *R*_5_ > *R*_2_ > *R*_1_.

**Case 2 (*R***_**6**_**):** At level 2, if the prior reaction is *R*_6_ and holds a *true* event for *N*_6_ → *N*_2_ then

$$ b\left({N}_{2,5}|{b}_{6,2}^{\prime}\right)>b\left({N}_{2,4}|{b}_{6,2}^{\prime}\right)>b\left({N}_{2,3}|{b}_{6,2}^{\prime}\right) $$

as per *b*_*N*, *N*′_ the likelihood of occurrence of reactions will be in the order *R*_5_ > *R*_2_ > *R*_1_.

There will be *M* number of cases (equal to elementary chemical reaction channels) if there are ${R}_M^{\prime }$ prior reactions in the system that bring the system to the current node. The likelihood value will change based on ${b}_{N^{\prime },N}^{\prime}\left(X-{v}_{\mu}\right)^{\prime }$. The *BLNP* function cannot be used standalone for expansion because it only assigns weightage to direction for expansion. In the Intelligent state projection section, we have derived the condition for our expansion strategies to work with the Markov chain graph state-space and defined the criteria for the formation of bounds (domain formed at anytime *t*) with time. The *BLNP* function (with expansion strategies), will choose the probable states in large biochemical systems where it is important to capture the moments at time *t* that define a system’s behavior. *BLNP* will be useful for identifying the most active reactions in the system while guiding the expansion towards the set of states with high probability mass.

#### Latitudinal search strategy

**Fig. 20:**
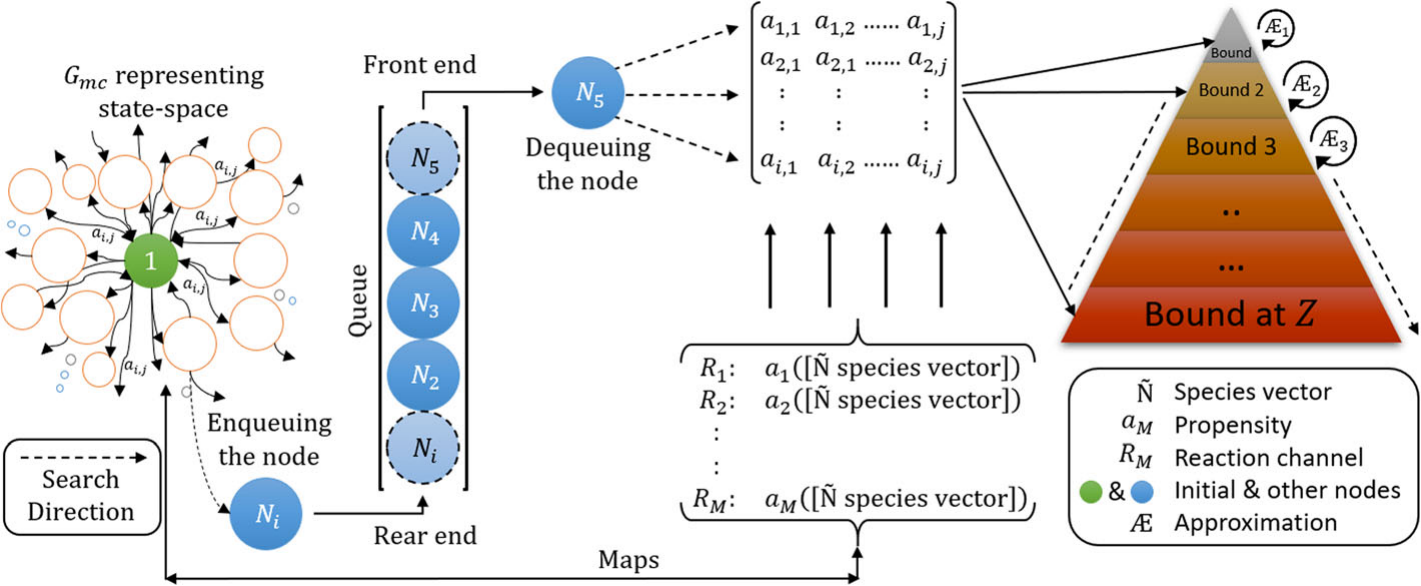
Infrastructure of the *Latitudinal Search* strategy, showing *G*_*mc*_, the *queue* and the domain

We delve deeper into the first subroutine of the *ISP* called the *Intelligent State Projection Latitudinal Search* (*ISP LAS*). Figure 20 manifests the infrastructure of the *LAS* strategy, showing *G*_*mc*_, the *queue* and the domain. *LAS’ queue* data structure is based on the *FIFO* (First In, First Out) method. In this method, the oldest state added to the *queue* is considered first. We define and exploit the direction of expansion step-by-step based on intuitive knowledge (as discussed in section (a)), gained from the probability of future reaction events. We follow the conditions (as discussed in the *results* section). Furthermore, we show step-by-step how the nodes are explored, and states updated in the domain in *I*_*tr*_ iterations.

At level *ƌ*_*l*_ the states are expanded only after all the states at level ƌ_*l*_ − 1 have been expanded; that is, the search is undertaken *level-by-level* and depth ƌ_*l*_ increases in every *I*_*tr*_ iteration. In the case of networks with *reversible* reactions, the *ISP* condition will prevent *LAS* from returning to the state it came from and also prevent transitions containing cycles resulting in *DAG* with no repetition of any state whatsoever; however, changes in propensities *a*_*i*,*j*_ are validated. Verifying the explored states in **X**_*K*_ in iterations ensures that the algorithm completes and that a deadlock in the state transition cycles cannot occur.

The *time complexity* of *LAS* depends on the average transitioning factor Ŧ and depth *ƌ*_*l*_ and is given by (see SI 7 for detailed discussion),
55

where,
56

For the nodes at the deepest level ƌ_*l*_, all walks are valid except for the very last node which stores the end state of the system. Therefore, once the end state is found, based on Eq. (20), *LAS* will zip ${\mathbf{X}}_K^{\prime }$, further leaking the highest probabilities to **X**_*K*_ for the solution of Eq. (3) which includes the end state of the system. As no state is ever repeated in the domain, *space complexity* will decrease when the set of states ${\mathbf{X}}_K^{\prime }$ is bunked at *t*^′^seconds in iterations if Eqs. (19) and (20) are satisfied. In Eq. (13), ${P}^{(t)}\left({\mathbf{X}}_K^{\prime}\right)$ is computed according to Eq. (5) (the exponential form of the CME), where *τ*_*m*_ is the tolerance and *I* is the identity matrix. Due to this stepping bunking of ${\mathbf{X}}_K^{\prime }$ from **X**_*K*_, the time complexity *O*(*Ŧ*^*d* + 1^) reduces to , where |**X**_*J*_| is the size of the state-space [13]. In contrast, the expansion of new nodes carrying similar states tend to increase  however, repetitive states are ignored.

If the input *τ*_*m*_ is too small, the algorithm automatically uses the default value of *sqrt*(*eps*). Here *sqrt* is the square root and *eps* is the default value of the epsilon on machine. The expansion of child nodes containing *state*(*N*_*i*_) = *X*_*i*_ stops if the condition of Eq. (32) is not satisfied. If the criterion of *slow and fast* reaction [12] is considered, then the condition of Eq. (31) or (32) is used, depending on the number of *R*_*M*(*sr*)_ and *R*_*M*(*fs*)_. Table 7 shows the steps of the *LAS* method with the embedded *BLNP* function, from steps 4a to 5b.

**Table 7:** Steps of *ISP* latitudinal search (*LAS*) algorithm

**Step 0:**	**Inputs:** Initial node ***N***_**0**_, ***a***_*μ*_, ***v***_*μ*_, tol ***τ***_***m***_, ***t***_***f***_, ***t***_***step***_ **Initialize:** ${\boldsymbol{Bound}}_{\boldsymbol{lower}}={\boldsymbol{X}}_{\boldsymbol{K}},{\boldsymbol{b}}_{{\boldsymbol{N}}^i,\boldsymbol{N}}^{\hbox{'}}{\left(\boldsymbol{X}-{\boldsymbol{v}}_{\mu}\right)}^{\hbox{'}}={\boldsymbol{P}}^{\left(\boldsymbol{t}\right)}\left({\boldsymbol{X}}_{\mathbf{0}}\right),\boldsymbol{A}=\left[\right]$
**Step 1:**	Start from parent node *N*_*i*_ = (*X*_0_, *ƌ*_*l*_) ← Current State of the system at *t*_*d*_,
**Step 2:**	Flag the current node as explored, update *A* and add the state *X*_*i*_ in the domain so that; *if* 1 − *I*^*T*^ *exp* (*t*. *A*_*j*_). *P*^(*t*)^(*X*_0_) ***≥*** *τ*_*m*_(*leak*) holds true go to *Step 3*; *else* stop the algorithm
**Step 3:**	Sort *exp*(*t*. *A*_*j*_). *P*^(*t*)^(*X*_0_) and shift the set of states in ${\mathbf{X}}_K^{\hbox{'}}\;\mathrm{at}\;{t}^{\hbox{'}}$ having smallest probabilities, if *P*^(*t*)^(**X**_*K*_) ≥ τ_*m*_(*leak*) > ${\mathrm{P}}^{(t)}\left({\mathbf{X}}_K^{\hbox{'}}\right)$ and at *t*_*d*_ update ${\mathbf{X}}_K\leftarrow {\mathbf{X}}_K-{\mathbf{X}}_K^{\hbox{'}}$
**Step 4a:**	Extend the graph dictionary *Dict* by v_*μ*_(*X*_*i*_(*t*)) by 1 level to check all the nodes *n*_*j*_ = (**X**_*j*_, *ƌ*_*l*_ , Ͼ${}_{N_i,{N}_i^{\hbox{'}}}\left.\left(\mathit{\min}\right)\right)$ adjacent to *N*_*i*_: *Bound*_*upper*_ ← *R*_*M*_(*Bound*_*lower*_) reachable by exactly *R*_*M*_ reactions (from fast to slow) having Ͼ${}_{N_i,{N}_i^{\hbox{'}}}\left(\mathit{\min}\right)$. If *n*_*K*_ = (**X**_*K*_, *ƌ*_*l*_, Ͼ${}_{N_i,{N}_i^{\hbox{'}}}\left.\left(\mathit{\min}\right)\right)$ be the set of adjacent nodes such that **n**_*K*_ ∈ **n**_*J*_ then go to next *Step,*
**Step 4b:**	Compute the *BLNP* function for **n**_*K*_∈ *Bound*_*upper*_: $b\left(\left.{N}_{N1,.. NM}\right|{b}_{1,N.\dots {N}^{\hbox{'}}N}^{\hbox{'}}\right)={P}_{N,1.\dots N,N\hbox{'}}\left(\omega \right)\ast {b}_{N^{\hbox{'}},N}^{\hbox{'}}{\left(X-{v}_i\right)}^{\hbox{'}}$
**Step 5a:**	If *n*_*K*_ = (**X**_*K*_, *ƌ*_*l*_, Ͼ${}_{N_i,{N}_i^{\hbox{'}}}\left.\left(\mathit{\min}\right)\right)\in domain$, then *update* the values of the set of states **X**_*K*_ present in *domain* and take *domain* ← *domain*_*previous*_ ∪ *domain* and go back to *Step 1*; *else* If *n*_*K*_ = (**X**_*K*_, *ƌ*_*l*_, Ͼ${}_{N_i,{N}_i^{\hbox{'}}}\left.\left(\mathit{\min}\right)\right)\notin domain$, then add it to the *queue* in order, according to reachability and go to the next *Step,*
**Step 5b:**	*sort* $b\left({N}_{N1,.. NM}\left|{b}_{N1,.. NM}^{\hbox{'}}\right.\right)$ in descending order and update $queue\leftarrow \left( queue;b\left({N}_{N1,.. NM}\left|{b}_{1,N.\dots {N}^{\hbox{'}},N}^{\hbox{'}}\right.\right)\right)$
**Step 6:**	Pull out the nodes *n*_*K*_ = (**X**_*K*_, *ƌ*_*l*_, Ͼ${}_{N_i,{N}_i^{\hbox{'}}}\left.\left(\mathit{\min}\right)\right)$ from the *queue* in order and add the set of states **X**_*K*_ in the domain as *domain* ← *domain* + **X**_*K*_ and take *domain*_*previous*_ ∪ *domain*, then go back to *Step 1,*
	**Output:** *domain* with probable states

*LAS* will be optimal if the transitions between all the states are uniform; that is, all the *R*_*M*_ reactions have the same propensity values. However, in real biochemical models, this condition is unusual. To see a step-by-step demonstration of the *ISP LAS* algorithm on a toy model, refer to SI 2. We now turn our attention to the second variant of the *ISP*. We apply the method to a toy model to see how it differs from *LAS*.

#### Longitudinal-latitudinal search strategy

**Fig. 21:**
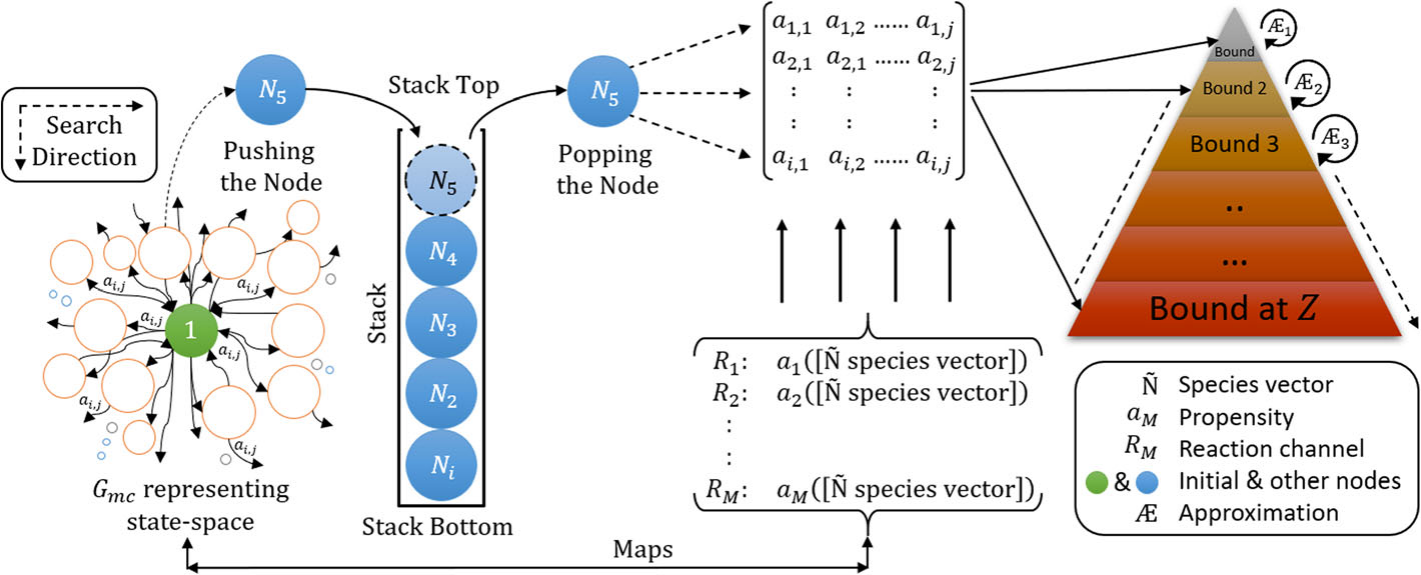
Infrastructure of the *Longitudinal Latitudinal Search* strategy, showing the *G*_*mc*_, the *stack* and the domain

Here, we delve deeper into the second sub-routine of *ISP* called the *Intelligent State Projection Longitudinal Latitudinal Search* (*ISP LOLAS*). Figure 21 visually represents the infrastructure of the *LOLAS* strategy, showing the *G*_*mc*_, *stack* and the domain. The *stack* data structure of *LOLAS* is based on the *LIFO* (Last In, First Out) method. In this method, the newest state added to the *stack* is considered first. In particular, we define the bound limit and exploit the direction of the expansion step-by-step based on intuitive knowledge (as discussed in section (a)), gained from the probability of future reaction events and follow the conditions (as discussed in the *Results* section). Furthermore, we show step-by-step how nodes carrying states are explored in a bidirectional way and how these states were updated in the domain in *I*_*tr*_ iterations.

The states at level ƌ_*l*_ are expanded only after the neighboring states at level ƌ_*l*_ − 1 have been expanded for *R*_*M*_: that is, the search is undertaken *level-by-level.* Depth ƌ_*l*_ increases in the same *I*_*tr*_ iteration up to a certain ƃ_*limit*_ (bound limit). The expansion limit is set by ƃ_*limit*_, ƌ_*step*_ (depth step). In contrast to *LAS*, it is not set by depth ƌ_*l*_,. The *LOLAS* search updates the dictionary *Dict* of *G*_*mc*_ by the stoichiometric vector function, *v*_*μ*_(*X*(*t*)) on state at level ƌ_*l*_ to explore the child nodes carrying states on levels ƌ_*l*_ + 1, ƌ_*l*_ + 2 … .. ƌ_*l*_ + *l*, where *l* = {1, 2…. ∞} and then *retracts* to level ƌ_*l*_ at which new state exploration decisions can be made. In the case of networks with *reversible* reactions, the *ISP* conditions will prevent *LOLAS* from returning to the state it came from and prevent transitions containing cycles resulting in *DAG* with no repetition of any state whatsoever; however, the change in propensities *a*_*i*, *j*_ is validated. Verifying the explored states in **X**_*K*_ in iterations ensures that the algorithm completes and that deadlocks in the state transition cycles cannot occur.

In the absence of ƃ_*limit*_, the algorithm will not retract. It will explore longitudinally by tracking only one *R*_*M*_ reaction. In addition, the algorithm will not terminate with an optimal order domain carrying a maximum probability mass. This would lead to an increase in the approximation error. Instead, it will terminate when carrying only those set of states as a result of tracking only a few *R*_*M*_, creating an insufficient domain for approximation. Therefore, by default, the value of ƃ_*limit*_ ≥ 1 is kept for large systems and can be increased depending upon the model’s dimension and the availability of the testing environment’s random access memory (*RAM*). *LOLAS*’ worst-case *time complexity* depends on the average transitioning factor Ŧ. Depth ƌ_*l*_ is given by (see SI 7 for a detailed discussion):
57

*LOLAS* only stores the transition path to the end state besides the neighbors of each relevant node in the exploration. Once all descendants are updated with the relevant propensities in the projection, it discards the node from the domain (explored), making it ready for the approximation. *LOLAS* first considers the *R*_1_ reaction and the corresponding stoichiometric vector *v*_1_ of the system, to explore all the neighboring states up to bound limit ƃ_*limit*_. It then considers *R*_2_, *R*_3_, … ..,*R*_*M*_ for the same ƃ_*limit*_ and the corresponding *v*_2_, *v*_3_, … .. , *v*_*M*_ to explore the states. For *count*(ƃ_*limit*_), *LOLAS* retracts to the *R*_1_ reaction and explores the new neighboring states longitudinally. It then reconsiders *R*_2_, *R*_3_, … .. , *R*_*M*_ to explore the other states in a similar fashion. Provided with this reaction tracking pattern, the *BLNP* function alters this trend and guides this tracking by considering reactions in a different order based on their propensities and the number of probable states of the system.

If the system is ending in a set of state **X**_*K*_ carried by **n**_*K*_ at *t*_*f*_, then *LOLAS* will explore the states efficiently, as long as *count*(ƃ_*limit*_) ≤ ƃ_*limit*_, otherwise *count*(ƃ_*limit*_) is reset for further expansion. Choosing the appropriate ƌ_*limit*_ and ƃ_*step*_ depends on the type of biochemical reaction network and the computing configuration. Starting with a depth 1 → ƃ_*limit*_, *LOLAS* explores all the states until they return *null*. It then resets the *count*(ƃ_*limit*_) and *retracts* to explore again. In most cases, fewer states are positioned at the lower level. They increase at a higher level when the number of active *R*_*M*_ reactions increases, so retracting provides the ability to track all the reactions simultaneously. The nature of the *LOLAS* expansion means that it is able to find more states at any time *t* compared to *LAS*. It is also able to find them at the deepest level of the graph. The states at depth ƌ_*l*_ are explored once, the states at depth ƌ_*l*_ − 1 are explored twice, states at depth ƌ_*l*_ − 2 are explored three times and so on, until it has explored all the system’s states. If the input *τ*_*m*_ is too small, the algorithm automatically uses the default value of *sqrt*(*eps*). Here *sqrt* is the square root and *eps* is the default value of the epsilon on machine. The expansion of the child nodes containing *state*(*N*_*i*_) = *X*_*i*_ stops if the condition of Eq. (32) is not satisfied. If the *slow and fast* [12] reaction criterion is considered, then either Eq. (31) or (32) conditions are used depending on the number of *R*_*M*(*sr*)_ and *R*_*M*(*fs*)_. Table 8 shows the *LOLAS* method, with an embedded *BLNP* function from steps 4a to 5b.

**Table 8:** Steps of *ISP* longitudinal latitudinal search (*LOLAS*) algorithm

**Step 0:**	**Inputs:** Initial node ***N***_**0**_, ***ƌ***_***step***_, ***ƃ***_***limit***_, ***a***_***μ***_, ***v***_***μ***_, tol ***τ***_***m***_, ***t***_***f***_, ***t***_***step***_ **Initialize:** ${\boldsymbol{Bound}}_{\boldsymbol{lower}}={\boldsymbol{X}}_{\boldsymbol{K}},{\boldsymbol{b}}_{{\boldsymbol{N}}^i,\boldsymbol{N}}^{\hbox{'}}{\left(\boldsymbol{X}-{\boldsymbol{v}}_{\mu}\right)}^{\hbox{'}}={\boldsymbol{P}}^{\left(\boldsymbol{t}\right)}\left({\boldsymbol{X}}_{\mathbf{0}}\right),\boldsymbol{A}=\left[\right]$
**Step 1:**	Initialize *count*(*ƃ*_*limit*_) and start from parent node *N*_*i*_ = (*X*_0_, *ƌ*_*l*_) ← Current state of the system at *t*_*d*_,
**Step 2:**	Flag the current node as explored, update *A* and add the state *X*_*i*_ in the domain so that; *if* 1 − *I*^*T*^ *exp* (*t*. *A*_*j*_). *P*^(*t*)^(*X*_0_) ≥ *τ*_*m*_(*leak*) holds true go to *Step 3*; *else* stop the algorithm.
**Step 3:**	Sort *exp*(*t*. *A*_*j*_). *P*^(*t*)^(*X*_0_) and shift the set of states in ${\mathbf{X}}_K^{\hbox{'}}\;\mathrm{at}\;{t}^{\hbox{'}}$ having smallest probabilities, if *P*^(*t*)^(**X**_*K*_) ≥ τ_*m*_(*leak*) > ${\mathrm{P}}^{(t)}\left({\mathbf{X}}_K^{\hbox{'}}\right)$ and at *t*_*d*_ update ${\mathbf{X}}_K\leftarrow {\mathbf{X}}_K-{\mathbf{X}}_K^{\hbox{'}}$
**Step 4a:**	For *ƌ*_***step***_, extend the graph dictionary *Dict* by v_*μ*_(*X*_*i*_(*t*)) for *count*(***ƃ***_*limit*_) to check all the nodes *n*_*j*_ = (**X**_*j*_, *ƌ*_*l*_ , Ͼ${}_{N_i,{N}_i^{\hbox{'}}}\left.\left(\mathit{\min}\right)\right)$ adjacent to *N*_*i*_: *Bound*_*upper*_ ← *R*_*M*_(*Bound*_*lower*_) reachable by exactly *R*_*M*_ reactions (from fast to slow) having Ͼ${}_{N_i,{N}_i^{\hbox{'}}}\left(\mathit{\min}\right)$. If *n*_*K*_ = (**X**_*K*_, *ƌ*_*l*_ , Ͼ${}_{N_i,{N}_i^{\hbox{'}}}\left.\left(\mathit{\min}\right)\right)$ be the set of adjacent nodes such that **n**_*K*_ ∈ **n**_*J*_, then go to the next *Step,*
**Step 4b:**	Compute the *BLNP* function for **n**_*K*_∈ *Bound*_*upper*_: $b\left(\left.{N}_{N1,.. NM}\right|{b}_{1,N.\dots {N}^{\hbox{'}}N}^{\hbox{'}}\right)={P}_{N,1.\dots N,N\hbox{'}}\left(\omega \right)\ast {b}_{N^{\hbox{'}},N}^{\hbox{'}}{\left(X-{v}_i\right)}^{\hbox{'}}$
**Step 5a:**	If *n*_*K*_ = (**X**_*K*_, *ƌ*_*l*_ , Ͼ${}_{N_i,{N}_i^{\hbox{'}}}\left.\left(\mathit{\min}\right)\right)\in domain$, then *update* the values of the set of states **X**_*K*_ present in *domain* and take *domain* ← *domain*_*previous*_ ∪ *domain* and go back to *Step 1*; *else* If *n*_*K*_ = (**X**_*K*_, *ƌ*_*l*_ , Ͼ${}_{N_i,{N}_i^{\hbox{'}}}\left.\left(\mathit{\min}\right)\right)\notin domain$, then add it to the *stack* in order, according to reachability and go to next *Step,*
**Step 5b:**	*sort* $b\left({N}_{N1,.. NM}\left|{b}_{N1,.. NM}^{\hbox{'}}\right.\right)$ in descending order and update $stack\leftarrow \left( stack;b\left({N}_{N1,.. NM}\left|{b}_{1,N.\dots {N}^{\hbox{'}},N}^{\hbox{'}}\right.\right)\right)$
**Step 6:**	Pop of the top nodes *n*_*K*_ = (**X**_*K*_, *ƌ*_*l*_ , Ͼ${}_{N_i,{N}_i^{\hbox{'}}}\left.\left(\mathit{\min}\right)\right)$ from the *stack* and add the set of states **X**_*K*_ in the domain as *domain* ← *domain* + **X**_*K*_ and take *domain*_*previous*_ ∪ *domain*, and go to next *Step,*
**Step 7:**	If *count*(*ƃ*_*limit*_) = *ƃ*_*limit*_ creates *Bound*_*upper*_ = {*domain*} *up to ƃ*_*limit*_ then label *Bound*_*lower*_ ← *Bound*_*upper*_ and go back to *Step* 1; else *if count*(*ƃ*_*limit*_) < *ƃ*_*limit*_ creates {*domain*} *up to count*(*ƃ*_*limit*_) then go to next *Step*,
**Step 8:**	*count*(***ƃ***_*limit*_)← *count*(***ƃ***_*limit*_) + 1 and go to *Step 4a*
	**Output:** *domain* with probable states

Refer to SI 3 for the step-by-step demonstration of the *ISP LOLAS* algorithm, where we assume the same toy model system.

## Supplementary information

**Additional file 1.**

## Data Availability

All the result data files generated and analyzed during the current study are available in the “isp” repository of https://github.com/rkosarwal/isp. Codes are not publicly available due to search engine privacy but are available from the corresponding or first author on reasonable request.

## Abbreviations

***a***_***i,j***_ or ***a***_***μ***_: Propensity of chemical reaction
***∆a***_***i,j***_: Change in propensity
${\boldsymbol{a}}_{\mathbf{1},\boldsymbol{N}\prime}^{\prime }$, … .,${\boldsymbol{a}}_{\boldsymbol{N},\boldsymbol{N}\prime}^{\prime }$: Propensities of the prior reactions
***a***_***fr***_: Probability of a jump process from state *X*_*i* − 1_ to *X*_*i*_ per unit time
***a***_***rv***_: Probability of a jump process from state *X*_*i*_ to *X*_*i* − 1_ per unit time
***A*** or ***A***_***i,j***_: Defines the transition between *i*, *j* and its weightage
***ƃ***_***limit***_: Exploration bound limit in *LOLAS*
${\boldsymbol{b}}_{{\boldsymbol{N}}^{\prime },\boldsymbol{N}}^{\prime}\left(\boldsymbol{X}-{\boldsymbol{v}}_{\boldsymbol{\mu}}\right)^{\prime }$: Prior Bayesian likelihood values {${b}_{1,N}^{\prime },\dots \dots {b}_{N^{\prime },N}^{\prime }$}
$\boldsymbol{b}\left({\boldsymbol{N}}_{\boldsymbol{N}\mathbf{1},..\boldsymbol{N}\boldsymbol{M}}|{\boldsymbol{b}}_{\mathbf{1},\boldsymbol{N}\dots .{\boldsymbol{N}}^{\prime },\boldsymbol{N}}^{\prime}\right)$: Represents Bayesian likelihood value given prior ${b}_{N^{\prime },N}^{\prime }$
***Bound***_***lower***_ or ***Bound***_***L***_: Define the set of states {*X*_1, 2……*S*_, *b*_1, 2, 3, …. *limit*_} at *ƃ*_*limit*_ already present in the domain for current iteration
***Bound***_***upper***_ or ***Bound***_***U***_: Define the set of states {*X*_1, 2……*S*_, *b*_1, 2, 3, …. *limit*_} at *ƃ*_*limit*_ added in the domain at the end of current iteration
***c***, ***c***_**1**_, ***c***_**2**_: Constants
Total transition/walk cost from node *N*_*i*_ to ${N}_i^{\prime }$
***Dict***: Dictionary of the model having transition records
***ƌ***_***l***_: Exploration depth limit in *LAS*
***ƌ***_***step***_: Exploration depth step in *ISP*
***dim***: Dimension of sub-matrix in Sliding Windows Method
***domain***: Defines the set of states of domain in current iteration that forms *Bound*_*upper*_
***domain***_***previous***_: Defines the set of states of domain in previous iteration that forms *Bound*_*lower*_
***D***_***j***_: Diagonal matrix whose diagonal entries are one
***e***: Markov chain tree edge, representing walk from *N*_*i*_ to ${N}_i^{\prime }$
***e***_**1**_: First unit basis vector in Krylov Subspace Method
***e***_***rror***_: Represents error value in calculation
***exp***(): Exponential function
***eps***: Epsilon
**Έ**_***y***_: Denote the sequence of events Έ_1_, Έ_2_… .,
***f***(***y***): Represents the positive real value function of *y*
***G***_***mc***_: Represents graph associated with the Markov chain tree
${\overline{\boldsymbol{H}}}_{\boldsymbol{dim}}$: Upper matrix (Hessenberg Matrix)
***I***^***T***^: Identity matrix *I* = *diag* (1, 1, …..1)^*T*^
***I***_***tr***_: Denote the iterations in *ISP*
***k***_***M***_: Kinetic parameter of the chemical reaction where *M* = {1, 2…. ∞}
***l***: Used as subscript for length of depth, for example *ƌ*_*l*_
**n**_***J***_: Set of nodes as {${N}_1,{N}_2,\dots \dots .{N}_{S^{\widetilde{\mathrm{N}}}}$}
**n**_***K***_: Set of nodes carrying set of **X**_*K*_ at any iteration
${\mathbf{n}}_{\boldsymbol{K}}^{\prime }$: Set of nodes carrying set of ${\mathbf{X}}_K^{\prime }$ at any iteration
***N***_**0**_: Root node carrying initial state *X*_0_
***N***_***i***_ or ***N***_***i***_: Any node
$\widetilde{\mathbf{N}}$ or $\left\{{\mathbf{S}}_{\mathbf{1}},\dots \dots .{\mathbf{S}}_{\widetilde{\mathbf{N}}}\right\}$: Number of different species
***num***_**1**_, ***num***_**2**_: Random number generated by uniform random number generator (URN)
${\boldsymbol{P}}^{\left({\boldsymbol{t}}_{\mathbf{0}}\right)}\left({\boldsymbol{X}}_{\mathbf{0}}\right)$: Initial probability at *t* = 0
***P***^(***t***)^(**X**_***K***_): Probability of set of states at time *t*
${\boldsymbol{P}}_{\boldsymbol{N},{\boldsymbol{N}}^{\prime }}\left(\boldsymbol{\omega} \right)$: Weighted probability of transition from *N*_*i*_ to ${N}_i^{\prime }$
***R***_***M***_: *M* elementary chemical reaction channels {*R*_1_, *R*_2_……. *R*_*M*_}
${\boldsymbol{R}}_{\boldsymbol{M}}^{\prime }$: Prior *M* elementary chemical reaction channels $\left\{{R}_1^{\prime },{R}_2^{\prime}\dots \dots .{R}_M^{\prime}\right\}$
***R***_***M***(***fs***)_: *M* elementary chemical reaction channels of fast reactions
***R***_***M***(***sr***)_: *M* elementary chemical reaction channels of fast reactions
_***tract***_: Number of retractions in *LOLAS*
${\mathbf{S}}^{\widetilde{\mathbf{N}}}$: Approximate number of states
$\hat{\boldsymbol{S}}$: Number of stages in expansion {1, 2, …….}
SI: Supporting information
***S***_***uc***_: Implicit successor or operator
***sqrt***: Square root
***t***_**0**_: Time at which initial conditions of system are defined
***t***^′^: Time at which ${\mathbf{X}}_K^{\prime }$ is dropped from the domain
***t***: Any random time in seconds
***t***_***d***_: Time at which **X**_*K*_ is updated in the iteration
***t***_***f***_: Final time at which solution is required
**Ŧ**: Transitioning factor
${\boldsymbol{U}}_{{\boldsymbol{X}}_{\boldsymbol{i}},{\boldsymbol{X}}_{{\boldsymbol{i}}^{\prime }}}$: Set of all arborescences
∣***U***∣: Define the cardinality of any set
***v***: Krylov Sub-space method - A column vector of a length equal to the number of different species present in the system
***v***_**μ**_ or ***v***_***M***_: Stoichiometric vector represents the change in the molecular population of the chemical species by the occurrence of one *R*_*M*_ reaction. It also defines the transition from state *X*_*i*_ to ${X}_{i^{\prime }}$ in the Markov chain tree
***v***_***μ***_(***X***(***t***)) or ***v***_***M***_(***X***(***t***)): Stoichiometric vector function, where *X* is any random state
***v***_**μ**_ or ***v***_***M***_: Matrix of all the Stoichiometric vectors [v_1_; v_2_; ……v_μ_]
***W***^***y***^: Probability that is computed inductively by *W*^(0)^ = *P*^(0)^ in uniformization method
${\mathbf{x}}_{\mathbf{1}},\dots \dots .{\mathbf{x}}_{\widetilde{\mathbf{N}}}$: Number of counts of different species
***X*** or ***X***_***i***_ or ${\boldsymbol{X}}_{{\boldsymbol{i}}^{\prime }}$: Any random state
***X***_**0**_: Initial state or initial condition
**X**_***J***_: Ordered set of possible states $\left\{{X}_1,\dots \dots .{X}_{S^{\widetilde{N}}}\right\}$ of the system
**X**_***K***_: Set of new states or domain at any iteration
${\mathbf{X}}_{\boldsymbol{K}}^{\prime }$: Set of states dropped from domain at *t*^′^ at any iteration
***y***, ***y***_**0**_: Positive integers
***Y***_***y***_: Poisson process given that 0 < *y* ≤ *M*
***Z***: Number of bounds in *ISP*
***τ***_***m***_ or ***tol***_***m***_: Tolerance value
***τ***_***m***_(***leak***): ${P}^{(t)}\left({\mathbf{X}}_K^{\prime}\right)$ leakage point
Ӕ: Approximate solution of the CME
***ω***: Weight or cost of single transition from *X*_*i*_ to ${X}_{i^{\prime }}$. It is equivalent to *a*_*i*,*j*_
**Ѫ**_***c***_: Markov chain representing biochemical process
Ѭ: Markov chain tree with **n**_*J*_
***λt***: Uniformization rate
Number of nonzero elements in *P*_*j*_
**φ**: Sample space
***Ω***: Asymptotic lower bound
***O***: Asymptotic upper bound
***Θ***: Asymptotic tight bound
{**1**, **2**……***.K***}: Indexing of set of states and set of nodes
***μ =*** {**1**, **2**, …***.M***}: Channels of chemical reaction propensity
