# Supplementary material for: Novel domain expansion methods to improve the computational efficiency of the Chemical Master Equation solution for large biological networks
Source: BMC Bioinformatics. 2020 Nov 11;21:515. doi: 10.1186/s12859-020-03668-2 (PMC7656229; doi:10.1186/s12859-020-03668-2)
Supplement: Supplementary file 1 — Additional file 1. [file 12859_2020_3668_MOESM1_ESM.docx]

# **SI 1. Implementation Details – Environment, Dependencies**

## SI 1.1. Environment

In this section we discuss about the perquisites, dependencies, and libraries given in following section SI 1.2, must be installed to create the proper environment that enables easier testing and implementation of the presented algorithm(s). The *ssh cmod* with key pair is used with *shell* to connect with the server using Intel®-i3-4005U microprocessor @ 1.70Ghz (2 cores per socket), 4GB DDR3, Ubuntu 16.04.1 desktop 64-bits platform. Files were transferred to AWS® server using *commands,* as well as PuTTy. Before uploading the files using PuTTy, the *ssh* key pair *isp* was generated using PuTTygen (supports RSA, DSA, ECDSA, and Ed25519 keys) to authenticate user through PuTTy and maintain the integrity of the files while transfer. The computing configuration we have used as follows:

| **Configuration** | **Description** |
| --- | --- |
| Amazon® Elastic File System (EFS) | - Configure file system access - Create mount targets - Configure optional settings  1. Performance mode – General Purpose or (2^nd^ option: Max I/O) 2. Throughput mode – Bursting or (2^nd^ option: Provisioned) 3. Enable encryption – Optional (No) |
| Storage | Volume type: EBS (1GB memory with 8GB Elastic Block Storage (EBS) type General Purpose SSD (GP2)). |
| Instance Type | large-m5a (variable ECUs, 16 vCPUs, 2.GHz, AMD EPYC 7571, 64GB memory, EBS only) |
| Ubuntu 16.04.1 | Desktop version running perquisites, dependencies, and essentials |

## SI 1.2. Dependencies and Libraries

| **Name** | **Version / Description** |
| --- | --- |
| Python | v2.7, v3.7 [1,2]  ppa:fkrull/deadsnakes |
| Python Dev package | Contains header files for the python C - API |
| GCC Python | Runs code inside GCC as it compiles, exposing internal data structures as a collection of functions and classes [3] |
| **Dependencies and Perquisites** | |
| Date-util | Powerful extension which provides date, time functions [4] |
| Pyparsing | Used to extract information from textual data (structured) [5] |
| Libpng-dev | Library of function to create and manipulate image format files like PNG [6] |
| Pytz | Library allows cross platform time zone calculations accurately [7] |
| Freetype | Library used to render fonts [8] |
| Cycler | Provides function to create *base* cycler objects, and class takes care of the iteration logic and composition [9] |
| Six | Provides function that smoothens the differences between python versions that is compatible with different versions [10] |
| back ports functools lru cache | Function handles caching mechanism [11] |
| subprocess 32 | Standard library module for python allows you to release new processes [12] |
| Zlib1g-dev | Library that implements the deflate compression method (as found in gzip etc formats) [13] |
| Kiwisolver | An efficient C++ implementation of the Cassowary constraint solving algorithm that ranges 10x to 500x faster than original solver typically gaining 40x improvement. Memory savings are greater than 5x times [14] |
| Intel distribution for python (optional) | package used to boost the computing and operations on elements (optional) [15] |
| **Essentials** | |
| Scipy | V1.1.0 (Builds on Numpy, and used for scientific and technical computing) [16] |
| Numpy | v1.16.3 (It is a numeric python library which provides fast and efficient operations) [17] |
| Setuptools | v1.4.2 (Enhance python standard distribution utilities to support projects packaging) [18] |
| Pip | v19.1.1 (Management system of packages that helps in installation, and management of packages written in python), [for Py2.4, Pip v1.1 is used] [19] |
| Matplotlib | v1.5.1 (Is a library used for plotting whose numerical extension is Numpy) [20] |
| **Libraries** | |
| Libamd | v2.2.0 [21] |
| Libblas3gf | shared library that provides basic linear algebra reference implementations [22] |
| Libc6 | C library that contains standard functions that are required to run on Linux/Ubuntu machines [23] |
| Libgcc1 | a library of internal subroutines [24] |
| Liblapack3gf | library of linear algebra routines 3 [25] |
| Libumfpack | v5.4.0 [26] |
| Libstdc++6 | Runtime library built with GNU compiler to support C++ programs [27] |
| Cmepy | Distribute wheels/eggs that includes py libraries to solve cme [28] |
| gfortan | Invoke Fortran routines like C-code into Python with support for Numeric arrays [29] |
| Libatlas-sse2-dev | Needed for processors that use sse2 instructions [30] |
| Python-all-dev | used as a build dependency for other packages to avoid dependencies which are hardcoded (optional) [31] |
| Pyme | Optimal finite state projection package [32] |
| **Optional** | |
| -U scikit-learn | Analysis, machine learning library [33] |
| Pandas | library written for the programming (in Python) for analysis and data manipulation [34] |
| Numba | v0.39.0 High performance python compiler (dependencies: zlib, sqlite, tcl, tk, openssl, mkl, xz, openmp, icc_rt (vs_2015runtime)) [35] |
| Numexpr | v2.6.5 (dependencies: zlib, sqlite, tcl, tk, openssl, xz, mkl, openmp, icc_rt, numpy) [36] |

**Links to packages:**

1. Python 2.7 https://www.python.org/download/releases/2.7/
2. Python 3.7 https://www.python.org/download/releases/3.7.0/
3. GCC https://gcc-python-plugin.readthedocs.io/en/latest/basics.html
4. Dateutil https://pypi.org/project/python-dateutil/
5. Pyparsing https://pypi.org/project/pyparsing/
6. Libpng-dev http://www.libpng.org/pub/png/libpng.html
7. Pytz https://pypi.org/project/pytz/
8. Freetype https://pypi.org/project/freetype-py/
9. Cycler https://pypi.org/project/Cycler/
10. Six https://pypi.org/project/six/
11. Backports.functools_lru_cache https://pypi.org/project/backports.functools_lru_cache/
12. Subprocess32 https://pypi.org/project/subprocess32/
13. Zlib1g-dev https://packages.ubuntu.com/trusty/zlib1g-dev
14. Kiwisolver https://pypi.org/project/kiwisolver/
15. Intel distribution for python https://software.intel.com/
16. Scipy https://docs.scipy.org/doc/scipy-1.1.0/reference/release.1.1.0.html
17. Numpy https://docs.scipy.org/doc/numpy-1.16.3/user/whatisnumpy.html
18. Setuptools https://pypi.org/project/setuptools/
19. Pip https://pypi.org/project/pip/
20. Matplotlib https://sourceforge.net/projects/matplotlib/files/matplotlib/matplotlib-1.5.1/
21. Libmad https://packages.ubuntu.com/source/trusty/libmad
22. Libblas3gf https://packages.ubuntu.com/search?keywords=libblas3gf
23. Libc6 https://packages.ubuntu.com/xenial/libc6-dev-i386
24. Libgcc1 https://packages.ubuntu.com/xenial/libgcc1
25. Lapack https://launchpad.net/ubuntu/xenial/+source/lapack
26. Libumfpack https://packages.ubuntu.com/search?arch=armhf&keywords=umfpack
27. Libstdc++6 https://packages.ubuntu.com/xenial/libstdc++6
28. Cmepy https://github.com/fcostin/cmepy
29. Gfortran https://packages.ubuntu.com/gfortran
30. Libatlas-sse2-devel https://pkgs.org/download/libatlas-sse2-devel
31. Python-all-dev https://packages.ubuntu.com/trusty/python-all-dev
32. Pyme https://github.com/fcostin/cmepy
33. Scikit-learn http://scikit-learn.org/stable/documentation.html
34. Pandas https://pandas.pydata.org/
35. Numba http://numba.pydata.org/
36. Numexpr https://pypi.org/project/numexpr/2.4.6/
37. Pyssa https://pyssa.readthedocs.io/en/latest/

# **SI 2.** $\boldsymbol{ISPLAS}$ **step-by-step**

To demonstrate the $ISP LAS$ algorithm, let us assume a system with three species, $C$, $A$, $T$, with initial molecular counts of 5, 0, 0, respectively, undergoing reactions as

|  | $\boldsymbol{R}_{\mathbf{1}}\boldsymbol{:}C \underset{\to}{k_{1}} A$, $\boldsymbol{R}_{\mathbf{2}}\boldsymbol{:}A \underset{\to}{k_{2}} T$, $\boldsymbol{R}_{\mathbf{3}}\boldsymbol{:}T \underset{\to}{k_{3}} C$ | (1) |
| --- | --- | --- |

with $k_{1}=k_{2}=$ $k_{3}=$ 1 and depicted as a network in Figure SI 1,


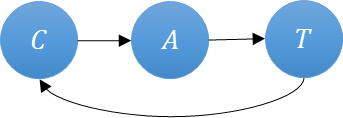


**Figure SI 1. Toy model network.** Showing three $Ñ=3$ species, $C$, $A$, $T$ in a network defining reactions, as given in Eq. (1).

These species counts are used as a state-space to define the toy model and these copy counts are tracked as $\left( \left[ C \right], [A], [T] \right)\inÑ :=(x_{0},x_{1},x_{2})$. In reaction $R_{1}$, the copy counts of $C$ are reduced by 1, which increases the copy count of $A$ by 1. Whereas, reaction $R_{2}$ decreases the count of $A$ by 1 and increases the counts of $T$ by 1. In $R_{3}$, the counts of $T$ are decreased by 1, which increases the counts of $C$ by 1. We can now define the transitions associated with $R_{1},R_{2},R_{3}$ in stoichiometric vector $V_{M}$ matrix as:

|  | $V_{M}=\left[ \begin{matrix} v_{1} \\ v_{2} \\ v_{3} \end{matrix} \right]=\left[ \begin{matrix} -1 \\ 0 \\ 1 \end{matrix} \begin{matrix} 1 \\ -1 \\ 0 \end{matrix} \begin{matrix} 0 \\ 1 \\ 1 \end{matrix} \right].$ | (2) |
| --- | --- | --- |

We express the propensity functions of the three reactions in terms of the states $\left( \left[ C \right], [A], [T] \right)\inÑ$ as:

${\boldsymbol{R}_{\mathbf{1}}\mathbf{:} a}_{1}\left( \left[ S \right],\left[ B \right], \left[ C \right], [P], [E] \right)=k_{1}([S])$,

${\boldsymbol{R}_{\mathbf{2}}\mathbf{:} a}_{2}\left( \left[ S \right],\left[ B \right], \left[ C \right], [P], [E] \right)=k_{2}([B])$,

${\boldsymbol{R}_{\mathbf{3}}\mathbf{:} a}_{3}\left( \left[ S \right],\left[ B \right], \left[ C \right], [P], [E] \right)=k_{3}([B][P])$.

Let, $G_{mc}$ be the discrete-state continuous time Markov chain graph and Ѭ be the equivalent tree of $G_{mc}$ as $DAG$ representing state-space of the system. In growing Markov chain tree, the transition between the nodes:

|  | $N_{i} \underset{\to}{v_{\mu}(X_{0}(t),X_{1}(t),\ldots.,X_{K}(t))} N_{i+1} ,$ | (3) |
| --- | --- | --- |

is defined in the typical form of a dictionary $Dict$. In the following section SI 2.1, we now demonstrate the $LAS$ expansion and update strategy on the toy model.

## SI 2.1. Expansion and Update

Nodes $\mathbf{n}_{J}=\left( \mathbf{X}_{K},ƌ_{l}, Ͼ_{N_{i},N_{i}^{'}}(min) \right)$ carrying states are expanded, as shown by $G_{mc}$ in number of stages ($Ŝ$). From Figs (A) to (F) in Figure SI 2 represent the systematic exploration of the nodes carrying states based on $LAS$ and as depicted up to six stages ($Ŝ=6$), and the walk represents $R_{M}$ reactions with propensities $a_{i,j}$. This shows the change in state from $state\left( N_{i} \right)=X_{i}$ and $state\left( N_{i}^{'} \right)=X_{i^{'}}$ of the system at any time $t$.


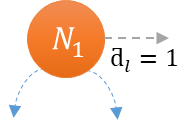

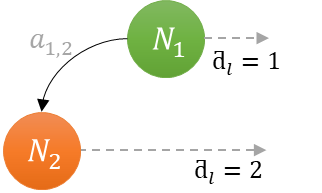

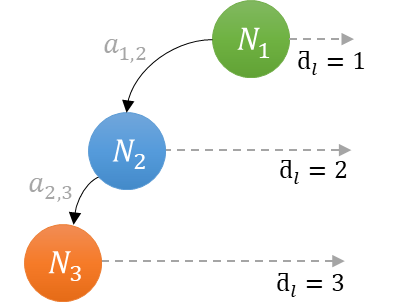


Fig (A). Stage 1, $ƌ_{l}=$ 1 Fig (B). Stage 2, $ƌ_{l}=$ 2 Fig (C). Stage 3, $ƌ_{l}=$ 3


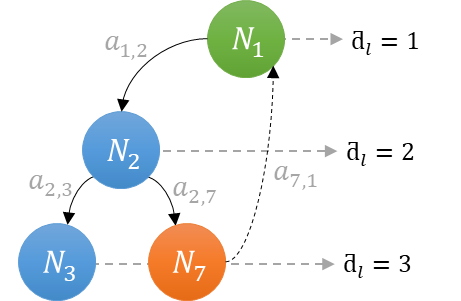

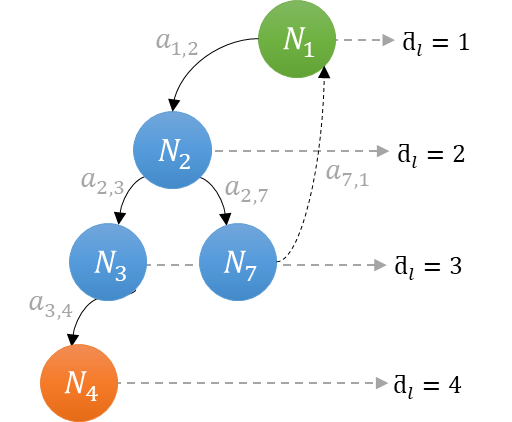


Fig (D). Stage 4, $ƌ_{l}=$ 3 Fig (E). Stage 5, $ƌ_{l}=$ 4


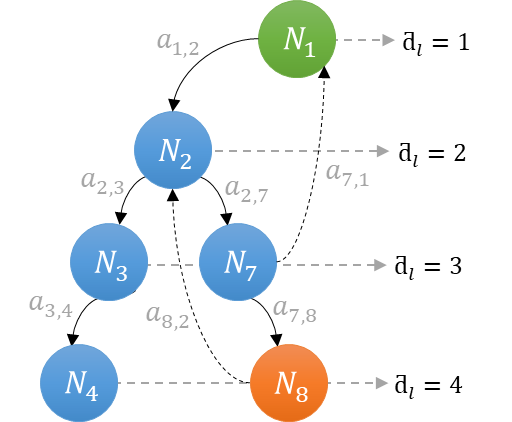


Fig (F). Stage 6, $ƌ_{l}=$ 4

**Figure SI 2.** **Six stages of state exploration based on a** $\boldsymbol{LAS}$ **search for the toy model, given an average transitioning factor of** $\boldsymbol{Ŧ=2}$**.** Fig (A) is the first stage that denote the initial node carrying initial state of the system, Fig (B) is the second stage that denote all the nodes with new node at $\boldsymbol{ƌ}_{\boldsymbol{l}}\boldsymbol{=}$ 2, Fig (C) is the third stage that denote all the nodes with new node at $\boldsymbol{ƌ}_{\boldsymbol{l}}\boldsymbol{=}$ 3, Fig (D) is the fourth stage that denote all the nodes with new node at $\boldsymbol{ƌ}_{\boldsymbol{l}}\boldsymbol{=}$ 3, Fig (E) is the fifth stage that denote all the nodes with new node at $\boldsymbol{ƌ}_{\boldsymbol{l}}\boldsymbol{=}$ 4, Fig (F) is the sixth stage that denote all the nodes with new node at $\boldsymbol{ƌ}_{\boldsymbol{l}}\boldsymbol{=}$ 4.

According to $LAS$ strategy, *step 0* defines the inputs based on the model and some are initialised with the given values. The model is solved for $t_{f}\boldsymbol{=}1.0 sec$ by taking $t_{step}\boldsymbol{=}0.01sec$, $\tau_{m}\boldsymbol{=}1e-6$ with the initial state $state\left( N_{1} \right)=X_{0}$. In *step 1,* $ISP$ starts with the given initial node and this carries the initial state of the system. The initial state is also called the parent state of the system. In *step 2*, the current node is flagged as explored, and the relevant state is added to the domain if Eq. (32) is satisfied otherwise stop the algorithm if it does not.

*Step 3* sorts the probabilities of $exp\left( t_{f}A \right). P^{(t)}\left( X_{j} \right)$ and bunks them from the domain at $t^{'}$ and stores them in the bunker $\mathbf{X}_{K}^{'}$ such that $P^{(t)}\left( \mathbf{X}_{K}^{'} \right)<\tau_{m}(leak)$, resulting in a domain with unique probable states at $t_{d}$. *Step 4a* and *4b*, extend the graph dictionary $Dict$ by a successor function to check all nodes $\mathbf{n}_{J}=\left( \mathbf{X}_{J},ƌ_{l}, Ͼ_{N_{i},N_{i}^{'}}(min) \right)$ that are adjacent to the current node $N_{i}$ and are reachable by exactly $R_{M}$ reactions such that ${Bound}_{upper}\leftarrow R_{M}({Bound}_{lower})$ having $Ͼ_{N_{i},N_{i}^{'}}(min)$. Let $\mathbf{n}_{K}=\left( \mathbf{X}_{K},ƌ_{l}, Ͼ_{N_{i},N_{i}^{'}}(min) \right)$ be the set of adjacent nodes such that $\mathbf{n}_{K}\subset\mathbf{n}_{J}$. Once the adjacent nodes are found the $BLNP$ function calculates $b\left( N_{N1,..NM} | b_{1,N\ldots.N^{'},N}^{'} \right)$ for $\mathbf{n}_{K}\in$ ${Bound}_{upper}$.

*Steps 5a* and *5b* validate if the set of states of set $\mathbf{n}_{K}$ is a part of the domain. Following that, it updates the values of the set of states present in the domain and applies a check to reassure the uniqueness of the states in the domain and resume the algorithm from *step 1*. Otherwise, if the set of states of set $\mathbf{n}_{K}$ is not part of the domain then nodes are added in the $queue$. Further, $queue$ is then updated according to sorted $b\left( N_{N1,..NM} | b_{N1,..NM}^{'} \right)$. *Step 6*, will pull the nodes from the $queue$ in order and add the set of states $\mathbf{X}_{K}$ in the domain followed by a check to reassure the uniquness of the states in the domain. Nodes $\mathbf{n}_{J}=\left( \mathbf{X}_{K},ƌ_{l}, Ͼ_{N_{i},N_{i}^{'}}(min) \right)$ are then expanded, as given in Table SI 1.

**Table SI 1.** $\boldsymbol{LAS}$ **nodes expansion strategy for the toy model.**

| **Iterations** | **Queue** | **Domain** |
| --- | --- | --- |
| 1 | {$N_{1}$} | [Empty] |
| 2 | {$N_{2},N_{7}$} | [$N_{1}$] |
| 3 | {$N_{3},N_{7}$} | [$N_{2},N_{1}]$ |
| 4* | {$N_{7},N_{4},N_{8}$} | [$N_{3}, N_{2},N_{1}]$ |
| 5 | {$N_{4},N_{8}$} | [$N_{7}, N_{3}, N_{2},N_{1}]$ |
| 6* | {$N_{8},N_{5},N_{10}$} | [$N_{4},N_{7}, N_{3}, N_{2},N_{1}]$ |
| 7* | {$N_{5},N_{10},N_{9}$} | [$N_{8},N_{4},N_{7}, N_{3}, N_{2},N_{1}]$ |
| 8* | {$N_{10},N_{9},N_{6},N_{13}$} | [$N_{5},N_{8},N_{4},N_{7}, N_{3}, N_{2},N_{1}]$ |
| 9* | {$N_{9},N_{6},N_{13},N_{11}$} | [$N_{10},N_{5},N_{8},N_{4},N_{7}, N_{3}, N_{2},N_{1}]$ |
| 10 | {$N_{6},N_{13},N_{11}$} | [$N_{9},N_{10},N_{5},N_{8},N_{4},N_{7}, N_{3}, N_{2},N_{1}]$ |
| 11* | {$N_{13},N_{11},N_{17}$} | [$N_{6},N_{9},N_{10},N_{5},N_{8},N_{4},N_{7}, N_{3}, N_{2},N_{1}]$ |
| 12* | {$N_{11},N_{17},N_{14}$} | [$N_{13},N_{6},N_{9},N_{10},N_{5},N_{8},N_{4},N_{7}, N_{3}, N_{2},N_{1}]$ |
| 13* | {$N_{17},N_{14},N_{12}$} | [$N_{11},N_{13},N_{6},N_{9},N_{10},N_{5},N_{8},N_{4},N_{7}, N_{3}, N_{2},N_{1}]$ |
| 14* | {$N_{14},N_{12}$,$N_{18}$} | [$N_{17},N_{11},N_{13},N_{6},N_{9},N_{10},N_{5},N_{8},N_{4},N_{7}, N_{3}, N_{2},N_{1}]$ |
| 15* | {$N_{12}$,$N_{18}$,$N_{15}$} | [$N_{14},N_{17},N_{11},N_{13},N_{6},N_{9},N_{10},N_{5},N_{8},N_{4},N_{7}, N_{3},$  $N_{2},N_{1}]$ |
| 16* | {$N_{18}$,$N_{15}$} | [$N_{12},N_{14},N_{17},N_{11},N_{13},N_{6},N_{9},N_{10},N_{5},N_{8},N_{4},N_{7},$  $N_{3},$ $N_{2},N_{1}]$ |
| 17* | {$N_{15},N_{19}$} | [$N_{18},N_{12},N_{14},N_{17},N_{11},N_{13},N_{6},N_{9},N_{10},N_{5},N_{8},N_{4},$  $N_{7}, N_{3},N_{2},N_{1}]$ |
| 18* | {$N_{19}$,$N_{16}$} | [$N_{15},N_{18},N_{12},N_{14},N_{17},N_{11},N_{13},N_{6},N_{9},N_{10},N_{5},N_{8},$  $N_{4},$ $N_{7}, N_{3},N_{2},N_{1}]$ |
| 19* | {$N_{16}$,$N_{20}$} | [$N_{19},N_{15},N_{18},N_{12},N_{14},N_{17},N_{11},N_{13},N_{6},N_{9},N_{10},N_{5},$  $N_{8},N_{4},N_{7}, N_{3},N_{2},N_{1}]$ |
| 20* | {$N_{20}$} | [$N_{16}$, $N_{19},N_{15},N_{18},N_{12},N_{14},N_{17},N_{11},N_{13},N_{6},N_{9},$  $N_{10},$ $N_{5},N_{8},N_{4},N_{7}, N_{3},N_{2},N_{1}]$ |
| 21* | {$N_{21}$} | [$N_{20}, N_{16}$, $N_{19},N_{15},N_{18},N_{12},N_{14},N_{17},N_{11},N_{13},N_{6},$  $N_{9},$ $N_{10},N_{5},N_{8},N_{4},N_{7}, N_{3},N_{2},N_{1}]$ |
| 22 | {Empty} | [$N_{21},N_{20}, N_{16}$, $N_{19},N_{15},N_{18},N_{12},N_{14},N_{17},$ $N_{11},N_{13},$  $N_{6},$ $N_{9},N_{10},N_{5},N_{8},N_{4},N_{7}, N_{3},N_{2},N_{1}]$ |

To avoid repetition of the states, a check is done at every level as shown by * in Table SI 1. For example, when child node is explored, the state is validated and added to the domain, however if child node is carrying the state which is already in the queue for exploration then the node is not considered. For the toy model, the average number of walk between the nodes is $Ŧ\approx2$.

The states, ${state\left( N_{i} \right)=X}_{i}$ is updated in the domain in every iteration given in Table SI 2 based on the $LAS$ update trend. For example, in *step* 4, nodes $N_{4}$ and $N_{8}$ were added to the *queue* when the state of $N_{3}$ was considered for the domain, but in the next *step* 5 when the state of $N_{7}$ was considered for the domain, no new node was added in the *queue* because node $N_{8}$ was already in the *queue*, this was the neighbour of both $N_{3}$ and $N_{7}$. The corresponding state in node $N_{8}$ will be added to the domain once and will not be repeated in the domain through $N_{7}$ but the changes in propensity ${\Delta a}_{i,j}$ will be validated. When the nodes are pulled from the *queue*, the states are updated according to occurrence of reaction.

**Table SI 2.** $\boldsymbol{LAS}$ **state update strategy for the toy model**.

| **Iterations** | **Depth of exploration** | **Corresponding propensities** $\boldsymbol{\Delta a}_{\boldsymbol{i,j}}$ |
| --- | --- | --- |
| 1 | 1 | Empty |
| 2 | 1 | ${\Delta a}_{1,2}$ |
| 3 | 1 ⟶ 2 | ${\Delta a}_{2,7},{\Delta a}_{2,3},$ $\Delta a_{1,2}$ |
| 4* | 2 ⟶ 3 | ${\Delta a}_{3,8},{\Delta a}_{3,4},{\Delta a}_{2,7},{\Delta a}_{2,3},$ $\Delta a_{1,2}$ |
| 5 | 3 | ${\Delta a}_{7,8},{\Delta a}_{7,1},{\Delta a}_{3,8},{\Delta a}_{3,4},{\Delta a}_{2,7},{\Delta a}_{2,3},$ $\Delta a_{1,2}$ |
| 6* | 3 ⟶ 4 | ${\Delta a}_{4,10},{\Delta a}_{4,5},{\Delta a}_{7,8},{\Delta a}_{7,1},{\Delta a}_{3,8},{\Delta a}_{3,4},{\Delta a}_{2,7},{\Delta a}_{2,3},$ $\Delta a_{1,2}$ |
| 7* | 4 | ${\Delta a}_{8,10},{\Delta a}_{8,9},{\Delta a}_{8,2},{\Delta a}_{4,10},{\Delta a}_{4,5},{\Delta a}_{7,8},{\Delta a}_{7,1},{\Delta a}_{3,8},$  ${\Delta a}_{3,4},{\Delta a}_{2,7},{\Delta a}_{2,3},$ $\Delta a_{1,2}$ |
| 8* | 4 ⟶ 5 | ${\Delta a}_{5,13},{\Delta a}_{5,6},{\Delta a}_{8,10},{\Delta a}_{8,9},{\Delta a}_{8,2},{\Delta a}_{4,10},{\Delta a}_{4,5},{\Delta a}_{7,8},$  ${\Delta a}_{7,1},{\Delta a}_{3,8},{\Delta a}_{3,4},{\Delta a}_{2,7},{\Delta a}_{2,3},$ $\Delta a_{1,2}$ |
| 9* | 5 | ${\Delta a}_{10,13},{\Delta a}_{10,11},{\Delta a}_{10,3},{\Delta a}_{5,13},{\Delta a}_{5,6},{\Delta a}_{8,10},{\Delta a}_{8,9},{\Delta a}_{8,2},$  ${\Delta a}_{4,10},{\Delta a}_{4,5},{\Delta a}_{7,8},{\Delta a}_{7,1},{\Delta a}_{3,8},{\Delta a}_{3,4},{\Delta a}_{2,7},{\Delta a}_{2,3},$ $\Delta a_{1,2}$ |
| 10 | 5 | ${\Delta a}_{9,11},{\Delta a}_{9,7},{\Delta a}_{10,13},{\Delta a}_{10,11},{\Delta a}_{10,3},{\Delta a}_{5,13},{\Delta a}_{5,6},$  ${\Delta a}_{8,10},{\Delta a}_{8,9},{\Delta a}_{8,2},{\Delta a}_{4,10},{\Delta a}_{4,5},{\Delta a}_{7,8},{\Delta a}_{7,1},$  ${\Delta a}_{3,8},{\Delta a}_{3,4},{\Delta a}_{2,7},{\Delta a}_{2,3},$ $\Delta a_{1,2}$ |
| 11* | 5 ⟶ 6 | ${\Delta a}_{6,17},{\Delta a}_{9,11},{\Delta a}_{9,7},{\Delta a}_{10,13},{\Delta a}_{10,11},{\Delta a}_{10,3},{\Delta a}_{5,13},$  ${\Delta a}_{5,6},{\Delta a}_{8,10},{\Delta a}_{8,9},{\Delta a}_{8,2},{\Delta a}_{4,10},{\Delta a}_{4,5},{\Delta a}_{7,8},{\Delta a}_{7,1},$  ${\Delta a}_{3,8},{\Delta a}_{3,4},{\Delta a}_{2,7},{\Delta a}_{2,3},$ $\Delta a_{1,2}$ |
| 12* | 6 | ${\Delta a}_{13,17},{\Delta a}_{13,14},{\Delta a}_{13,4},{\Delta a}_{6,17},{\Delta a}_{9,11},{\Delta a}_{9,7},{\Delta a}_{10,13},$  ${\Delta a}_{10,11},{\Delta a}_{10,3},{\Delta a}_{5,13},{\Delta a}_{5,6},{\Delta a}_{8,10},{\Delta a}_{8,9},{\Delta a}_{8,2},{\Delta a}_{4,10},$  ${\Delta a}_{4,5},{\Delta a}_{7,8},{\Delta a}_{7,1},{\Delta a}_{3,8},{\Delta a}_{3,4},{\Delta a}_{2,7},{\Delta a}_{2,3},$ $\Delta a_{1,2}$ |
| 13* | 6 | ${\Delta a}_{11,14},{\Delta a}_{11,12},{\Delta a}_{11,8},{\Delta a}_{13,17},{\Delta a}_{13,14},{\Delta a}_{13,4},{\Delta a}_{6,17},$  ${\Delta a}_{9,11},{\Delta a}_{9,7},{\Delta a}_{10,13},{\Delta a}_{10,11},{\Delta a}_{10,3},{\Delta a}_{5,13},{\Delta a}_{5,6},$  ${\Delta a}_{8,10},{\Delta a}_{8,9},{\Delta a}_{8,2},{\Delta a}_{4,10},{\Delta a}_{4,5},{\Delta a}_{7,8},{\Delta a}_{7,1},{\Delta a}_{3,8},$  ${\Delta a}_{3,4},{\Delta a}_{2,7},{\Delta a}_{2,3},$ $\Delta a_{1,2}$ |
| 14* | 6 ⟶ 7 | ${\Delta a}_{17,18},{\Delta a}_{17,5},{\Delta a}_{11,14},{\Delta a}_{11,12},{\Delta a}_{11,8},{\Delta a}_{13,17},{\Delta a}_{13,14},$  ${\Delta a}_{13,4},{\Delta a}_{6,17},{\Delta a}_{9,11},{\Delta a}_{9,7},{\Delta a}_{10,13},{\Delta a}_{10,11},{\Delta a}_{10,3},$  ${\Delta a}_{5,13},{\Delta a}_{5,6},{\Delta a}_{8,10},{\Delta a}_{8,9},{\Delta a}_{8,2},{\Delta a}_{4,10},{\Delta a}_{4,5},{\Delta a}_{7,8},$  ${\Delta a}_{7,1},{\Delta a}_{3,8},{\Delta a}_{3,4},{\Delta a}_{2,7},{\Delta a}_{2,3},$ $\Delta a_{1,2}$ |
| 15* | 7 | ${\Delta a}_{14,18},{\Delta a}_{14,15},{\Delta a}_{14,10},{\Delta a}_{17,18},{\Delta a}_{17,5},{\Delta a}_{11,14},{\Delta a}_{11,12},$  ${\Delta a}_{11,8},{\Delta a}_{13,17},{\Delta a}_{13,14},{\Delta a}_{13,4},{\Delta a}_{6,17},{\Delta a}_{9,11},{\Delta a}_{9,7},$  ${\Delta a}_{10,13},{\Delta a}_{10,11},{\Delta a}_{10,3},{\Delta a}_{5,13},{\Delta a}_{5,6},{\Delta a}_{8,10},{\Delta a}_{8,9},$  ${\Delta a}_{8,2},{\Delta a}_{4,10},{\Delta a}_{4,5},{\Delta a}_{7,8},{\Delta a}_{7,1},{\Delta a}_{3,8},{\Delta a}_{3,4},{\Delta a}_{2,7},$  ${\Delta a}_{2,3},$ $\Delta a_{1,2}$ |
| 16* | 7 | ${\Delta a}_{12,15},{\Delta a}_{12,9},{\Delta a}_{14,18},{\Delta a}_{14,15},{\Delta a}_{14,10},{\Delta a}_{17,18},{\Delta a}_{17,5},$  ${\Delta a}_{11,14},{\Delta a}_{11,12},{\Delta a}_{11,8},{\Delta a}_{13,17},{\Delta a}_{13,14},{\Delta a}_{13,4},{\Delta a}_{6,17},$  ${\Delta a}_{9,11},{\Delta a}_{9,7},{\Delta a}_{10,13},{\Delta a}_{10,11},{\Delta a}_{10,3},{\Delta a}_{5,13},{\Delta a}_{5,6},$  ${\Delta a}_{8,10},{\Delta a}_{8,9},{\Delta a}_{8,2},{\Delta a}_{4,10},{\Delta a}_{4,5},{\Delta a}_{7,8},{\Delta a}_{7,1},{\Delta a}_{3,8},$  ${\Delta a}_{3,4},{\Delta a}_{2,7},{\Delta a}_{2,3},$ $\Delta a_{1,2}$ |
| 17* | 7 ⟶ 8 | ${\Delta a}_{18,19},{\Delta a}_{18,13},{\Delta a}_{12,15},{\Delta a}_{12,9},{\Delta a}_{14,18},{\Delta a}_{14,15},{\Delta a}_{14,10},$  ${\Delta a}_{17,18},{\Delta a}_{17,5},{\Delta a}_{11,14},{\Delta a}_{11,12},{\Delta a}_{11,8},{\Delta a}_{13,17},{\Delta a}_{13,14},$  ${\Delta a}_{13,4},{\Delta a}_{6,17},{\Delta a}_{9,11},{\Delta a}_{9,7},{\Delta a}_{10,13},{\Delta a}_{10,11},{\Delta a}_{10,3},$  ${\Delta a}_{5,13},{\Delta a}_{5,6},{\Delta a}_{8,10},{\Delta a}_{8,9},{\Delta a}_{8,2},{\Delta a}_{4,10},{\Delta a}_{4,5},{\Delta a}_{7,8},$  ${\Delta a}_{7,1},{\Delta a}_{3,8},{\Delta a}_{3,4},{\Delta a}_{2,7},{\Delta a}_{2,3},$ $\Delta a_{1,2}$ |
| 18* | 8 | ${\Delta a}_{15,19},{\Delta a}_{15,16},{\Delta a}_{15,11},{\Delta a}_{18,19},{\Delta a}_{18,13},{\Delta a}_{12,15},{\Delta a}_{12,9},$  ${\Delta a}_{14,18},{\Delta a}_{14,15},{\Delta a}_{14,10},{\Delta a}_{17,18},{\Delta a}_{17,5},{\Delta a}_{11,14},{\Delta a}_{11,12},$  ${\Delta a}_{11,8},{\Delta a}_{13,17},{\Delta a}_{13,14},{\Delta a}_{13,4},{\Delta a}_{6,17},{\Delta a}_{9,11},{\Delta a}_{9,7},$  ${\Delta a}_{10,13},{\Delta a}_{10,11},{\Delta a}_{10,3},{\Delta a}_{5,13},{\Delta a}_{5,6},{\Delta a}_{8,10},{\Delta a}_{8,9},$  ${\Delta a}_{8,2},{\Delta a}_{4,10},{\Delta a}_{4,5},{\Delta a}_{7,8},{\Delta a}_{7,1},{\Delta a}_{3,8},{\Delta a}_{3,4},{\Delta a}_{2,7},$  ${\Delta a}_{2,3},$ $\Delta a_{1,2}$ |
| 19* | 8 ⟶ 9 | ${\Delta a}_{19,20},{\Delta a}_{19,14},{\Delta a}_{15,19},{\Delta a}_{15,16},{\Delta a}_{15,11},{\Delta a}_{18,19},$  ${\Delta a}_{18,13},{\Delta a}_{12,15},{\Delta a}_{12,9},{\Delta a}_{14,18},{\Delta a}_{14,15},{\Delta a}_{14,10},{\Delta a}_{17,18},$  ${\Delta a}_{17,5},{\Delta a}_{11,14},{\Delta a}_{11,12},{\Delta a}_{11,8},{\Delta a}_{13,17},{\Delta a}_{13,14},{\Delta a}_{13,4},$  ${\Delta a}_{6,17},{\Delta a}_{9,11},{\Delta a}_{9,7},{\Delta a}_{10,13},{\Delta a}_{10,11},{\Delta a}_{10,3},{\Delta a}_{5,13},$  ${\Delta a}_{5,6},{\Delta a}_{8,10},{\Delta a}_{8,9},{\Delta a}_{8,2},{\Delta a}_{4,10},{\Delta a}_{4,5},{\Delta a}_{7,8},{\Delta a}_{7,1},$  ${\Delta a}_{3,8},{\Delta a}_{3,4},{\Delta a}_{2,7},{\Delta a}_{2,3},$ $\Delta a_{1,2}$ |
| 20* | 9 | ${\Delta a}_{16,20},{\Delta a}_{16,12},{\Delta a}_{19,20},{\Delta a}_{19,14},{\Delta a}_{15,19},{\Delta a}_{15,16},$  ${\Delta a}_{15,11},{\Delta a}_{18,19},$ ${\Delta a}_{18,13},{\Delta a}_{12,15},{\Delta a}_{12,9},{\Delta a}_{14,18},{\Delta a}_{14,15},$  ${\Delta a}_{14,10},{\Delta a}_{17,18},{\Delta a}_{17,5},{\Delta a}_{11,14},{\Delta a}_{11,12},{\Delta a}_{11,8},{\Delta a}_{13,17},$  ${\Delta a}_{13,14},{\Delta a}_{13,4},{\Delta a}_{6,17},{\Delta a}_{9,11},{\Delta a}_{9,7},{\Delta a}_{10,13},{\Delta a}_{10,11},$  ${\Delta a}_{10,3},{\Delta a}_{5,13},{\Delta a}_{5,6},{\Delta a}_{8,10},{\Delta a}_{8,9},{\Delta a}_{8,2},{\Delta a}_{4,10},{\Delta a}_{4,5},$  ${\Delta a}_{7,8},{\Delta a}_{7,1},{\Delta a}_{3,8},{\Delta a}_{3,4},{\Delta a}_{2,7},{\Delta a}_{2,3},$ $\Delta a_{1,2}$ |
| 21* | 9 ⟶ 10 | ${\Delta a}_{20,21},{\Delta a}_{20,15},{\Delta a}_{16,20},{\Delta a}_{16,12},{\Delta a}_{19,20},{\Delta a}_{19,14},$  ${\Delta a}_{15,19},{\Delta a}_{15,16},{\Delta a}_{15,11},{\Delta a}_{18,19},$ ${\Delta a}_{18,13},{\Delta a}_{12,15},{\Delta a}_{12,9},$  ${\Delta a}_{14,18},{\Delta a}_{14,15},{\Delta a}_{14,10},{\Delta a}_{17,18},{\Delta a}_{17,5},{\Delta a}_{11,14},{\Delta a}_{11,12},$  ${\Delta a}_{11,8},{\Delta a}_{13,17},{\Delta a}_{13,14},{\Delta a}_{13,4},{\Delta a}_{6,17},{\Delta a}_{9,11},{\Delta a}_{9,7},$  ${\Delta a}_{10,13},{\Delta a}_{10,11},{\Delta a}_{10,3},{\Delta a}_{5,13},{\Delta a}_{5,6},{\Delta a}_{8,10},{\Delta a}_{8,9},$  ${\Delta a}_{8,2},{\Delta a}_{4,10},{\Delta a}_{4,5},{\Delta a}_{7,8},{\Delta a}_{7,1},{\Delta a}_{3,8},{\Delta a}_{3,4},{\Delta a}_{2,7},$  ${\Delta a}_{2,3},$ $\Delta a_{1,2}$ |
| 22 | 10 ⟶ 11 | ${\Delta a}_{21,16},{\Delta a}_{20,21},{\Delta a}_{20,15},{\Delta a}_{16,20},{\Delta a}_{16,12},{\Delta a}_{19,20},$  ${\Delta a}_{19,14},$ ${\Delta a}_{15,19},{\Delta a}_{15,16},{\Delta a}_{15,11},{\Delta a}_{18,19},$ ${\Delta a}_{18,13},$  ${\Delta a}_{12,15},{\Delta a}_{12,9},{\Delta a}_{14,18},{\Delta a}_{14,15},{\Delta a}_{14,10},{\Delta a}_{17,18},{\Delta a}_{17,5},$  ${\Delta a}_{11,14},{\Delta a}_{11,12},{\Delta a}_{11,8},{\Delta a}_{13,17},{\Delta a}_{13,14},{\Delta a}_{13,4},{\Delta a}_{6,17},$  ${\Delta a}_{9,11},{\Delta a}_{9,7},{\Delta a}_{10,13},{\Delta a}_{10,11},{\Delta a}_{10,3},{\Delta a}_{5,13},{\Delta a}_{5,6},$  ${\Delta a}_{8,10},{\Delta a}_{8,9},{\Delta a}_{8,2},{\Delta a}_{4,10},{\Delta a}_{4,5},{\Delta a}_{7,8},{\Delta a}_{7,1},{\Delta a}_{3,8},$  ${\Delta a}_{3,4},{\Delta a}_{2,7},{\Delta a}_{2,3},$ $\Delta a_{1,2}$ |

The response of$LAS$ for the state-space expansion is shown in Figure SI 3. It clearly shows how the size of the domain (as *2D* *pyramids*) increases with the addition of new probable states. The model is solved for $t_{f}\boldsymbol{=}1.0sec$ by taking $t_{step}\boldsymbol{=}0.01sec$, $\tau_{m}\boldsymbol{=}1e-6$.

The probability bunked during the expansion and approximation of the model is shown in Figure SI 4. There were no reversible reaction in the model and all $R_{M}$ have similar kinetic parameters, so the bunking error became constant from $t\boldsymbol{=}0.05 sec$ after losing $2.21e-02$ of probability in the approximation until $t_{f}$.


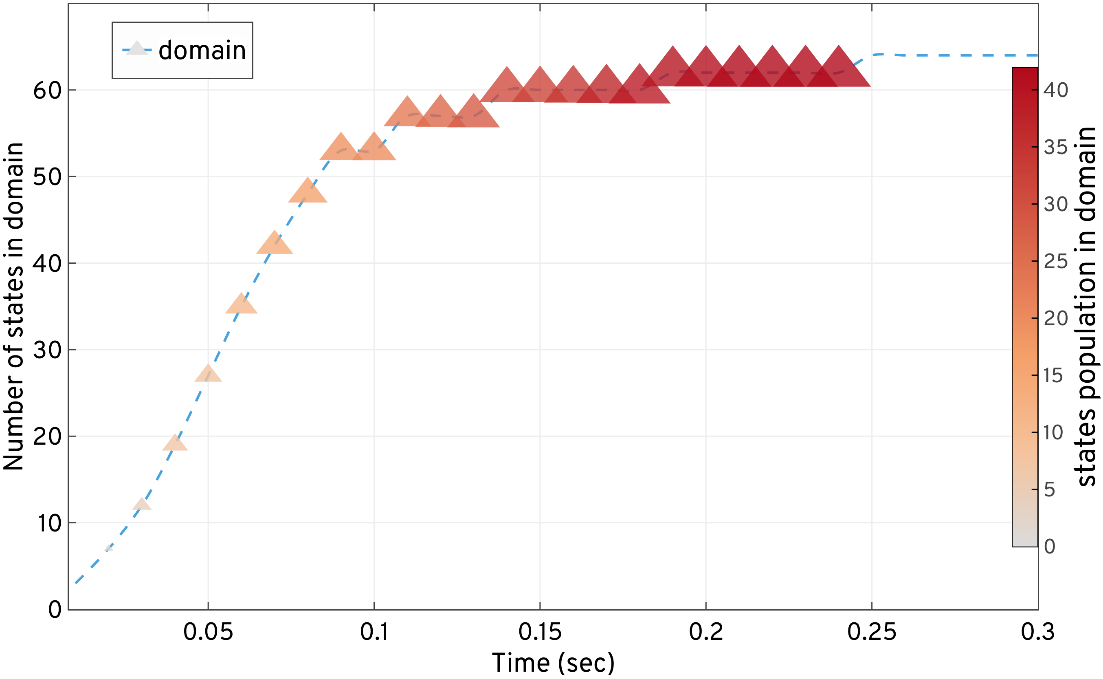


**Figure SI 3. Based on the** $\boldsymbol{LAS}$ **strategy, the response shows how the size of the domain increases with addition of new states with time in the toy model**.


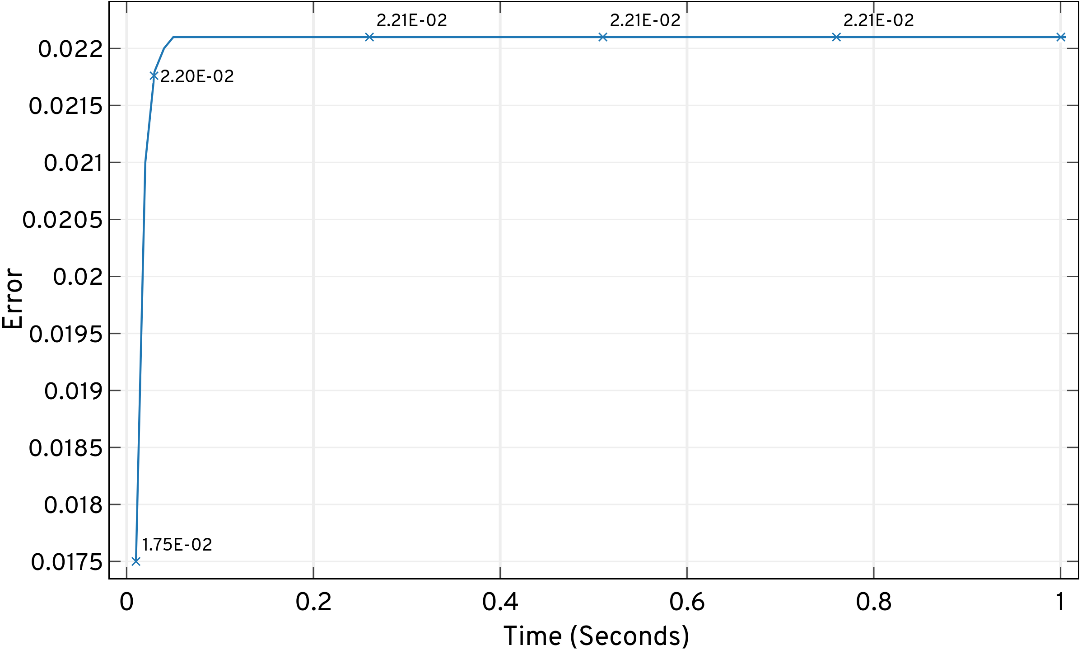


**Figure SI 4.** **Based on the** $\boldsymbol{LAS}$ **strategy, the response shows the total bunked probability during the approximation of the toy model**.

The *time*-*space* complexity of $LAS$ for this model has a linear behaviour as shown in Figure SI 5, for cases like $\boldsymbol{(}\boldsymbol{Ŧ}\boldsymbol{=}2, ƌ_{l}=4)$, and $(Ŧ=5, ƌ_{l}=10)$.

The stochastic behaviour of biochemical systems is such that the state-space depends on the propensity of each $R_{M}$ reaction, and the size of the domain depends upon the number of state explored. $LAS$ successfully creates the domain of an optimum order with $66$ states at $t_{f}$ by introducing new states to the domain with time, as shown in the Figure SI 6.


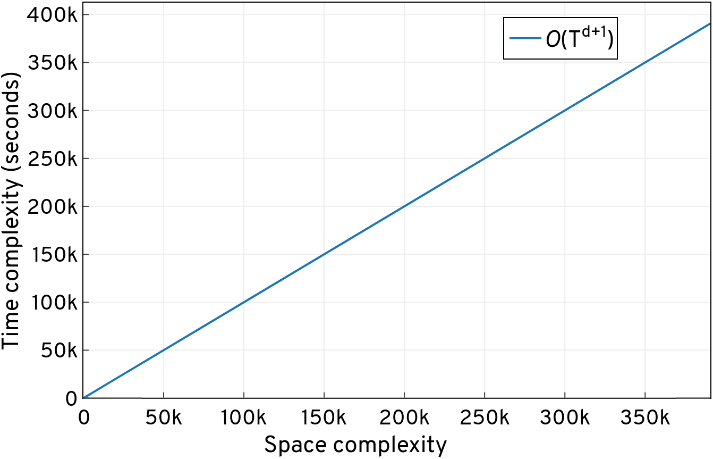


**Figure SI 5.** **Time-Space complexity of the** $\boldsymbol{LAS}$ **response shows the linear behaviour at different average transition factors and the depth of the end state**. For cases like $(Ŧ=2, ƌ_{l}=4)$, and $(Ŧ=5, ƌ_{l}=10)$, $LAS$ has the same response.


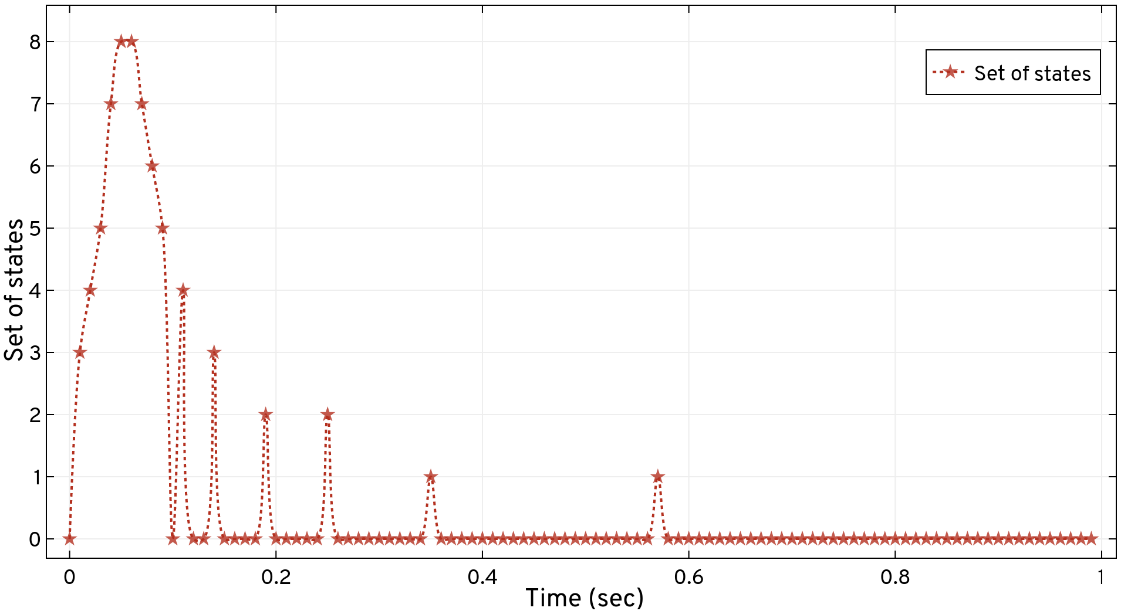


**Figure SI 6. Shows the set of states explored for the toy model after every** $\boldsymbol{LAS}$ **iteration**. Based on the reactions, $LAS$ unfolds the state-space pattern to update states in the domain and expands 66 probable states in 1.0 *sec*.
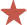
 shows the time point where new set of states is explored and updated in the domain.

The computational expense estimation for exploring this system is given at different time points in Table SI 3. For large biochemical systems, the expense and state-space will vary with the number of states explored. Ideally, if the species present in the model decay with time or because no new states are found in the expansion, then the size of the domain remains the same even if it is solved for a higher $t_{f}$.

**Table SI 3.** $\boldsymbol{LAS}$ **expansion of the state-space and solution of the toy model at different time points**.

| **Time (sec)** | **No. of states explored** | **Exploration performance time (sec)** | **Probability lost** | **Approximation (probability)** |
| --- | --- | --- | --- | --- |
| 0.0 | 0 | 0.0 | 0 | 0 |
| 0.01 | 3 | 0.00332 | 0.0175 | 0.982 |
| 0.05 | 27 | 0.02082 | 0.0221 | 0.977 |
| 0.1 | 53 | 0.03842 | 0.0221 | 0.977 |
| 1.0 | 66 | 0.00910 | 0.0221 | 0.977 |

Similarly, the $ISP LAS$ can be applied on the real biological model to study the expansion of the state-space and its solution.

# **SI 3.** $\boldsymbol{ISPLOLAS}$ **step-by-step**

To demonstrate the $ISP LOLAS$ algorithm, we assume the same toy model system as discussed in SI 2. In section SI 3.1, we demonstrate the $LOLAS$ expansion and update strategy on the toy model.

## SI 3.1. Expansion and Update

Nodes $\mathbf{n}_{J}=\left( \mathbf{X}_{K},ƌ_{l}, Ͼ_{N_{i},N_{i}^{'}}(min) \right)$ carrying states are expanded, as shown by $G_{mc}$ in number of stages ($Ŝ$), assuming $ƌ_{step}=1$, $ƃ_{limit}=2$. From Figs (A) to (F) in Figure SI 7 represent the systematic exploration of the nodes carrying states based on $LOLAS$ and as depicted up to six stages ($Ŝ=6$), and walk representing $R_{M}$ reactions with propensities $a_{i,j}$. This shows the change in state from $state\left( N_{i} \right)=X_{i}$ and $state\left( N_{i}^{'} \right)=X_{i^{'}}$ of the system at time, $t$.

According to $LOLAS$ strategy, for $R_{1}$, explores the states up to $ƌ_{l}=4$ as shown in Fig (D) of Figure SI 7 and retracts to initial node $N_{1}$ to track $R_{2}$. However, $R_{2}$ only occurs when the system is at $N_{2}$. Therefore, the system will consider the expansion from $N_{2}$ for $R_{2}$ and explore until $ƌ_{l}=4$. It retracts continuously for all the $R_{M}$ reactions’ up to $ƃ_{limit}=2$ and once all the states are explored up to $ƌ_{l}=4$ for all the reactions, it reconsider $R_{1}$ and other reactions to further explore and retract.

The toy model is solved for $t_{f}\boldsymbol{=}1.0 sec$ by taking $t_{step}\boldsymbol{=}0.01sec$, $\tau_{m}\boldsymbol{=}1e-6$ with the initial state, $state\left( N_{1} \right)=X_{0}$, $ƃ_{limit}=3$. In *step 1*, the count of $ƃ_{limit}$ is initialised and starts from the given initial state, $state\left( N_{i} \right)=X_{i}$. In *step 2*, matrix $A$ is created by flagging the current node as visited and the state is added in the domain if $e_{rror}\geq\tau_{m}$; otherwise, it will stop the algorithm if it does not. In *step 3*, it *sorts* the probabilities of the expanded states and shifts the set of states in $\mathbf{X}_{K}^{'}$ at $t^{'}$ having smallest probabilities, if $\tau_{m}(leak)>P^{(t)}\left( \mathbf{X}_{K}^{'} \right)$ which leads to an optimal $\mathbf{X}_{K}⟵\mathbf{X}_{K}-\mathbf{X}_{K}^{'}$ set.

In *step 4*, $G_{mc}$ is extended for a count of $ƃ_{limit}$ to check all the nodes $\mathbf{n}_{J}=\left( \mathbf{X}_{J},ƌ_{l}, Ͼ_{N_{i},N_{i}^{'}}(min) \right)$ adjacent to $N_{i}$ reachable exactly by $R_{M}$ reactions having $Ͼ_{N_{i},N_{i}^{'}}(min)$. In *step 5,* the values of the set of states $\mathbf{X}_{K}$ present in the $domain$ is updated if $\mathbf{n}_{J}\in domain$ and unique states was chosen from the $domain$ (current iteration) and ${domain}_{previous}$ (previous iteration); otherwise $\mathbf{n}_{J}$ is added to the $stack$ and moves to the next step. In *step 6*, the nodes are popped out from the $stack$ one-by-one and the corresponding set of states $\mathbf{X}_{K}$ are added to the domain, followed by creating a $domain$ comprising unique states. The nodes $\mathbf{n}_{J}=\left( \mathbf{X}_{K},ƌ_{l}, Ͼ_{N_{i},N_{i}^{'}}(min) \right)$ are then expanded as given in Table SI 4.


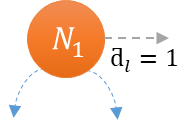

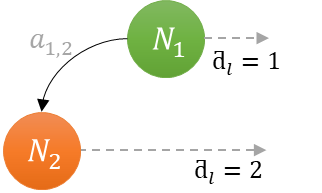

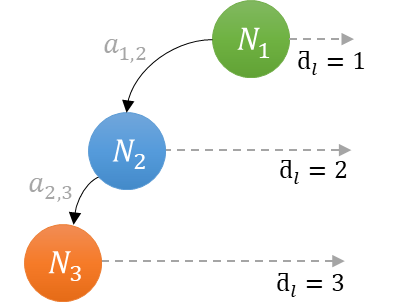


**Fig (A). Stage 1,** $\boldsymbol{ƌ}_{\boldsymbol{l}}\boldsymbol{=}$ **1 Fig (B). Stage 2,** $\boldsymbol{ƌ}_{\boldsymbol{l}}\boldsymbol{=}$ **2 Fig (C). Stage 3,** $\boldsymbol{ƌ}_{\boldsymbol{l}}\boldsymbol{=}$ **3**


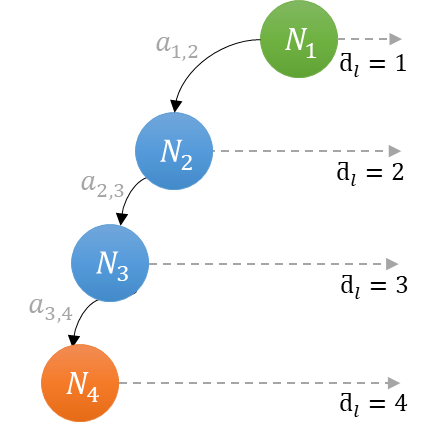

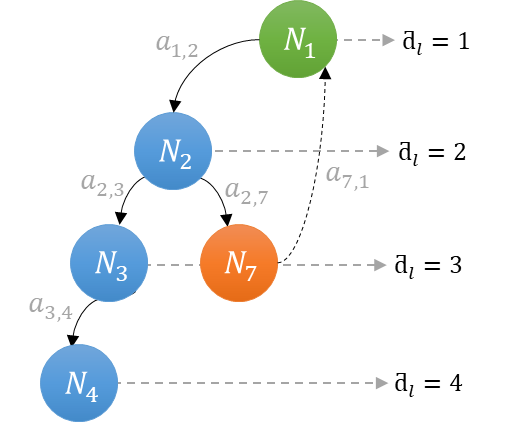


**Fig (D). Stage 4,** $\boldsymbol{ƌ}_{\boldsymbol{l}}\boldsymbol{=}$ **4 Fig (E). Stage 5,** $\boldsymbol{ƌ}_{\boldsymbol{l}}\boldsymbol{=}$ **4**


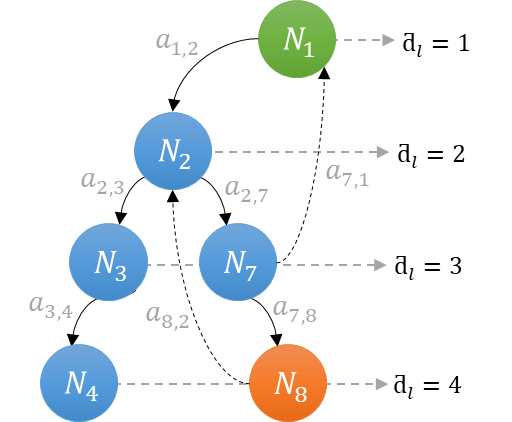


**Fig (F). Stage 6,** $\boldsymbol{ƌ}_{\boldsymbol{l}}\boldsymbol{=}$ **4**

**Figure SI 7. Six stages of state exploration based on** $\boldsymbol{LOLAS}$ **search for the toy model, given average transitioning factor** $\boldsymbol{Ŧ=2}$**, assumed** $\boldsymbol{ƌ}_{\boldsymbol{step}}\boldsymbol{=1}$**,** $\boldsymbol{ƃ}_{\boldsymbol{limit}}\boldsymbol{=3}$. Fig (A) is the first stage that denote the initial node carrying initial state of the system, Fig (B) is the second stage that denote all the nodes with new node at $\boldsymbol{ƌ}_{\boldsymbol{l}}\boldsymbol{=}$ 2, Fig (C) is the third stage that denote all the nodes with new node at $\boldsymbol{ƌ}_{\boldsymbol{l}}\boldsymbol{=}$ 3, Fig (D) is the fourth stage that denote all the nodes with new node at $\boldsymbol{ƌ}_{\boldsymbol{l}}\boldsymbol{=}$ 4, Fig (E) is the fifth stage that denote all the nodes with new node at $\boldsymbol{ƌ}_{\boldsymbol{l}}\boldsymbol{=}$ 4, Fig (F) is the sixth stage that denote all the nodes with new node at $\boldsymbol{ƌ}_{\boldsymbol{l}}\boldsymbol{=}$ 4.

**Table SI 4.** $\boldsymbol{LOLAS}$ **nodes expansion strategy for the toy model**.

| **Iterations** | **Stack** | **Domain** |
| --- | --- | --- |
| 1 | {$N_{1}$} | [Empty] |
| 2 | {$N_{2}$} | [$N_{1}$] |
| 3 | {$N_{3}, N_{7}$} | [$N_{2},N_{1}]$ |
| 4 | {$N_{4}, N_{8}, N_{7}$} | [$N_{3},N_{2},N_{1}]$ |
| 5 | {$N_{5}, N_{10}, N_{8}, N_{7}$} | [$N_{4},N_{3},N_{2},N_{1}]$ |
| 6* | {$N_{9}$} | [$N_{7},N_{8},N_{10},N_{5},N_{4},N_{3},N_{2},N_{1}]$ |
| 7* | {$N_{13}, N_{11}$} | [$N_{9},N_{7},N_{8},N_{10},N_{5},N_{4},N_{3},N_{2},N_{1}]$ |
| 8* | {$N_{17},N_{14}, N_{11}$} | [$N_{13},N_{9},N_{7},N_{8},N_{10},N_{5},N_{4},N_{3},N_{2},N_{1}]$ |
| 9* | {$N_{18},N_{14}, N_{11}$} | [$N_{17},N_{13},N_{9},N_{7},N_{8},N_{10},N_{5},N_{4},N_{3},N_{2},N_{1}]$ |
| 10* | {$N_{19},N_{14}, N_{11}$} | [$N_{18},N_{17},N_{13},N_{9},N_{7},N_{8},N_{10},N_{5},N_{4},N_{3},N_{2},N_{1}]$ |
| 11* | {$N_{20},N_{14}, N_{11}$} | [$N_{19},N_{18},N_{17},N_{13},N_{9},N_{7},N_{8},N_{10},N_{5},N_{4},N_{3},N_{2},N_{1}]$ |
| 12* | {$N_{15}, N_{11}$} | [$N_{14},N_{20},N_{19},N_{18},N_{17},N_{13},N_{9},N_{7},N_{8},N_{10},N_{5},N_{4},N_{3},$  $N_{2},N_{1}]$ |
| 13* | {$N_{16}, N_{11}$} | [$N_{15},N_{14},N_{20},N_{19},N_{18},N_{17},N_{13},N_{9},N_{7},N_{8},N_{10},N_{5},$  $N_{4},N_{3},N_{2},N_{1}]$ |
| 14* | {$N_{11}$} | [$N_{16},N_{15},N_{14},N_{20},N_{19},N_{18},N_{17},N_{13},N_{9},N_{7},N_{8},N_{10},$  $N_{5},N_{4},N_{3},N_{2},N_{1}]$ |
| 15* | {$N_{12}$} | [$N_{11},N_{16},N_{15},N_{14},N_{20},N_{19},N_{18},N_{17},N_{13},N_{9},N_{7},N_{8},$  $N_{10},N_{5},N_{4},N_{3},N_{2},N_{1}]$ |
| 16* | {$N_{21}$} | [$N_{12},N_{11},N_{16},N_{15},N_{14},N_{20},N_{19},N_{18},N_{17},N_{13},N_{9},N_{7},$  $N_{8},$ $N_{10},N_{5},N_{4},N_{3},N_{2},N_{1}]$ |
| 17 | {Empty} | [$N_{21},N_{12},N_{11},N_{16},N_{15},N_{14},N_{20},N_{19},N_{18},N_{17},N_{13},$  $N_{9},N_{7},$ $N_{8},N_{10},N_{5},N_{4},N_{3},N_{2},N_{1}]$ |

To avoid the repetition of states, a check is carried out at every level, as shown by * in Table SI 4. The algorithm rechecks the explored nodes and validates the propensities of the corresponding nodes, $N_{1}$, $N_{2}$, $N_{3}$, $N_{5}$, and then explores the new states through $v_{m}$ to the same bound limit. In general, the algorithm prolongs the retraction until it explores all possible ways to update the states up to a certain $ƃ_{l}$. If $Ꞧ_{tract}$ is the number of retraction, then condition $Ꞧ_{tract}\approx R_{M}$ holds true for $LOLAS$ for any biochemical system having $R_{M}$ reactions. The states, ${state\left( N_{i} \right)=X}_{i}$ are updated in the domain in every iteration as given in Table SI 5, based on the $LOLAS$ update trend.

**Table SI 5.** $\boldsymbol{LOLAS}$ **states update strategy for the toy model**.

| **Iteration** | **Depth of exploration** | **Corresponding propensities** $\boldsymbol{\Delta a}_{\boldsymbol{i,j}}$ |
| --- | --- | --- |
| 1 | 1 | Empty |
| 2 | 1 | ${\Delta a}_{1,2}$ |
| 3 | 1 ⟶ 2 | ${\Delta a}_{2,7},{\Delta a}_{2,3},{\Delta a}_{1,2}$ |
| 4 | 2 ⟶ 3 | ${\Delta a}_{3,8},{\Delta a}_{3,4}, {\Delta a}_{2,7},{\Delta a}_{2,3},{\Delta a}_{1,2}$ |
| 5 | 2 ⟶ 4 | ${\Delta a}_{4,10},{\Delta a}_{4,5}, {\Delta a}_{3,8},{\Delta a}_{3,4}, {\Delta a}_{2,7},{\Delta a}_{2,3},{\Delta a}_{1,2}$ |
| 6* | 4 ⟶ 5,  5 ⟶ 4,  4 ⟶ 3 | ${\Delta a}_{7,8},{\Delta a}_{7,1}, {\Delta a}_{8,9},{\Delta a}_{8,10},{\Delta a}_{8,2}$, ${\Delta a}_{10,11},{\Delta a}_{10,13},$  ${\Delta a}_{10,3},{\Delta a}_{5,13},{\Delta a}_{5,6}$, ${\Delta a}_{4,10},{\Delta a}_{4,5}, {\Delta a}_{3,8},{\Delta a}_{3,4},$  ${\Delta a}_{2,7},{\Delta a}_{2,3},{\Delta a}_{1,2}$ |
| 7* | 3 ⟶ 5 | ${\Delta a}_{9,11},{\Delta a}_{9,7}$,${\Delta a}_{7,8},{\Delta a}_{7,1}, {\Delta a}_{8,9},{\Delta a}_{8,10},{\Delta a}_{8,2}$, ${\Delta a}_{10,11}$, ${\Delta a}_{10,13}, {\Delta a}_{10,3}, {\Delta a}_{5,13},{\Delta a}_{5,6}$, ${\Delta a}_{4,10},{\Delta a}_{4,5},$  ${\Delta a}_{3,8},{\Delta a}_{3,4}, {\Delta a}_{2,7},{\Delta a}_{2,3},{\Delta a}_{1,2}$ |
| 8* | 5 ⟶ 6 | ${\Delta a}_{13,14},{\Delta a}_{13,17},{\Delta a}_{13,4}$,${\Delta a}_{9,11},{\Delta a}_{9,7}$,${\Delta a}_{7,8},{\Delta a}_{7,1},$  ${\Delta a}_{8,9}$, ${\Delta a}_{8,10},{\Delta a}_{8,2}$, ${\Delta a}_{10,11}$,${\Delta a}_{10,13}, {\Delta a}_{10,3}, {\Delta a}_{5,13},$  ${\Delta a}_{5,6}$, ${\Delta a}_{4,10},$ ${\Delta a}_{4,5}, {\Delta a}_{3,8},{\Delta a}_{3,4}, {\Delta a}_{2,7},{\Delta a}_{2,3},{\Delta a}_{1,2}$ |
| 9* | 6 ⟶ 7 | ${\Delta a}_{17,18},{\Delta a}_{17,5}$,${\Delta a}_{13,14},{\Delta a}_{13,17},{\Delta a}_{13,4}$,${\Delta a}_{9,11},{\Delta a}_{9,7}$,${\Delta a}_{7,8},$ ${\Delta a}_{7,1}, {\Delta a}_{8,9}$,${\Delta a}_{8,10},{\Delta a}_{8,2}$, ${\Delta a}_{10,11}$,${\Delta a}_{10,13},$  ${\Delta a}_{10,3}, {\Delta a}_{5,13}$,${\Delta a}_{5,6}$, ${\Delta a}_{4,10},{\Delta a}_{4,5}, {\Delta a}_{3,8},{\Delta a}_{3,4}, {\Delta a}_{2,7},$  ${\Delta a}_{2,3},{\Delta a}_{1,2}$ |
| 10* | 7 ⟶ 8 | ${\Delta a}_{18,19},{\Delta a}_{18,13}$,${\Delta a}_{17,18},{\Delta a}_{17,5}$,${\Delta a}_{13,14},{\Delta a}_{13,17},$  ${\Delta a}_{13,4}$,${\Delta a}_{9,11}$, ${\Delta a}_{9,7}$,${\Delta a}_{7,8}, {\Delta a}_{7,1}, {\Delta a}_{8,9}$,${\Delta a}_{8,10},{\Delta a}_{8,2}$, ${\Delta a}_{10,11}$, ${\Delta a}_{10,13}$, ${\Delta a}_{5,13}$, ${\Delta a}_{5,6}$, ${\Delta a}_{4,10},{\Delta a}_{4,5}, {\Delta a}_{3,8},$  ${\Delta a}_{3,4}, {\Delta a}_{2,7},{\Delta a}_{2,3},{\Delta a}_{1,2}$ |
| 11* | 8 ⟶ 9 | ${\Delta a}_{19,20},{\Delta a}_{19,14}$, ${\Delta a}_{18,19},{\Delta a}_{18,13}$,${\Delta a}_{17,18},{\Delta a}_{17,5}$,  ${\Delta a}_{13,14},{\Delta a}_{13,17},{\Delta a}_{13,4}$, ${\Delta a}_{9,11}$, ${\Delta a}_{9,7}$,${\Delta a}_{7,8}, {\Delta a}_{7,1},$  ${\Delta a}_{8,9}$,${\Delta a}_{8,10},$ ${\Delta a}_{8,2}$, ${\Delta a}_{10,11}$, ${\Delta a}_{10,13}$, ${\Delta a}_{5,13}$, ${\Delta a}_{5,6}$,  ${\Delta a}_{4,10},{\Delta a}_{4,5}, {\Delta a}_{3,8},$ ${\Delta a}_{3,4}, {\Delta a}_{2,7},{\Delta a}_{2,3},{\Delta a}_{1,2}$ |
| 12* | 9 ⟶ 10,  10 ⟶ 7 | ${\Delta a}_{14,15},{\Delta a}_{14,18},{\Delta a}_{14,10}$, ${\Delta a}_{20,21},{\Delta a}_{20,15}$, ${\Delta a}_{19,20},$  ${\Delta a}_{19,14}$, ${\Delta a}_{18,19},{\Delta a}_{18,13}$, ${\Delta a}_{17,18},{\Delta a}_{17,5}$,${\Delta a}_{13,14},$ ${\Delta a}_{13,17},{\Delta a}_{13,4}$, ${\Delta a}_{9,11}$, ${\Delta a}_{9,7}$,${\Delta a}_{7,8},$ ${\Delta a}_{7,1},$ ${\Delta a}_{8,9}$,  ${\Delta a}_{8,10}$, ${\Delta a}_{8,2}$, ${\Delta a}_{10,11}$, ${\Delta a}_{10,13}$, ${\Delta a}_{5,13}$, ${\Delta a}_{5,6}$, ${\Delta a}_{4,10},$  ${\Delta a}_{4,5}, {\Delta a}_{3,8},{\Delta a}_{3,4}, {\Delta a}_{2,7},{\Delta a}_{2,3},{\Delta a}_{1,2}$ |
| 13* | 7 ⟶ 8 | ${\Delta a}_{15,16},{\Delta a}_{15,19},{\Delta a}_{15,11}$, ${\Delta a}_{14,15},{\Delta a}_{14,18},{\Delta a}_{14,10}$, ${\Delta a}_{20,21},$ ${\Delta a}_{20,15}$, ${\Delta a}_{19,20},{\Delta a}_{19,14}$, ${\Delta a}_{18,19},{\Delta a}_{18,13}$, ${\Delta a}_{17,18},{\Delta a}_{17,5}$,${\Delta a}_{13,14},$ ${\Delta a}_{13,17},{\Delta a}_{13,4}$, ${\Delta a}_{9,11}$, ${\Delta a}_{9,7}$,  ${\Delta a}_{7,8},$ ${\Delta a}_{7,1},$ ${\Delta a}_{8,9}$,${\Delta a}_{8,10}$, ${\Delta a}_{8,2}$, ${\Delta a}_{10,11}$, ${\Delta a}_{10,13}$,  ${\Delta a}_{5,13}$, ${\Delta a}_{5,6}$, ${\Delta a}_{4,10},{\Delta a}_{4,5},$ ${\Delta a}_{3,8},{\Delta a}_{3,4}, {\Delta a}_{2,7},{\Delta a}_{2,3},$  ${\Delta a}_{1,2}$ |
| 14* | 8 ⟶ 9 | ${\Delta a}_{16,20},{\Delta a}_{16,12}$, ${\Delta a}_{15,16},{\Delta a}_{15,19},{\Delta a}_{15,11}$, ${\Delta a}_{14,15},$  ${\Delta a}_{14,18},$ ${\Delta a}_{14,10}$, ${\Delta a}_{20,21},$ ${\Delta a}_{20,15}$, ${\Delta a}_{19,20},{\Delta a}_{19,14}$,  ${\Delta a}_{18,19},{\Delta a}_{18,13}$, ${\Delta a}_{17,18},{\Delta a}_{17,5}$, ${\Delta a}_{13,14},$ ${\Delta a}_{13,17},$  ${\Delta a}_{13,4}$, ${\Delta a}_{9,11}$, ${\Delta a}_{9,7}$,${\Delta a}_{7,8},$ ${\Delta a}_{7,1},$ ${\Delta a}_{8,9}$, ${\Delta a}_{8,10}$,  ${\Delta a}_{8,2}$, ${\Delta a}_{10,11}$, ${\Delta a}_{10,13}$, ${\Delta a}_{5,13}$, ${\Delta a}_{5,6}$, ${\Delta a}_{4,10},{\Delta a}_{4,5},$  ${\Delta a}_{3,8},{\Delta a}_{3,4}, {\Delta a}_{2,7},{\Delta a}_{2,3},{\Delta a}_{1,2}$ |
| 15* | 9 ⟶ 6 | ${\Delta a}_{11,12},{\Delta a}_{11,14},{\Delta a}_{11,8}$, ${\Delta a}_{16,20},{\Delta a}_{16,12}$, ${\Delta a}_{15,16},$  ${\Delta a}_{15,19},{\Delta a}_{15,11}$, ${\Delta a}_{14,15},{\Delta a}_{14,18},$ ${\Delta a}_{14,10}$, ${\Delta a}_{20,21},$  ${\Delta a}_{20,15}$, ${\Delta a}_{19,20}, {\Delta a}_{19,14}$, ${\Delta a}_{18,19},{\Delta a}_{18,13}$, ${\Delta a}_{17,18},$  ${\Delta a}_{17,5}$, ${\Delta a}_{13,14},$ ${\Delta a}_{13,17},{\Delta a}_{13,4}$, ${\Delta a}_{9,11}$, ${\Delta a}_{9,7}$, ${\Delta a}_{7,8},$  ${\Delta a}_{7,1},$ ${\Delta a}_{8,9}$, ${\Delta a}_{8,10}$, ${\Delta a}_{8,2}$, ${\Delta a}_{10,11}$, ${\Delta a}_{10,13}$, ${\Delta a}_{5,13}$,  ${\Delta a}_{5,6}$, ${\Delta a}_{4,10},{\Delta a}_{4,5}, {\Delta a}_{3,8},{\Delta a}_{3,4},$ ${\Delta a}_{2,7},{\Delta a}_{2,3},{\Delta a}_{1,2}$ |
| 16* | 6 ⟶ 7 | ${\Delta a}_{12,15},{\Delta a}_{12,9}$, ${\Delta a}_{11,12},{\Delta a}_{11,14},{\Delta a}_{11,8}$, ${\Delta a}_{16,20},$  ${\Delta a}_{16,12}$, ${\Delta a}_{15,16},{\Delta a}_{15,19},$ ${\Delta a}_{15,11}$, ${\Delta a}_{14,15},{\Delta a}_{14,18},$  ${\Delta a}_{14,10}$, ${\Delta a}_{20,21},$ ${\Delta a}_{20,15}$, ${\Delta a}_{19,20},$ ${\Delta a}_{19,14}$, ${\Delta a}_{18,19},$  ${\Delta a}_{18,13}$, ${\Delta a}_{17,18},{\Delta a}_{17,5}$, ${\Delta a}_{13,14},$ ${\Delta a}_{13,17},$ ${\Delta a}_{13,4}$, ${\Delta a}_{9,11}$, ${\Delta a}_{9,7}$, ${\Delta a}_{7,8},$ ${\Delta a}_{7,1},$ ${\Delta a}_{8,9}$, ${\Delta a}_{8,10}$, ${\Delta a}_{8,2}$, ${\Delta a}_{10,11}$, ${\Delta a}_{10,13}$, ${\Delta a}_{5,13}$, ${\Delta a}_{5,6}$, ${\Delta a}_{4,10},{\Delta a}_{4,5},$  ${\Delta a}_{3,8},{\Delta a}_{3,4}, {\Delta a}_{2,7},{\Delta a}_{2,3},{\Delta a}_{1,2}$ |
| 17 | 7 ⟶ 11 | ${\Delta a}_{21,16}$, ${\Delta a}_{12,15},{\Delta a}_{12,9}$, ${\Delta a}_{11,12},{\Delta a}_{11,14},{\Delta a}_{11,8}$, ${\Delta a}_{16,20},$ ${\Delta a}_{16,12}$, ${\Delta a}_{15,16},{\Delta a}_{15,19},$ ${\Delta a}_{15,11}$, ${\Delta a}_{14,15},$  ${\Delta a}_{14,18},$ ${\Delta a}_{14,10}$, ${\Delta a}_{20,21},$ ${\Delta a}_{20,15}$, ${\Delta a}_{19,20},$ ${\Delta a}_{19,14}$, ${\Delta a}_{18,19},{\Delta a}_{18,13}$, ${\Delta a}_{17,18},$ ${\Delta a}_{17,5}$, ${\Delta a}_{13,14},$ ${\Delta a}_{13,17},$ ${\Delta a}_{13,4}$, ${\Delta a}_{9,11}$, ${\Delta a}_{9,7}$, ${\Delta a}_{7,8},$ ${\Delta a}_{7,1}, {\Delta a}_{8,9}$, ${\Delta a}_{8,10}$, ${\Delta a}_{8,2}$, ${\Delta a}_{10,11}$, ${\Delta a}_{10,13}$, ${\Delta a}_{5,13}$, ${\Delta a}_{5,6}$, ${\Delta a}_{4,10},$  ${\Delta a}_{4,5}, {\Delta a}_{3,8},{\Delta a}_{3,4}, {\Delta a}_{2,7},{\Delta a}_{2,3},{\Delta a}_{1,2}$ |

The response of$LOLAS$ for the state-space expansion is shown in Figure SI 8. It clearly shows how the size of the domain (as *2D* *pyramids*) quickly increases with the addition of new probable states as compared to $LAS$.

The probability bunked during the expansion and approximation of model is shown in Figure SI 9. There were no reversible reactions in the model and all $R_{M}$ have similar kinetic parameters, so the error becomes a constant from $t\boldsymbol{=}0.56 sec$ after losing $1.03e-05$ of probability in the approximation up until $t_{f}$.


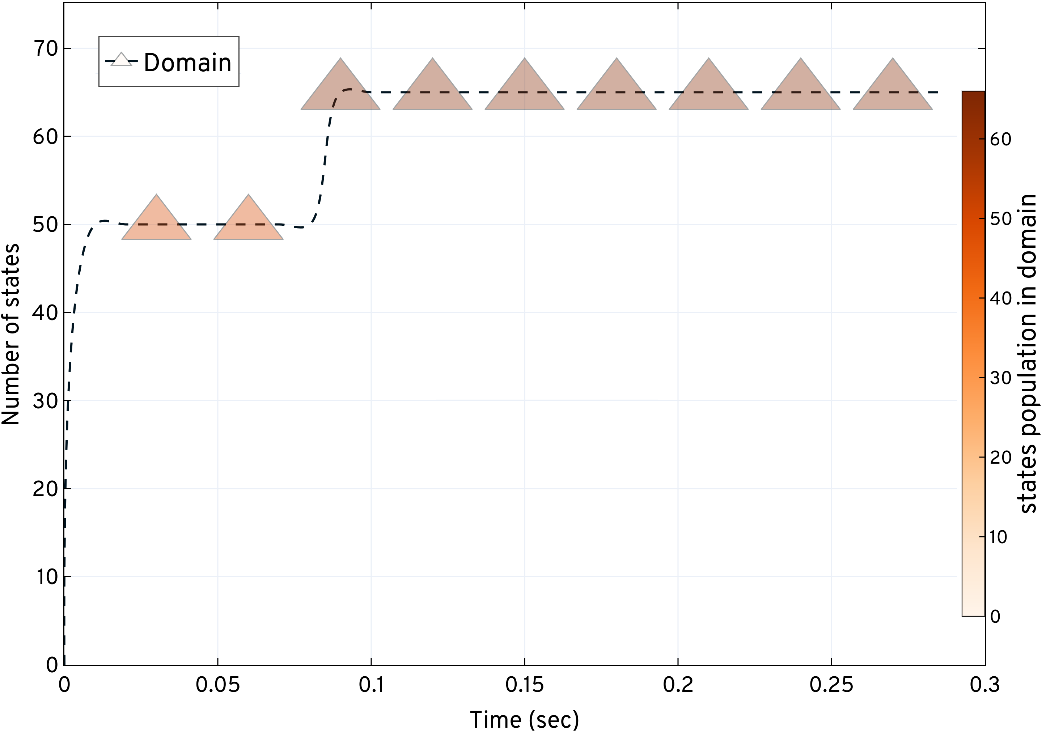


**Figure SI 8. Based on** $\boldsymbol{LOLAS}$ **strategy, the response shows how the size of the domain increases with the addition of new states with time in the toy model**.


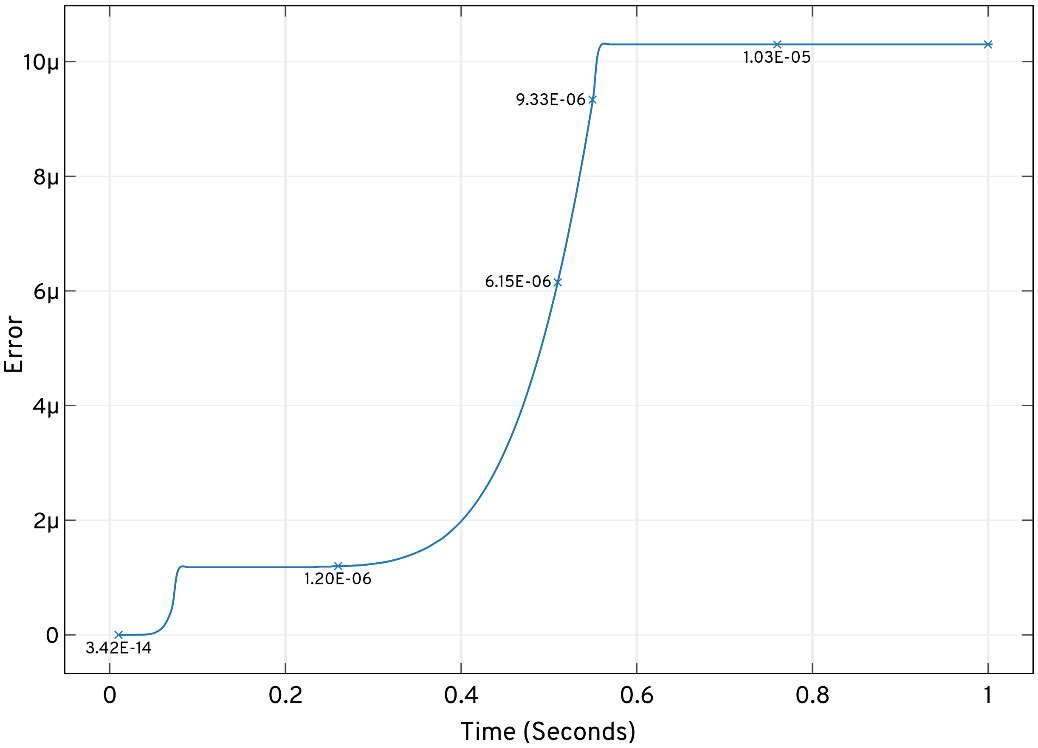


**Figure SI 9. Based on** $\boldsymbol{LOLAS}$ **strategy, the response shows the total bunked probability during the approximation of the toy model**.

$LOLAS$ successfully creates the domain of an optimum order with $66$ states at $t_{f}$ by introducing new states to the domain with time, as shown in Figure SI 10. The response of $LOLAS$ in Figure SI 10 is different from the $LAS$ response in Figure SI 6, which shows that $LOLAS$ is much faster than $LAS$ in finding states. Such behaviour of $LOLAS$ is more suitable for large models having large state-space. In addition, the $ISP$ state-space patterns in Figure SI 6 and Figure SI 10 can be used as a *blueprint* of the model’s state-space to compare it with other model *blueprints* for the characteristics and occurrence of reactions. Such a pattern is considered to predict the behaviour of large network state-space expansion when the set of occurrence of initial reactions are similar in different systems.


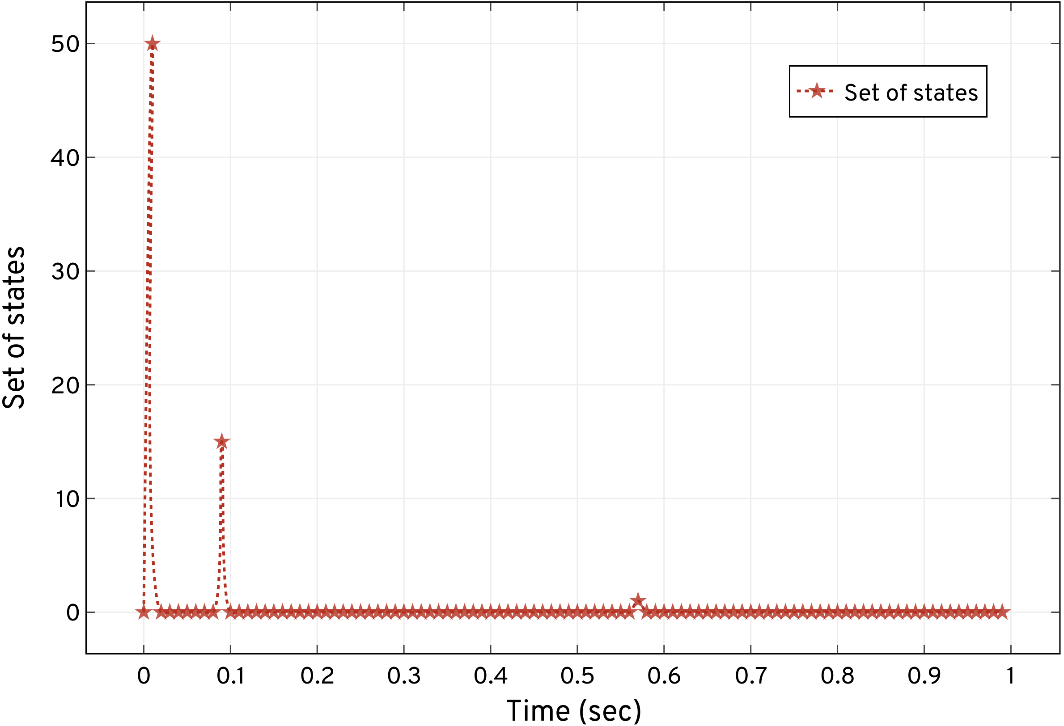


**Figure SI 10. Shows the set of states explored for the toy model after every** $\boldsymbol{LOLAS}$ **iteration.** Based on the reactions, $LOLAS$ unfolds the state-space pattern to update states in the domain and expands 66 probable states in 1.0 *sec*.
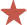
 shows the time point where new set of states is explored and updated in the domain.

The computational expense estimation for exploring this system is given in Table SI 6 for different time points. Overall, the number of states explored by $LAS$ and $LOLAS$; however, the time at which different states are explored changed the bunked probabilities of the states at time points. At $t=$ 0.05 *sec*, $LOLAS$ explores 50 states while $LAS$ explores 27 states taking 0.00882 *sec* and 0.02082 *sec* respectively. This shows that even though the number of states explored by both the variants do not change at $t_{f}$, the number of states present at the time points may change the solution of the CME, as seen for the system like the toy model.

**Table SI 6.**$\boldsymbol{LAS}$ **expansion of the state-space and solution of the toy model at different time points**.

| **Time (sec)** | **No. of states explored** | **Exploration performance time (sec)** | **Probability lost** | **Approximation (probability)** |
| --- | --- | --- | --- | --- |
| 0.0 | 0 | 0.0 | 0 | 0 |
| 0.01 | 50 | 0.04473 | 3.42e-14 | ≈0.99 |
| 0.05 | 50 | 0.00882 | 2.89e-08 | 0.9999999711 |
| 0.1 | 66 | 0.01854 | 1.18e-06 | 0.99999882 |
| 1.0 | 66 | 0.00210 | 1.03e-05 | 0.9999897 |

Similarly, the $ISP LOLAS$ can be applied on real biological model to study the expansion of state-space and its solution.

# **SI 4. G1/S model initial conditions**

## SI 4.1. Initial conditions of the variables

| **Biochemical species** | **Initial condition** | **Model variable** |
| --- | --- | --- |
| $CycD/CDK4/6$ | $2$ | $x_{5}$ |
| $p27/CycD/CDK4/6$ | ${1\times10}^{-3}$ | $x_{11}$ |
| $E2f$ | $0$ | $x_{21}$ |
| $\mathrm{Rb}$ | ${5\times e}^{-2}$ | $x_{23}$ |
| $Rb/E2f$ | $1.95$ | $x_{19}$ |
| $p21/CycD/CDK4/6$ | $0$ | $x_{15}$ |
| $\mathrm{CycD}$ | ${3\times10}^{-2}$ | $x_{0}$ |
| $CDK4/6$ | $5$ | $x_{3}$ |
| $p21$ | $0$ | $x_{14}$ |
| $p27$ | $6.3$ | $x_{10}$ |
| $p16$ | ${1\times10}^{-3}$ | $x_{18}$ |
| $Rb-PP/E2f$ | ${1\times10}^{-3}$ | $x_{20}$ |
| $\mathrm{CycE}$ | ${1\times10}^{-3}$ | $x_{1}$ |
| $CycE/CDK2-P$ | ${1\times10}^{-3}$ | $x_{7}$ |
| $CycA/CDK2-P$ | ${1\times10}^{-4}$ | $x_{9}$ |
| $'X'$ | ${1\times10}^{-4}$ | $x_{26}$ |
| $Rb-PPPP$ | ${1\times10}^{-2}$ | $x_{22}$ |
| $p21/CycE/CDK2-P$ | $0$ | $x_{16}$ |
| $p21/CycA/CDK2-P$ | $0$ | $x_{17}$ |
| $p27/CycE/CDK2-P$ | $1$ | $x_{12}$ |
| $p27/CycA/CDK2-P$ | ${1\times10}^{-4}$ | $x_{13}$ |
| $CDK2$ | $13.5$ | $x_{4}$ |
| $CycE/CDK2$ | ${1\times10}^{-3}$ | $x_{6}$ |
| $p53$ | ${2.65\times10}^{-2}$ | $x_{24}$ |
| $CycA/CDK2$ | ${4\times10}^{-4}$ | $x_{8}$ |

## SI 4.2. Kinetic parameter values for reactions involved in the G1/S model

| **Parameter** | **Value** | **Parameter** | **Value** | **Parameter** | **Value** |
| --- | --- | --- | --- | --- | --- |
| $\boldsymbol{k}_{\boldsymbol{1}}$ | ${5 x 10}^{-3}$ | $\boldsymbol{k}_{\boldsymbol{26}}$ | ${2.25 x 10}^{-2}$ | $\boldsymbol{k}_{\boldsymbol{51}}$ | ${5 x 10}^{-8}$ |
| $\boldsymbol{k}_{\boldsymbol{2}}$ | ${5 x 10}^{-4}$ | $\boldsymbol{k}_{\boldsymbol{27}}$ | ${1.75 x 10}^{-4}$ | $\boldsymbol{k}_{\boldsymbol{52}}$ | ${5 x 10}^{-7}$ |
| $\boldsymbol{k}_{\boldsymbol{3}}$ | ${5 x 10}^{-3}$ | $\boldsymbol{k}_{\boldsymbol{28}}$ | ${1.9 x 10}^{-2}$ | $\boldsymbol{k}_{\boldsymbol{53}}$ | ${5 x 10}^{-5}$ |
| $\boldsymbol{k}_{\boldsymbol{4}}$ | ${2.5 x 10}^{-3}$ | $\boldsymbol{k}_{\boldsymbol{29}}$ | ${5 x 10}^{-4}$ | $\boldsymbol{k}_{\boldsymbol{54}}$ | ${1 x 10}^{-2}$ |
| $\boldsymbol{k}_{\boldsymbol{5}}$ | ${7.5 x 10}^{-2}$ | $\boldsymbol{k}_{\boldsymbol{30}}$ | ${2.5 x 10}^{-3}$ | $\boldsymbol{k}_{\boldsymbol{55}}$ | ${5 x 10}^{-8}$ |
| $\boldsymbol{k}_{\boldsymbol{6}}$ | ${2.5 x 10}^{-3}$ | $\boldsymbol{k}_{\boldsymbol{31}}$ | ${1.75 x 10}^{-4}$ | $\boldsymbol{k}_{\boldsymbol{56}}$ | ${5 x 10}^{-5}$ |
| $\boldsymbol{k}_{\boldsymbol{7}}$ | ${1.25 x 10}^{-3}$ | $\boldsymbol{k}_{\boldsymbol{32}}$ | ${2.5 x 10}^{-3}$ | $\boldsymbol{k}_{\boldsymbol{57}}$ | ${5 x 10}^{-3}$ |
| $\boldsymbol{k}_{\boldsymbol{8}}$ | ${2.5 x 10}^{-4}$ | $\boldsymbol{k}_{\boldsymbol{33}}$ | ${1.75 x 10}^{-4}$ | $\boldsymbol{k}_{\boldsymbol{58}}$ | ${5 x 10}^{-5}$ |
| $\boldsymbol{k}_{\boldsymbol{9}}$ | ${8 x 10}^{-4}$ | $\boldsymbol{k}_{\boldsymbol{34}}$ | ${5 x 10}^{-8}$ | $\boldsymbol{k}_{\boldsymbol{59}}$ | ${5 x 10}^{-4}$ |
| $\boldsymbol{k}_{\boldsymbol{10}}$ | ${5 x 10}^{-4}$ | $\boldsymbol{k}_{\boldsymbol{35}}$ | ${1 x 10}^{-2}$ | $\boldsymbol{k}_{\boldsymbol{60}}$ | ${1 x 10}^{-4}$ |
| $\boldsymbol{k}_{\boldsymbol{11}}$ | ${1 x 10}^{-3}$ | $\boldsymbol{k}_{\boldsymbol{36}}$ | ${1.5 x 10}^{-3}$ | $\boldsymbol{k}_{\boldsymbol{61}}$ | $1.5$ |
| $\boldsymbol{k}_{\boldsymbol{12}}$ | ${2 x 10}^{-4}$ | $\boldsymbol{k}_{\boldsymbol{37}}$ | ${5 x 10}^{-5}$ | $\boldsymbol{k}_{\boldsymbol{62}}$ | ${1 x 10}^{-3}$ |
| $\boldsymbol{k}_{\boldsymbol{13}}$ | ${5 x 10}^{-4}$ | $\boldsymbol{k}_{\boldsymbol{38}}$ | ${1 x 10}^{-2}$ | $\boldsymbol{k}_{\boldsymbol{63}}$ | ${9.4 x 10}^{-4}$ |
| $\boldsymbol{k}_{\boldsymbol{14}}$ | ${5 x 10}^{-4}$ | $\boldsymbol{k}_{\boldsymbol{39}}$ | ${5 x 10}^{-3}$ | $\boldsymbol{k}_{\boldsymbol{64}}$ | ${2 x 10}^{-2}$ |
| $\boldsymbol{k}_{\boldsymbol{15}}$ | ${5 x 10}^{-4}$ | $\boldsymbol{k}_{\boldsymbol{40}}$ | ${2 x 10}^{-3}$ | $\boldsymbol{k}_{\boldsymbol{65}}$ | $9.5$ |
| $\boldsymbol{k}_{\boldsymbol{16}}$ | ${5 x 10}^{-4}$ | $\boldsymbol{k}_{\boldsymbol{41}}$ | ${5 x 10}^{-5}$ | $\boldsymbol{k}_{\boldsymbol{66}}$ | $10$ |
| $\boldsymbol{k}_{\boldsymbol{17}}$ | ${2 x 10}^{-3}$ | $\boldsymbol{k}_{\boldsymbol{42}}$ | ${1 x 10}^{-4}$ | $\boldsymbol{k}_{\boldsymbol{67}}$ | ${5 x 10}^{-3}$ |
| $\boldsymbol{k}_{\boldsymbol{18}}$ | ${5 x 10}^{-4}$ | $\boldsymbol{k}_{\boldsymbol{43}}$ | ${5 x 10}^{-4}$ | $\boldsymbol{k}_{\boldsymbol{68}}$ | ${5 x 10}^{-2}$ |
| $\boldsymbol{k}_{\boldsymbol{19}}$ | ${5 x 10}^{-3}$ | $\boldsymbol{k}_{\boldsymbol{44}}$ | ${5 x 10}^{-4}$ | $\boldsymbol{k}_{\boldsymbol{69}}$ | ${8 x 10}^{-4}$ |
| $\boldsymbol{k}_{\boldsymbol{20}}$ | ${5 x 10}^{-4}$ | $\boldsymbol{k}_{\boldsymbol{45}}$ | ${5 x 10}^{-5}$ | $\boldsymbol{k}_{\boldsymbol{70}}$ | $6$ |
| $\boldsymbol{k}_{\boldsymbol{21}}$ | ${5 x 10}^{-3}$ | $\boldsymbol{k}_{\boldsymbol{46}}$ | ${2.5 x 10}^{-3}$ | $\boldsymbol{k}_{\boldsymbol{71}}$ | ${4 x 10}^{-3}$ |
| $\boldsymbol{k}_{\boldsymbol{22}}$ | ${2.5 x 10}^{-2}$ | $\boldsymbol{k}_{\boldsymbol{47}}$ | ${2.5 x 10}^{-3}$ | $\boldsymbol{k}_{\boldsymbol{72}}$ | ${1 x 10}^{-8}$ |
| $\boldsymbol{k}_{\boldsymbol{23}}$ | ${1.75 x 10}^{-3}$ | $\boldsymbol{k}_{\boldsymbol{48}}$ | ${2.5 x 10}^{-3}$ | $\boldsymbol{k}_{\boldsymbol{73}}$ | ${7.72 x 10}^{-1}$ |
| $\boldsymbol{k}_{\boldsymbol{24}}$ | ${2.25 x 10}^{-2}$ | $\boldsymbol{k}_{\boldsymbol{49}}$ | ${4 x 10}^{-2}$ | $\boldsymbol{k}_{\boldsymbol{74}}$ | ${5.56 x 10}^{-2}$ |
| $\boldsymbol{k}_{\boldsymbol{25}}$ | ${1.75 x 10}^{-4}$ | $\boldsymbol{k}_{\boldsymbol{50}}$ | ${2.5 x 10}^{-3}$ | $\boldsymbol{k}_{\boldsymbol{75}}$ | ${2 x 10}^{-2}$ |

## SI 4.3. Reactions in catalytic reaction network

| $\boldsymbol{R}_{\boldsymbol{1}}\boldsymbol{:}$ $\star\underset{\to}{k_{1}}x_{0}$ | $\boldsymbol{R}_{\boldsymbol{26}}\boldsymbol{:}$ $x_{7}+x_{4}\underset{\to}{k_{26}}x_{16}$ | $\boldsymbol{R}_{\boldsymbol{51}}\boldsymbol{:}$ $x_{21}\underset{\to}{k_{51}}x_{21}$ |
| --- | --- | --- |
| $\boldsymbol{R}_{\boldsymbol{2}}\boldsymbol{:}$ $x_{0}\underset{\to}{k_{2}}\star$ | $\boldsymbol{R}_{\boldsymbol{27}}\boldsymbol{:}$ $x_{16}\underset{\to}{k_{27}}x_{14}+x_{7}$ | $\boldsymbol{R}_{\boldsymbol{52}}\boldsymbol{:}$ $\star\underset{\to}{k_{52}}x_{21}$ |
| $\boldsymbol{R}_{\boldsymbol{3}}\boldsymbol{:}$ $x_{0}+x_{3}\underset{\to}{k_{3}}x_{5}$ | $\boldsymbol{R}_{\boldsymbol{28}}\boldsymbol{:}$ $x_{8}+x_{9}\underset{\to}{k_{28}}x_{9}$ | $\boldsymbol{R}_{\boldsymbol{53}}\boldsymbol{:}$ $x_{21}\underset{\to}{k_{53}}\star$ |
| $\boldsymbol{R}_{\boldsymbol{4}}\boldsymbol{:}$ $x_{5}\underset{\to}{k_{4}}x_{0}+x_{3}$ | $\boldsymbol{R}_{\boldsymbol{29}}\boldsymbol{:}$ $x_{9}\underset{\to}{k_{29}}x_{8}$ | $\boldsymbol{R}_{\boldsymbol{54}}\boldsymbol{:}$ $x_{21}+x_{9}\underset{\to}{k_{54}}\star$ |
| $\boldsymbol{R}_{\boldsymbol{5}}\boldsymbol{:}$ $x_{21}\underset{\to}{k_{5}}x_{1}$ | $\boldsymbol{R}_{\boldsymbol{30}}\boldsymbol{:}$ $x_{9}+x_{10}\underset{\to}{k_{30}}x_{13}$ | $\boldsymbol{R}_{\boldsymbol{55}}\boldsymbol{:}$ $x_{22}\underset{\to}{k_{55}}x_{23}$ |
| $\boldsymbol{R}_{\boldsymbol{6}}\boldsymbol{:}$ $x_{1}\underset{\to}{k_{6}}\star$ | $\boldsymbol{R}_{\boldsymbol{31}}\boldsymbol{:}$ $x_{13}\underset{\to}{k_{31}}x_{10}+x_{9}$ | $\boldsymbol{R}_{\boldsymbol{56}}\boldsymbol{:}$ $\star\underset{\to}{k_{56}}x_{23}$ |
| $\boldsymbol{R}_{\boldsymbol{7}}\boldsymbol{:}$ $x_{1}+x_{4}\underset{\to}{k_{7}}x_{6}$ | $\boldsymbol{R}_{\boldsymbol{32}}\boldsymbol{:}$ $x_{9}+x_{14}\underset{\to}{k_{32}}x_{17}$ | $\boldsymbol{R}_{\boldsymbol{57}}\boldsymbol{:}$ $x_{23}\underset{\to}{k_{57}}\star$ |
| $\boldsymbol{R}_{\boldsymbol{8}}\boldsymbol{:}$ $x_{6}\underset{\to}{k_{8}}x_{1}+x_{4}$ | $\boldsymbol{R}_{\boldsymbol{33}}\boldsymbol{:}$ $x_{17}\underset{\to}{k_{33}}x_{14}+x_{9}$ | $\boldsymbol{R}_{\boldsymbol{59}}\boldsymbol{:}$ $x_{18}\underset{\to}{k_{59}}x_{23}$ |
| $\boldsymbol{R}_{\boldsymbol{9}}\boldsymbol{:}$ $x_{26}\underset{\to}{k_{9}}x_{2}$ | $\boldsymbol{R}_{\boldsymbol{34}}\boldsymbol{:}$ $\star\underset{\to}{k_{34}}x_{10}$ | $\boldsymbol{R}_{\boldsymbol{60}}\boldsymbol{:}$ $\star\underset{\to}{k_{60}}x_{24}$ |
| $\boldsymbol{R}_{\boldsymbol{10}}\boldsymbol{:}$ $x_{2}\underset{\to}{k_{10}}\star$ | $\boldsymbol{R}_{\boldsymbol{35}}\boldsymbol{:}$ $x_{7}+x_{10}\underset{\to}{k_{35}}\star$ | $\boldsymbol{R}_{\boldsymbol{61}}\boldsymbol{:}$ *Signal* $\underset{\to}{k_{61}}x_{24}$ |
| $\boldsymbol{R}_{\boldsymbol{11}}\boldsymbol{:}$ $x_{2}+x_{4}\underset{\to}{k_{11}}x_{8}$ | $\boldsymbol{R}_{\boldsymbol{36}}\boldsymbol{:}$ $x_{9}+x_{10}\underset{\to}{k_{36}}\star$ | $\boldsymbol{R}_{\boldsymbol{62}}\boldsymbol{:}$ $x_{24}\underset{\to}{k_{62}}\star$ |
| $\boldsymbol{R}_{\boldsymbol{12}}\boldsymbol{:}$ $x_{8}\underset{\to}{k_{12}}x_{2}+x_{4}$ | $\boldsymbol{R}_{\boldsymbol{37}}\boldsymbol{:}$ $\star\underset{\to}{k_{37}}x_{14}$ | $\boldsymbol{R}_{\boldsymbol{63}}\boldsymbol{:}$ $\star\underset{\to}{k_{63}}x_{25}$ |
| $\boldsymbol{R}_{\boldsymbol{13}}\boldsymbol{:}$ $x_{5}\underset{\to}{k_{13}}x_{3}$ | $\boldsymbol{R}_{\boldsymbol{38}}\boldsymbol{:}$ $x_{24}\underset{\to}{k_{38}}x_{14}$ | $\boldsymbol{R}_{\boldsymbol{64}}\boldsymbol{:}$ $x_{25}\underset{\to}{k_{64}}\star$ |
| $\boldsymbol{R}_{\boldsymbol{14}}\boldsymbol{:}$ $x_{9}\underset{\to}{k_{14}}x_{4}$ | $\boldsymbol{R}_{\boldsymbol{39}}\boldsymbol{:}$ $x_{14}\underset{\to}{k_{39}}\star$ | $\boldsymbol{R}_{\boldsymbol{65}}\boldsymbol{:}$ $x_{27}^{9}\underset{\to}{k_{65}}x_{25}$ |
| $\boldsymbol{R}_{\boldsymbol{15}}\boldsymbol{:}$ $x_{8}\underset{\to}{k_{15}}x_{4}$ | $\boldsymbol{R}_{\boldsymbol{40}}\boldsymbol{:}$ $\star\underset{\to}{k_{40}}x_{18}$ | $\boldsymbol{R}_{\boldsymbol{66}}\boldsymbol{:}$ $x_{27}^{9}\underset{\to}{k_{66}}x_{25}$ |
| $\boldsymbol{R}_{\boldsymbol{16}}\boldsymbol{:}$ $x_{6}\underset{\to}{k_{16}}x_{4}$ | $\boldsymbol{R}_{\boldsymbol{42}}\boldsymbol{:}$ $x_{23}\underset{\to}{k_{42}}x_{18}$ | $\boldsymbol{R}_{\boldsymbol{67}}\boldsymbol{:}$ $x_{27}\underset{\to}{k_{67}}\star$ |
| $\boldsymbol{R}_{\boldsymbol{17}}\boldsymbol{:}$ $x_{7}.x_{7}\underset{\to}{k_{17}}x_{4}$ | $\boldsymbol{R}_{\boldsymbol{43}}\boldsymbol{:}$ $x_{18}\underset{\to}{k_{43}}\star$ | $\boldsymbol{R}_{\boldsymbol{68}}\boldsymbol{:}$ $x_{21}\underset{\to}{k_{68}}x_{26}$ |
| $\boldsymbol{R}_{\boldsymbol{18}}\boldsymbol{:}$ $x_{5}+x_{14}\underset{\to}{k_{18}}x_{15}$ | $\boldsymbol{R}_{\boldsymbol{44}}\boldsymbol{:}$ $x_{18}+x_{5}\underset{\to}{k_{44}}\star$ | $\boldsymbol{R}_{\boldsymbol{69}}\boldsymbol{:}$ $x_{26}\underset{\to}{k_{69}}\star$ |
| $\boldsymbol{R}_{\boldsymbol{19}}\boldsymbol{:}$ $x_{15}\underset{\to}{k_{19}}x_{14}+x_{5}$ | $\boldsymbol{R}_{\boldsymbol{45}}\boldsymbol{:}x_{21}+x_{23}\underset{\to}{k_{45}}x_{19}$ | $\boldsymbol{R}_{\boldsymbol{70}}\boldsymbol{:} x_{24}+$ *Signal* $\underset{\to}{k_{70}}x_{27}$ |
| $\boldsymbol{R}_{\boldsymbol{20}}\boldsymbol{:}$ $x_{5}+x_{10}\underset{\to}{k_{20}}x_{11}$ | $\boldsymbol{R}_{\boldsymbol{46}}\boldsymbol{:}$ $x_{19}+x_{5}\underset{\to}{k_{46}}x_{20}$ | $\boldsymbol{R}_{\boldsymbol{71}}\boldsymbol{:}$ $x_{24}+x_{25}\underset{\to}{k_{71}}x_{27}$ |
| $\boldsymbol{R}_{\boldsymbol{21}}\boldsymbol{:}$ $x_{11}\underset{\to}{k_{21}}x_{10}+x_{5}$ | $\boldsymbol{R}_{\boldsymbol{47}}\boldsymbol{:}$ $x_{11}+x_{19}\underset{\to}{k_{47}}x_{20}$ | $\boldsymbol{R}_{\boldsymbol{72}}\boldsymbol{:}$ $DDS\underset{\to}{exp(-k_{72}.t)}$ *Signal* |
| $\boldsymbol{R}_{\boldsymbol{22}}\boldsymbol{:}$ $x_{6}+x_{7}\underset{\to}{k_{22}}x_{7}$ | $\boldsymbol{R}_{\boldsymbol{48}}\boldsymbol{:}$ $x_{19}+x_{15}\underset{\to}{k_{48}}x_{20}$ | $\boldsymbol{R}_{\boldsymbol{73}}\boldsymbol{:}$ $x_{24}+x_{25}\underset{\to}{k_{73}}\star$ |
| $\boldsymbol{R}_{\boldsymbol{23}}\boldsymbol{:}$ $x_{7}\underset{\to}{k_{23}}x_{6}$ | $\boldsymbol{R}_{\boldsymbol{49}}\boldsymbol{:}$ $x_{7}+x_{20}\underset{\to}{k_{49}}x_{21}$ | $\boldsymbol{R}_{\boldsymbol{74}}\boldsymbol{:}$ $x_{24}\underset{\to}{k_{74}}\star$ |
| $\boldsymbol{R}_{\boldsymbol{24}}\boldsymbol{:}$ $x_{7}+x_{10}\underset{\to}{k_{24}}x_{12}$ | $\boldsymbol{R}_{\boldsymbol{50}}\boldsymbol{:}$ $x_{9}+x_{20}\underset{\to}{k_{50}}x_{21}$ | $\boldsymbol{R}_{\boldsymbol{75}}\boldsymbol{:} x_{24}+{DDS}_{initial}\underset{\to}{k_{75}}\star$ |
| $\boldsymbol{R}_{\boldsymbol{25}}\boldsymbol{:}$ $x_{12}\underset{\to}{k_{25}}x_{10}+x_{7}$ |  |  |

# **SI 5. Description of modules, sub-modules present in**$\boldsymbol{ISP}$

The $ISP$ has six major modules (*steps*): (i) state module; (ii) explore module; (iii) sort module; (iv) dictionary module; (v) update module; and (vi) queue/stack module. The sub-modules are shown in Table SI 7.

**Table SI 7. Modules/sub-modules, components of the comprehensive** $\boldsymbol{ISP}$ **method.**

| **Module (Steps)** | **Sub-module (Steps)** | **Components** |
| --- | --- | --- |
| Input module | $-$ | $N_{0}$, $a_{\mu}$, $V_{\mu}$, $\tau_{m}$, $t_{f}$, $t_{step}$ |
|  | Provide inputs to the sub-modules at $t_{d}$ | |
| State module | $N_{i}=\left( X_{0},ƌ_{l} \right)$ | $N_{i}$, $X_{0}$, $ƌ_{l}$, $t_{d}$ |
|  | Describes the graph initial node and corresponding set of states at $t_{d}$ | |
| Explore module | $1-I^{T}exp\left( t.A_{j} \right). P^{(t)}\left( X_{0} \right)\geq\tau_{m}(leak)$  or  $1-I^{T}exp\left( t_{f}A_{j} \right). P^{\left( t \right)}\left( X_{0} \right)\geq\tau_{m}*\frac{(no. of R_{M(sr)})}{(no. of R_{M(fs)})}$  or  $1-I^{T}exp\left( t_{f}A_{j} \right). P^{\left( t \right)}\left( X_{0} \right)\geq\tau_{m}*\frac{(no. of R_{M(fs)})}{(no. of R_{M(sr)})}$ | $A_{j}$, $exp$, $X_{i}$, $X_{0}$, $\tau_{m}$, $t$, $I^{T}$ |
|  | Defines the validation functions for exploration at $t$ | |
| Sort module | - $exp\left( t.A_{j} \right). P^{(t)}\left( X_{0} \right)$ - $P^{\left( t \right)}\left( \mathbf{X}_{K} \right)\geq\tau_{m}(leak)>P^{(t)}\left( \mathbf{X}_{K}^{'} \right)$ - $\mathbf{X}_{K}⟵\mathbf{X}_{K}-\mathbf{X}_{K}^{'}$ | $A_{j}$, $exp$, $X_{i}$, $X_{0}$, $\tau_{m}(leak)$, $t$, $\mathbf{X}_{K}$, $\mathbf{X}_{K}^{'}$ |
|  | Follows the sorting and bunking of $P^{(t)}\left( \mathbf{X}_{K}^{'} \right)$ | |
| Dictionary module | - $Dict$ - $\mathbf{n}_{K}=\left( \mathbf{X}_{K},ƌ_{l}, Ͼ_{N_{i},N_{i}^{'}}(min) \right)$ | $Dict$, $\mathbf{X}_{K}$, $ƌ_{l}$, $\mathbf{n}_{K}$, $R_{M}$, $Ͼ_{N_{i},N_{i}^{'}}(min)$ |
|  | Accounts for mapping of nodes by keeping the track of transitions at $t$. | |
| Update module | - $\mathbf{n}_{K}=\left( \mathbf{X}_{K},ƌ_{l}, Ͼ_{N_{i},N_{i}^{'}}(min) \right)\in domain$, - ${domain⟵domain}_{previous}\cup domain$, - $\mathbf{n}_{K}=\left( \mathbf{X}_{K},ƌ_{l}, Ͼ_{N_{i},N_{i}^{'}}(min) \right)\notin domain$, - $b\left( N_{N1,..NM} \vert b_{1,N\ldots.N^{'},N}^{'} \right)$ - $queue$ or $stack$. | $\mathbf{X}_{K}$, $ƌ_{l}$, $\mathbf{n}_{K}$, $R_{M}$, $Ͼ_{N_{i},N_{i}^{'}}(min)$, $P_{N,N^{'}}(\omega)$, $b_{N^{'},N}^{'}$, $queue$ or $stack$, ${domain}_{previous}$. |
|  | Responsible for updating the states in the domain; and pre-conditioning at $t$. | |
| Queue or stack module | - $\mathbf{n}_{K}=\left( \mathbf{X}_{K},ƌ_{l}, Ͼ_{N_{i},N_{i}^{'}}(min) \right)$, - $queue$ or $stack$ | $\mathbf{X}_{K}$, $ƌ_{l}$, $\mathbf{n}_{K}$, $R_{M}$, $Ͼ_{N_{i},N_{i}^{'}}(min)$, $queue$ or $stack$, ${domain}_{previous}$. |
|  | Consider new $\mathbf{n}_{K}$ unique set of nodes for addition in the domain. | |
| Bound module | - $count\left( ƃ_{limit} \right)=ƃ_{limit}$, - ${Bound}_{upper}=\left\{ domain \right\}$, - ${Bound}_{lower}\leftarrow{Bound}_{upper}$, - $count\left( ƃ_{limit} \right)<ƃ_{limit}$. | $count\left( \boldsymbol{ƃ}_{limit} \right)$, $\boldsymbol{ƃ}_{limit}$, ${Bound}_{lower}$, ${Bound}_{upper}.$ |
|  | Defines ${Bound}_{lower}$ for next iteration and conditions if $count\left( ƃ_{limit} \right)$ reaches the $ƃ_{limit}$. | |

The *state module* describes the initial node of the graph and maps with the corresponding set of states at any time, $t_{d}$. It takes the initial values of the components from the input module and stores the information of a number of nodes, sets of states, depth of nodes in the state-space or the bound limit in every iteration.

The *explore module* defines the validation functions for exploration. At $t$ it flags the current node to be explored, and looks for a set of child nodes carrying a set of states, as long as the validation Eq. (32) (or Eq. (30) or (31) based on conditions) holds true; otherwise it stops the exploration. The cease of validation defines the stopping criteria of the exploration.

The *sort module* organises the probabilities based on the conditions of the sub-module (as shown in Table SI 7) and bunks the probabilities that provide minimal weightage in the approximation as it is computationally expensive to re-compute these probabilities up to $t_{f}$.

The *dictionary module* keeps track of adjacent sets of $\mathbf{n}_{K}\in\mathbf{n}_{J}$ nodes in real-time and walks between them. The dictionary $Dict$ is a compressed row format that records different nodes in a single transition, but uses a similar $\mathbf{n}_{K}$ set of nodes in all possible transitions.

The *update module* is responsible for pre-conditioning and updating the domain with the new set of new states and validating it for duplicate states as a result of the previous iterations. The corresponding set of nodes carrying a set of states are not considered if the states are already present in the domain; however, the changes in propensity values are updated.

The *queue* and *stack* module provides a unique node to the successor operator Eq. (33) to consider new states by pulling the node out from the *queue* in $LAS$ or by popping the top node from the *stack* in $LOLAS$. In $LAS$, these new states act as an input to the *state module* for the next iteration, as well as for approximation methods while, in $LOLAS$, it goes through a *bound module*, which defines the upper and lower bounds of the iteration and the bound limit that satisfies the sub-module conditions.

These modules and sub-modules constitute the $ISP$ method, such that they track key changes in the components that follows the changes in the reaction propensities by population and activation of the species and describes the dynamics of the biochemical system. This method also permits the time form quantification of the state-space based on the size and dimensions of the model. Further details of the method conditions are discussed in the following sections.

# **SI 6. Dilemma in selection**

**(a) Statement of the Problem:** Assume that ${state\left( N_{0} \right)=X}_{0}$ is the present state of the system, and further we have two choices to make among ${state\left( N_{1} \right)=X}_{1}$ and ${state\left( N_{2} \right)=X}_{2}$ based on reactions and copy counts (as shown in Figure SI 11) for the future state of the system.

It is clear that species with higher copy counts will interact quickly compared to other species with lower copy counts. Depending upon the association of higher copy count species within reactions $R_{1}$ or $R_{2}$, the system will jump to $X_{1}$ or $X_{2}$ respectively. Now we make an assumption that there are almost equal propensities of reactions, as shown in Figure SI 12, at any time $t$ of the expansion phase.


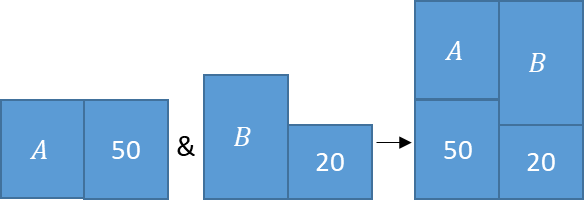


**Figure SI 11.** $\boldsymbol{A}$ **and** $\boldsymbol{B}$ **with copy counts** $\boldsymbol{50}$ **and** $\boldsymbol{20}$**, respectively, creating a system of two species,** $\boldsymbol{A}$ **and** $\boldsymbol{B}$ **with 70, total copy counts**.


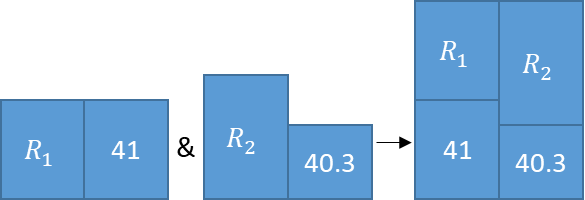


**Figure SI 12. Two states** $\boldsymbol{X}_{\boldsymbol{1}}$ **of** $\boldsymbol{A}$ **and** $\boldsymbol{X}_{\boldsymbol{2}}$ **of** $\boldsymbol{B}$ **with almost equal propensities. This creates a dilemma when choosing the direction for expansion**.

**(b) Real life example:** Suppose a person walking from the car park to the entrance of the fifa world cup stadium and accidently dropped their ticket in a hurry. From behind we can see that the person has long hairs, but we are not sure whether they are man or women and what to call out "Excuse me ma'am!" or "Excuse me Sir!". In such situation we have to make a guess.

Now imagine instead this person has reached the entrance and standing in the queue of men’s. Knowing this piece of extra information we might make a different guess. $BLNP$ is a way to capture the sense of knowledge about the situation to make better guesses as it treats the probability as beliefs, not frequencies. Now we put numbers to our dilemma at the queue as shown in the Figure SI 13. Let us assume that out of 1000 women in the queue 500 have short hair and 50 have long and out of 1000 men in the queue 940 have short hair and 60 have long. In this case we can see that definitely there are more women with long hair than men with long hair, so it is a safe bet to assume it is a women.


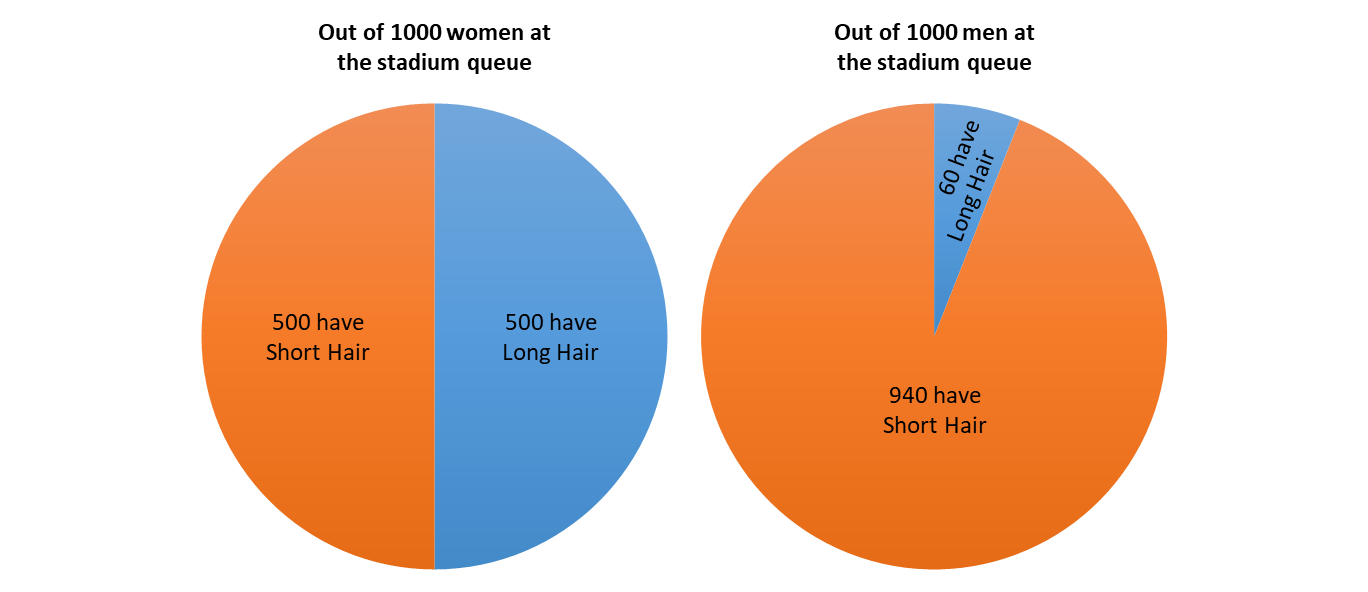


**Figure SI 13. Showing the ratio of distribution of men and women.**

Now we just made a subtle assumption that there are about the same number of women and men in the queue of a stadium. This assumption no longer holds when we move to the men's queue. Here let's say there are 20 women out of every 1000 people and 980 are men, as maybe women keeping their partners company. Figure SI 14 shows that, there still ten with short hairs and 10 with long hairs, it's still half and half long and short hair but now there are six times as many men with long hair than women.


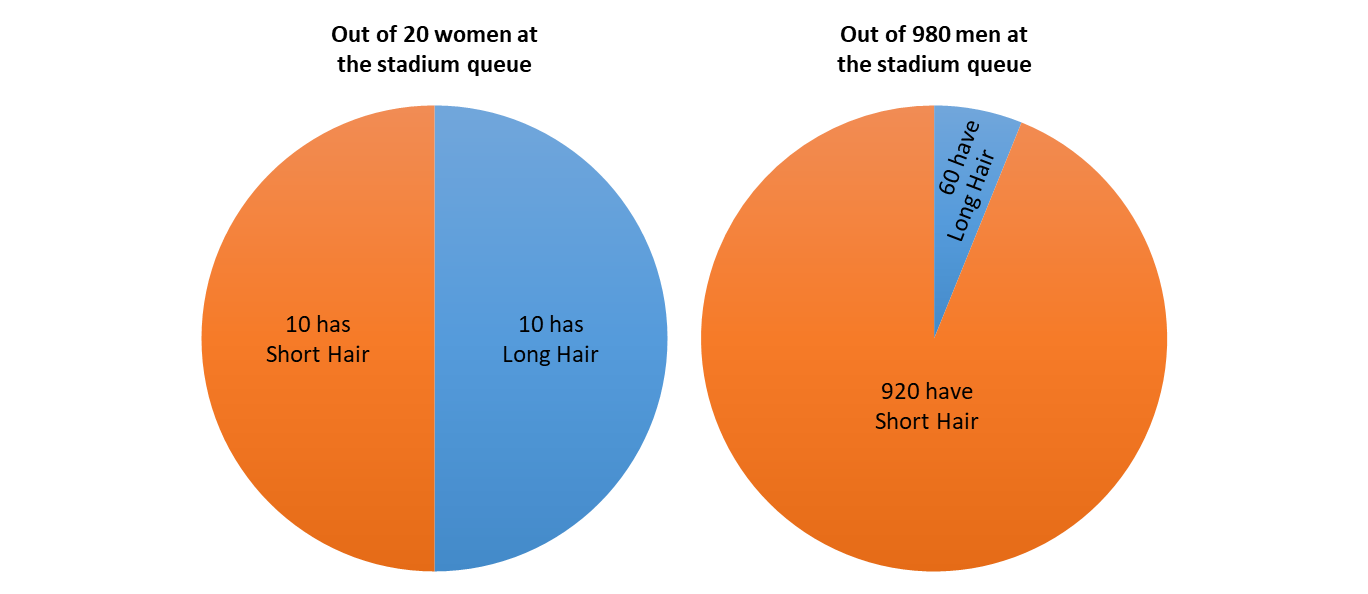


**Figure SI 14. Showing the distribution of men and women with short and long hairs.**

Now how we can make safe bet that this person is a man? So to draw this little differently, out of thousand people at the stadium queue overall, we make this assumption explicit that 500 of them are women and 500 of them are men as shown in Figure SI 15 (left and right). It shows how different categories break down in the queue for the men's queue then they break down little differently as shown in Figure SI 15.


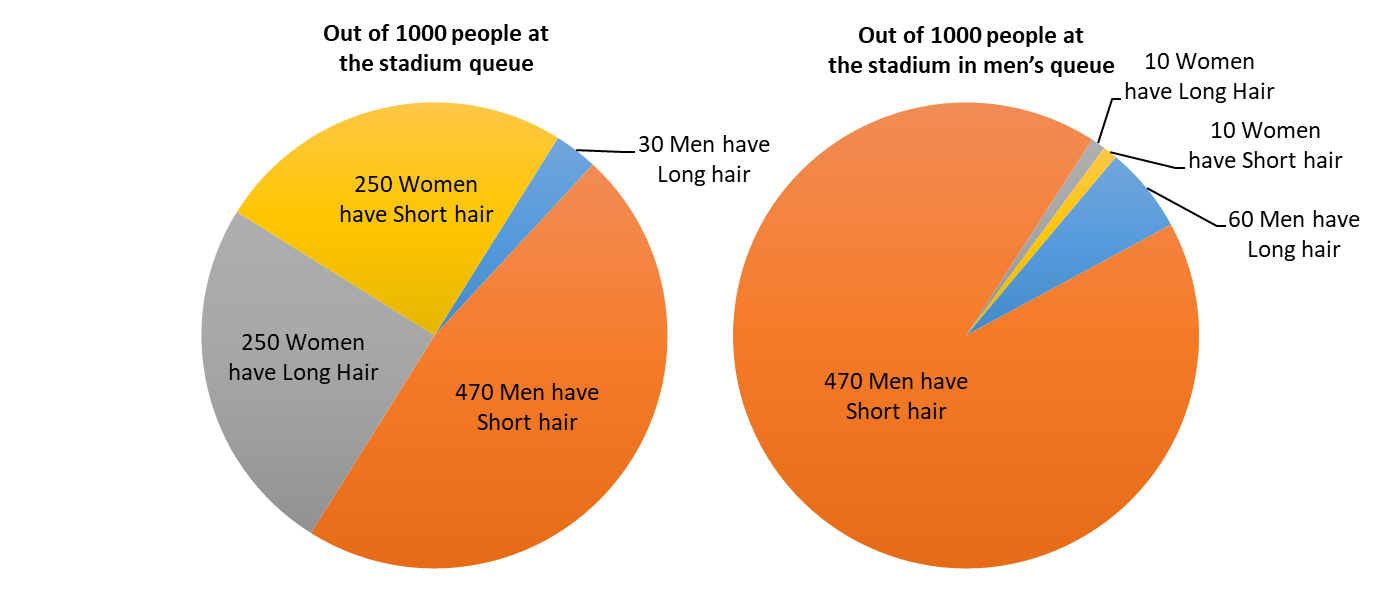


**Figure SI 15. Showing different distribution fig 42 (left), fig 42 (right) of men and women in a queue of *1000* members.**

From Figure SI 13, the probability that person is a woman and men are given by $P\left( w \right)=0.5$ and $P\left( men \right)=0.5$ respectively, whereas $P\left( women \right)=0.02$ and $P\left( men \right)=0.98$.

**Conditional probability:** If we know that a person is a woman that is the condition, then the probability that the person has long hair is given by $P\left( long hair|women \right)=0.5$. Similarly, for men the conditional probability that person has long hair is given by

$P\left( long hair|men \right)=0.06$. For Figure SI 12, the conditional probability

$P\left( long hair|women \right)=0.5$ and $P\left( long hair|men \right)=0.12$.

**Joint probability:** From Figure SI 12, the probability that a person is both a woman and has short hair is given by

$$P\left( women with short hair \right)=P\left( women \right)* P\left( short hair|women \right)$$

$=0.5*0.5=0.25.$

Similarly, the probability that a person is both woman and has long hair is given by

$P\left( women with long hair \right)=P\left( w \right)* P\left( long hair|women \right)$

$=0.5*0.5=0.25.$

The probability that a person is both man and has short hair is given by

$P\left( men with short hair \right)=P\left( men \right)*P\left( short hair|men \right)$

$=0.5*0.94=0.47.$

Similarly, the probability that a person is both man and has long hair is given by

$P\left( men with long hair \right)=P\left( men \right)* P\left( long hair|men \right)$

$=0.5*0.06=0.03.$

From Figure D.3, the probability that a person is both a woman and has short hair is given by

$P\left( women with short hair \right)=P\left( women \right)* P\left( short hair|women \right)$

$=0.02*0.5=0.01.$

Similarly, the probability that a person is both woman and has long hair is given by

$P\left( women with long hair \right)=P\left( women \right)* P\left( long hair|women \right)$

$=0.02*0.5=0.01.$

The probability that a person is both man and has short hair is given by

$P\left( men with short hair \right)=P\left( men \right)*P\left( short hair|men \right)$

$=0.98*0.94=0.9212.$

Similarly, the probability that a person is both man and has long hair is given by

$P\left( men with long hair \right)=P\left( men \right)* P\left( long hair|men \right)$

$=0.98*0.12=0.1176.$

**Marginal probability:** From Figure SI 13, the probability that someone has long hair is given by

$$P\left( long hair \right)=P\left( women with long hair \right)+P\left( men with long hair \right)$$

$= 0.01+0.1176=0.1276$

and for short hair

$$P\left( short hair \right)=P\left( women with short hair \right)+P\left( men with short hair \right)$$

$= 0.01+0.9212=0.9312$

If someone has long hairs the probability that they are a man can be calculated by the function

$$P\left( men | long hair \right)=\frac{P\left( men \right)*P\left( long hair \right| men)}{P(long hair)}$$

$= 0.9216$ or $92.16\%$.

Similarly, if someone has long hairs the probability that they are a women can be calculated as

$$P\left( women | long hair \right)=\frac{P\left( women \right)*P\left( long hair \right| women)}{P(long hair)}$$

$= 0.0783$ or $07.83\%$.

It is clear from the probability that the ticket dropped by the person is a man. Similarly, such function can be applied to the state-space to choose the probable states carrying most of the probability density and track the active reactions in the biochemical systems.

# **SI 7. Complexity based on operations**

The time complexity of $ISP$ can be evaluated for the worst-case scenario by considering the steps of $LAS$ and $LOLAS$ based on operations and by making some simplified assumptions on size of state-space. To execute each of these steps instructions machine takes discrete amount of time as,

1. In *step 1*, as the initial values are given for the first iteration, it will access the array inputs as:

for 1^st^ iteration it runs for 1 time,

for 2^nd^ iteration it will again run for 1 time,

for $I_{tr}$^th^ iteration it will run for 1 time.

Total number of times this step will run is the sum of all the run from 1^st^ to $I_{tr}$^th^ iteration as $1+1+1\ldots\ldots\ldots..+\left( I_{tr}-1 \right)=I_{tr}$, so the time complexity will simply be $O$($I_{tr}$). If $I_{tr}$ is equal to the set of nodes in the graph then complexity will become $O$($\mathbf{n}_{K}$) with the condition that all the nodes are considered as initial node at some point of any iteration, which is true in most of the cases.

1. In *step 2*, for initial node one-time flagging is done for set of states and updates the queue for 1 time as a set. It will continue to flag for other nodes expanded in iterations as:

for 2^nd^ iteration it will again run for 1 time having random number of states,

for $I_{tr}$^th^ iteration it will run for 1 time having end state of the system within final set of states.

Total number of times this step will run is the sum of all the iterations up to $I_{tr}$^th^. On an average if $\mathbf{n}_{K}$ are carrying $S^{Ñ}$ states upto $ƌ_{l}$ level then number of states added to the domain will be (${\mathbf{n}_{K}}^{(S^{Ñ})}$) and complexity will be $O$(${\mathbf{n}_{K}}^{(\mathbf{X}_{K})}$). The calculation of Eq. (33) will add constant factor to the complexity, so $\mathbf{n}_{K}$ carrying set of $\mathbf{X}_{K}$ will have complexity of $O({\mathbf{n}_{K}}^{(\mathbf{X}_{K})})$. Most of the time, the condition of Eq. (33) will be true as long as $\mathbf{X}_{K}$ is getting new states.

1. In *step 3*, sorting the probabilities in every iteration when number of states is growing with exploration leads to the time complexity which depends only on size of $\mathbf{X}_{K}$, whereas bunking of states with low probabilities from the $queue$ done conditionally in selective iteration at $t^{'}$, so for

for 1^st^ iteration sorting $\mathbf{X}_{K}$ and bunking of states is done 0 times, so the complexity will be 0,

for 2^nd^ iteration sorting $\mathbf{X}_{K}$ is done 1 time but bunking is based on Eq. (71), so the complexity will be $O$($1^{2}$) + 0 = $O$($1$), for $I_{tr}$^th^ iteration sorting is done $I_{tr}$ times; so complexity will be $O$(${I_{tr}}^{2}$) and if the bunking is done it will add $O$($I_{tr}logI_{tr}$) of complexity. So the complexity of $I_{tr}$^th^ iteration will become $O$(${I_{tr}}^{2}$) + $O$($I_{tr}logI_{tr}$).

The total complexity of the step is the sum of 0 + $O$($1^{2}$) + 0 +,…………….,+$O$(${I_{tr}}^{2}$) + $O$($I_{tr}logI_{tr}$). If any biochemical system has $\mathbf{X}_{K}$ set of states then the complexity $O$($I_{tr}logI_{tr}$) becomes negligible as the size of set of states $\mathbf{X}_{K}^{'}$ is smaller than the size of the $\mathbf{X}_{K}$ when Eq. (71) applied conditionally. Therefore, the complexity of the step reduce to $O$(${I_{tr}}^{2}$) if $O$($I_{tr}logI_{tr}$) is ignored for most of the cases.

1. In *step 4*, searching adjacent set of nodes $\mathbf{n}_{K}$ to current node $N_{i}$ to extend the dictionary $Dict$ will incur $O$(1) for single reaction for one step as one new node is added carrying new states every time, so complexity for every node for $I_{tr}$ iterations will be $O$($\mathbf{n}_{K}$) and further computing the $BLNP$ function for $\mathbf{n}_{K}$ will add $O$($\mathbf{n}_{K}$) to the complexity. Overall, it will give the complexity of Ѭ for complete search.
2. In step 5, accessing the array (domain) in $I_{tr}$ iterations will cost $O$($I_{tr}$) or $O$($\mathbf{n}_{K}$) = $O$($\mathbf{X}_{K}$). To commit the updates of propensities of set of states will cost $O$($1$) per value, i.e. for $O$($X_{0},X_{1},\ldots\ldots..$) = $O$($\mathbf{X}_{K}$) if ${\mathbf{n}_{K}=(X}_{0,1,2\ldots\ldots}, ƌ_{l})\notin domain$ then complexity will be $O$($1$) per value in the queue or stack, so for $\mathbf{X}_{K}$ it will run $Z$ times, $O$($I_{tr}$).
3. In step 6, accessing ${\mathbf{n}_{K}=(X}_{0,1,2\ldots\ldots}, ƌ_{l})$ for each value from the queue or stack will have same complexity of $O$($\mathbf{n}_{K}$) for each $N_{i}$ for $I_{tr}$ iterations, whereas adding the new set of states in the domain will be $O$($|{\mathbf{n}_{K}}^{(\mathbf{X}_{K})}|$).

Combining all the steps complexity,

= $O(|\mathbf{n}_{K}|)$ + $O$($|{\mathbf{n}_{K}}^{\left( \mathbf{X}_{K} \right)}|$) + $O$($|{I_{tr}}^{2}|$) + $O$($|I_{tr}logI_{tr}|$) + $O$($|\mathbf{n}_{K}|$) + $O$($|\mathbf{X}_{K}|$) + $O$($|I_{tr}|$) + $O$($|\mathbf{n}_{K}|$) + $O$($|{\mathbf{n}_{K}}^{(\mathbf{X}_{K})}|$)

**Step 1 Step 2 Step 3 Step 4 Step 5 Step 6**

ignoring $O$($I_{tr}logI_{tr}$) as it becomes negligible when no states were bunked from the domain or number of states bunked from domain were much lesser than the number of states present in the domain. If estimated number of states $S^{Ñ}$ creates the perfect domain with set $\mathbf{X}_{K}$ then total complexity will reduce to

= 3 × $O(\mathbf{n}_{K})$ + 2 x $O$(${\mathbf{n}_{K}}^{(\mathbf{X}_{K})}$) + $O$(${I_{tr}}^{2}$) + $O$($I_{tr}$) + $O$($\mathbf{X}_{K}$),

therefore, $O\left( Algo\left( y \right) \right)=\{0\leq f\left( y \right)\leq c*Algo(y)$ $for all y\geq y_{0}\}$,

where $y, y_{0}$ are positive integers. When working with biochemical networks graphs that are large to store explicitly, it is pragmatic to define the overall complexity of the $ISP$in terms of states and depth of the explored states. As we are interested in end state, so for any iteration if the end state of the system is at $ƌ_{l}$ from initial node $N_{0}$ state $X_{0}$ then for $I_{tr}$ iterations $O$(${\mathbf{n}_{K}}^{(\mathbf{X}_{K})}$) = $O(Ŧ^{ƌ_{l}})$, where $Ŧ$ is the average transitioning factor that defines the average number of transition from $N_{1}$ to $N_{N'}$. For small biochemical models every state in the system is crucial as there will be a smaller number of variables that may greatly affect the state-space of the system. In $ISP$, we increase the depth level by one after the end node has been found to track any reversible reaction that may take the system to previous state and add the end state present with end node, therefore the time complexity of the $ISP LAS$ becomes $O(Ŧ^{ƌ_{l}+1})$. The complexity of the operations carried out in $ISP$ variants for Markov chain tree of a biochemical network is discussed below:

**Table SI 8. Comparison of the** $\boldsymbol{ISP LAS}$ **average-case** $\boldsymbol{\Theta}$ **and worst-case** $\boldsymbol{O}$ ***Time-Space* complexity based on operations (*Access, Search, Update, and Remove*) on elements (*Array, Queue, and Tree*).**

| $\boldsymbol{LAS}$ | **Array** | **Queue** | **Tree** |
| --- | --- | --- | --- |
| Time complexity: Average case | | | |
| Access | $\Theta(1)$ | $\Theta(Ŧ^{ƌ_{l}+1})$ | $\Theta(log\left( Ŧ^{ƌ_{l}+1} \right))$ |
| Search | $\Theta(Ŧ^{ƌ_{l}+1})$ | $\Theta(Ŧ^{ƌ_{l}+1})$ | $\Theta(log\left( Ŧ^{ƌ_{l}+1} \right))$ |
| Update | $\Theta(Ŧ^{ƌ_{l}+1})$ | $\Theta(1)$ | $\Theta(log\left( Ŧ^{ƌ_{l}+1} \right))$ |
| Remove | $\Theta(Ŧ^{ƌ_{l}+1})$ | $\Theta(1)$ | $\Theta(log\left( Ŧ^{ƌ_{l}+1} \right))$ |
| Time complexity: Worst case | | | |
| Access | $O(1)$ | $O(Ŧ^{ƌ_{l}+1})$ | $O(Ŧ^{ƌ_{l}+1})$ |
| Search | $O(Ŧ^{ƌ_{l}+1})$ | $O(Ŧ^{ƌ_{l}+1})$ | $O(Ŧ^{ƌ_{l}+1})$ |
| Update | $O(Ŧ^{ƌ_{l}+1})$ | $O(1)$ | $O(Ŧ^{ƌ_{l}+1})$ |
| Remove | $O(Ŧ^{ƌ_{l}+1})$ | $O(1)$ | $O(Ŧ^{ƌ_{l}+1})$ |
| Space complexity: Worst case | | | |
| Space | $O(Ŧ^{ƌ_{l}+1})$ | $O(Ŧ^{ƌ_{l}+1})$ | $O(Ŧ^{ƌ_{l}+1})$ |

**Table SI 9.** **Comparison of the** $\boldsymbol{ISP LOLAS}$ **average-case** $\boldsymbol{\Theta}$ **and worst-case** $\boldsymbol{O}$ **Time-Space complexity based on operations (*Access, Search, Update, and Remove*) on elements (*Array, Stack, and Tree*).**

| $\boldsymbol{LOLAS}$ | **Array** | **Stack** | **Tree** |
| --- | --- | --- | --- |
| Time complexity: Average case | | | |
| Access | $\Theta(1)$ | $\Theta(Ŧ^{ƌ_{l}})$ | $\Theta(log\left( Ŧ^{ƌ_{l}} \right))$ |
| Search | $\Theta(Ŧ^{ƌ_{l}})$ | $\Theta(Ŧ^{ƌ_{l}})$ | $\Theta(log\left( Ŧ^{ƌ_{l}} \right))$ |
| Update | $\Theta(Ŧ^{ƌ_{l}})$ | $\Theta(1)$ | $\Theta(log\left( Ŧ^{ƌ_{l}} \right))$ |
| Remove | $\Theta(Ŧ^{d})$ | $\Theta(1)$ | $\Theta(log\left( Ŧ^{ƌ_{l}} \right))$ |
| Time complexity: Worst case | | | |
| Access | $O(1)$ | $O(Ŧ^{ƌ_{l}})$ | $O(Ŧ^{ƌ_{l}})$ |
| Search | $O(Ŧ^{ƌ_{l}})$ | $O(Ŧ^{ƌ_{l}})$ | $O(Ŧ^{ƌ_{l}})$ |
| Update | $O(Ŧ^{ƌ_{l}})$ | $O(1)$ | $O(Ŧ^{ƌ_{l}})$ |
| Remove | $O(Ŧ^{ƌ_{l}})$ | $O(1)$ | $O(Ŧ^{ƌ_{l}})$ |
| Space complexity: Worst case | | | |
| Space | $O(Ŧƌ_{l})$ | $O(Ŧƌ_{l})$ | $O(Ŧƌ_{l})$ |

# **SI 8. Structure of G1/S model**


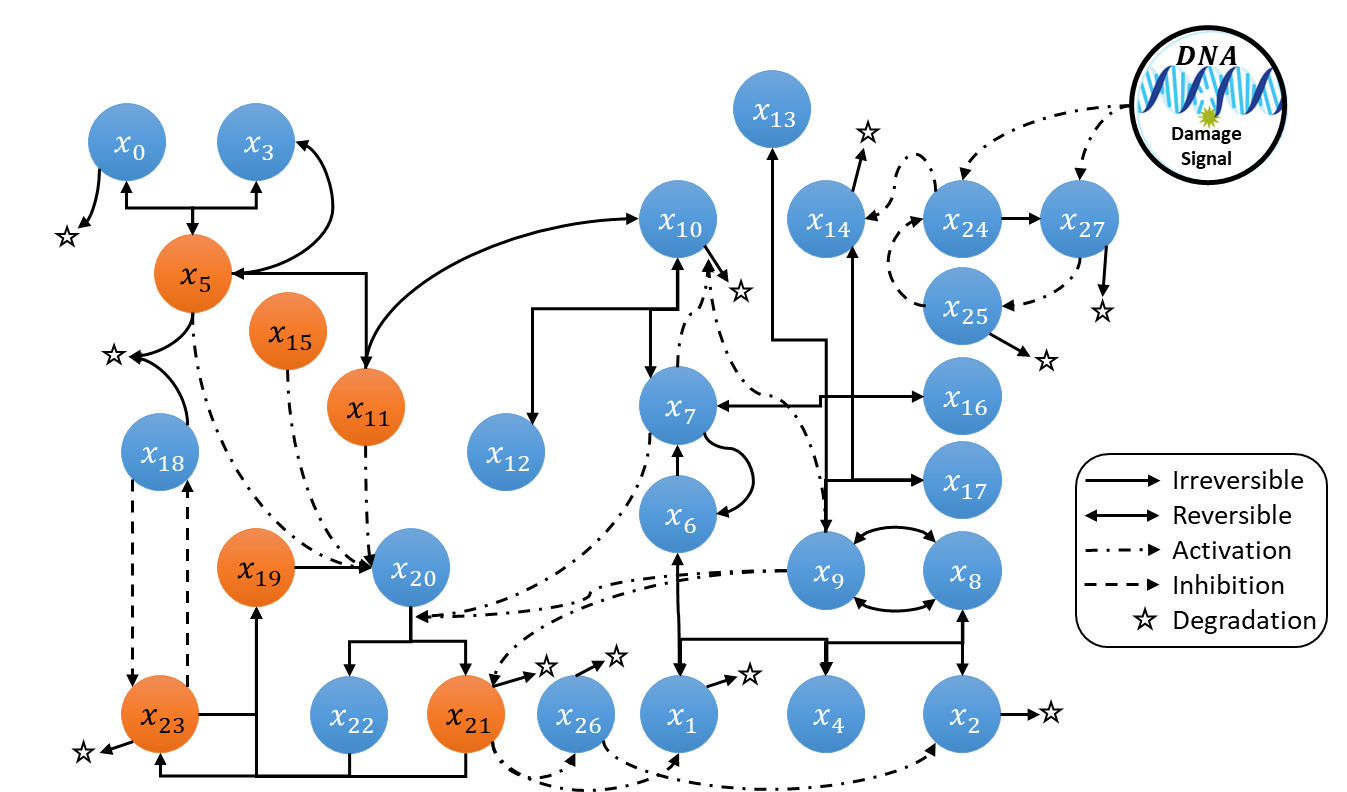


**Figure SI 16. 2D structure of the G1/S checkpoint model involving the DNA damage signal transduction pathway using model variables for** $\boldsymbol{ISP LOLAS}$**.**

# **SI 9. Conditional probabilities of the catalytic reaction system.**


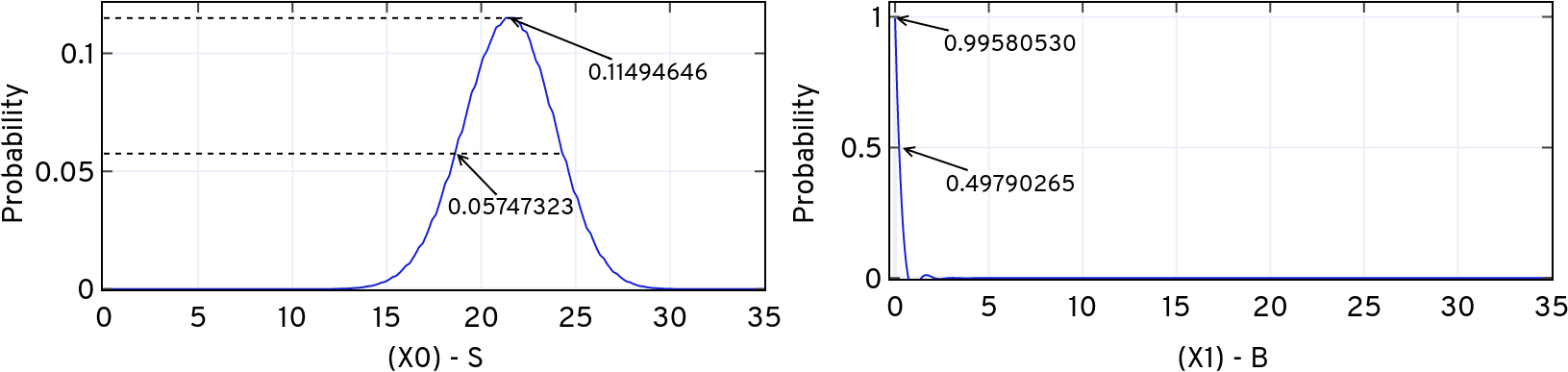


**Fig (A). Probability of** $\boldsymbol{S}$ **over** $\boldsymbol{t}_{\boldsymbol{f}}$ **Fig (B). Probability of** $\boldsymbol{B}$ **over** $\boldsymbol{t}_{\boldsymbol{f}}$


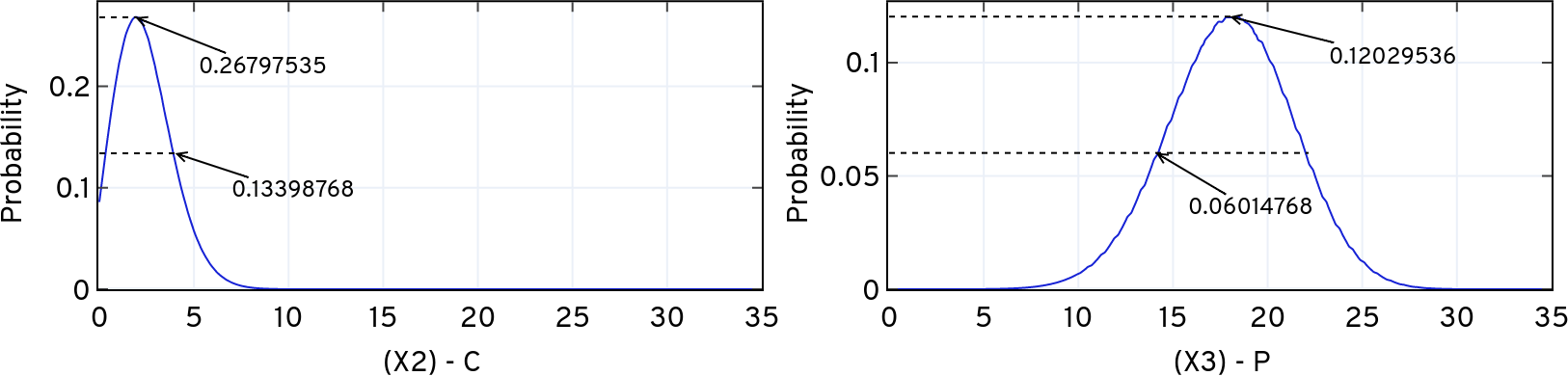


**Fig (C). Probability of** $\boldsymbol{C}$ **over** $\boldsymbol{t}_{\boldsymbol{f}}$ **Fig (D). Probability of** $\boldsymbol{P}$ **over** $\boldsymbol{t}_{\boldsymbol{f}}$


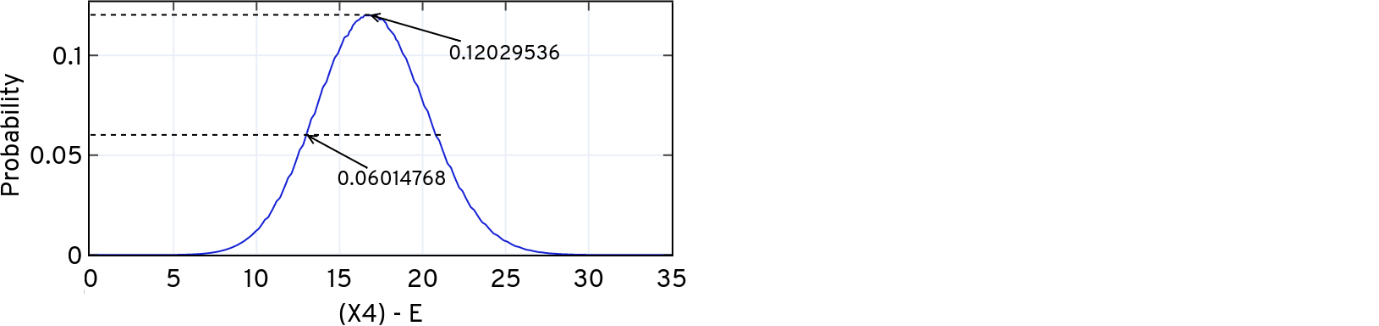


**Fig (E). Probability of** $\boldsymbol{E}$ **over** $\boldsymbol{t}_{\boldsymbol{f}}$

**Figure SI 17. Conditional probability of the catalytic system evaluated at** $\boldsymbol{t}_{\boldsymbol{f}}\boldsymbol{=0.5}$ ***sec*,** $\boldsymbol{t}_{\boldsymbol{step}}\boldsymbol{=0.01}$ **using** $\boldsymbol{LAS}$**.** Fig (A) is the probability of the species $S$ over $t_{f}$, Fig (B) is the probability of the species $B$ over $t_{f}$, Fig (C) is the probability of the species $C$ over $t_{f}$, Fig (D) is the probability of the species $P$ over $t_{f}$, Fig (E) is the probability of the species $E$ over $t_{f}$.

# **SI 10. Conditional probabilities of the dual enzymatic reaction system.**


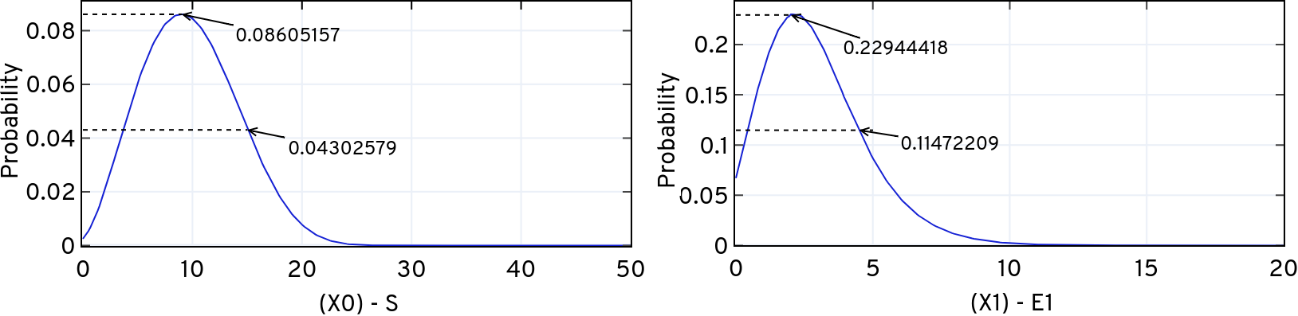


**Fig (A). Probability of** $\boldsymbol{S}$ **over** $\boldsymbol{t}_{\boldsymbol{f}}$ **Fig (B). Probability of** $\boldsymbol{E}_{\boldsymbol{1}}$ **over** $\boldsymbol{t}_{\boldsymbol{f}}$


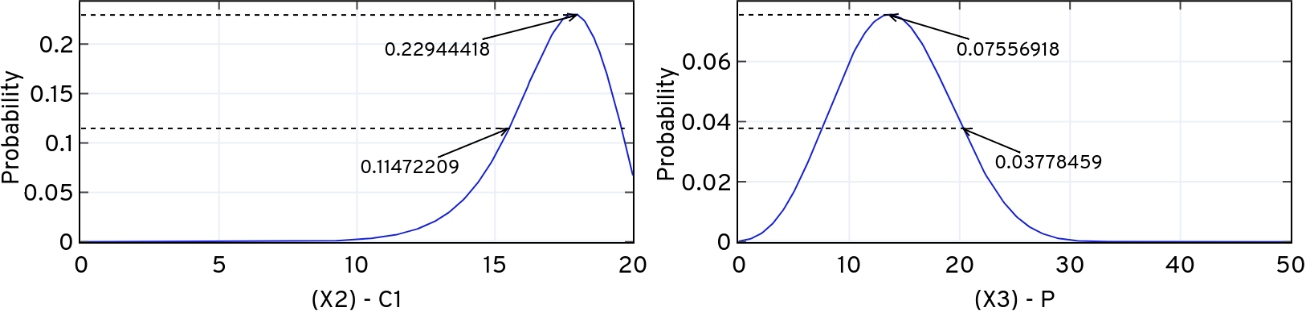


**Fig (C). Probability of** $\boldsymbol{C}_{\boldsymbol{1}}$ **over** $\boldsymbol{t}_{\boldsymbol{f}}$ **Fig (D). Probability of** $\boldsymbol{P}$ **over** $\boldsymbol{t}_{\boldsymbol{f}}$


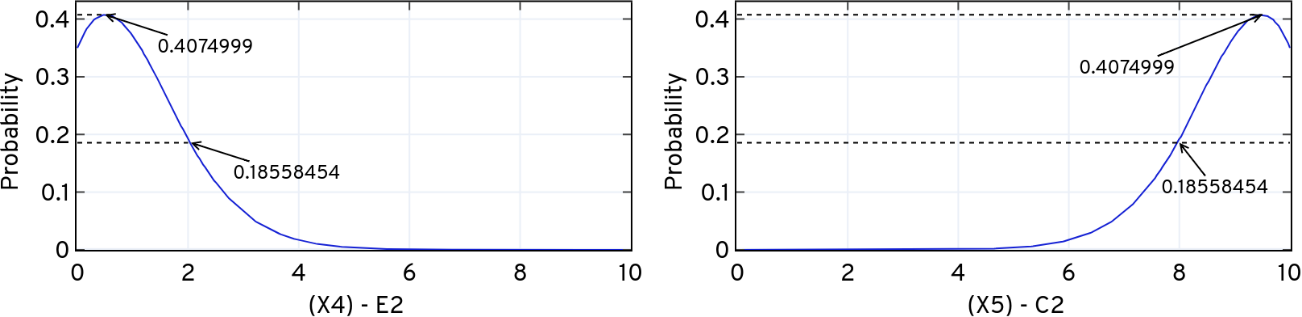


**Fig (E). Probability of** $\boldsymbol{E}_{\boldsymbol{2}}$ **over** $\boldsymbol{t}_{\boldsymbol{f}}$ **Fig (F). Probability of** $\boldsymbol{C}_{\boldsymbol{2}}$ **over** $\boldsymbol{t}_{\boldsymbol{f}}$

**Figure SI 18. Conditional probability of the dual enzymatic reactions system evaluated at** $\boldsymbol{t}_{\boldsymbol{f}}\boldsymbol{=2.0}$ ***sec*,** $\boldsymbol{t}_{\boldsymbol{step}}\boldsymbol{=0.01}$ **using** $\boldsymbol{LOLAS}$**.** Fig (A) is the probability of the species $S$ over $t_{f}$, Fig (B) is the probability of the species $B$ over $t_{f}$, Fig (C) is the probability of the species $C$ over $t_{f}$, Fig (D) is the probability of the species $P$ over $t_{f}$, Fig (E) is the probability of the species $E$ over $t_{f}$.

# **SI 11. Conditional probabilities of the G1/S model**


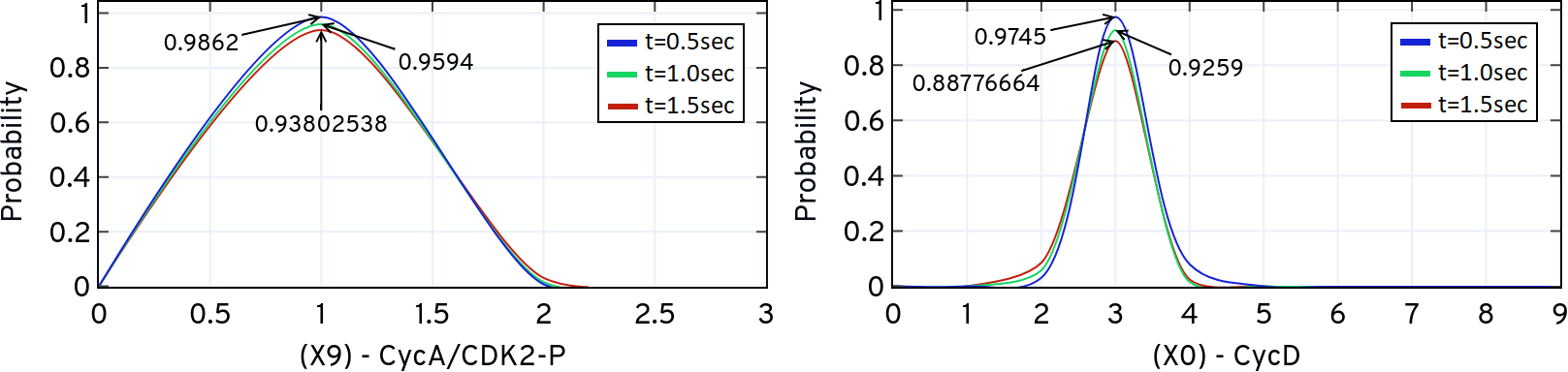


**Fig (1). Probability of** $\mathbf{CycA/CDK-P}$ **over** $\boldsymbol{t}_{\boldsymbol{f}}$ **Fig (2). Probability of** $\mathbf{CycD}$ **over** $\boldsymbol{t}_{\boldsymbol{f}}$


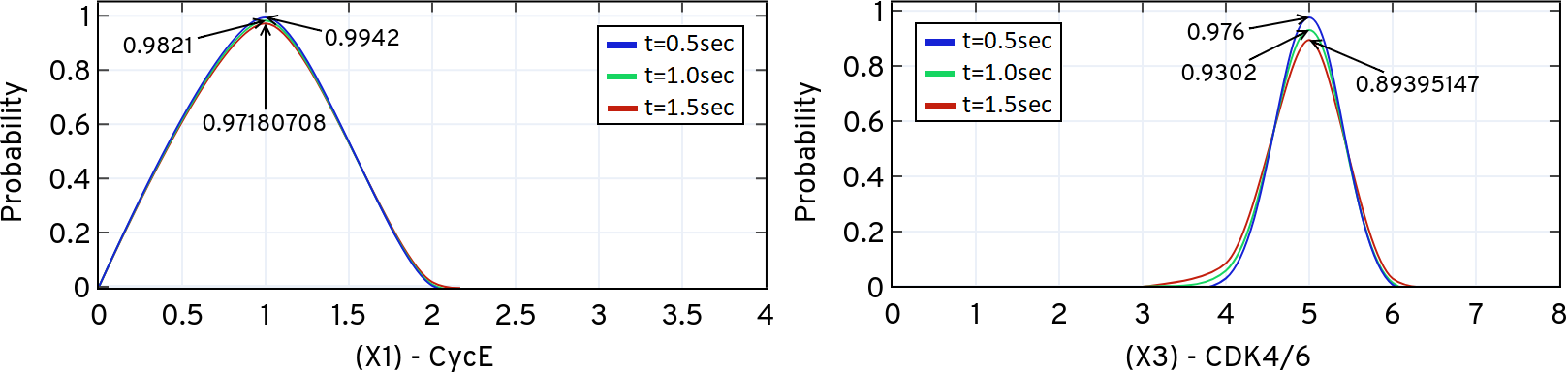


**Fig (3). Probability of** $\mathbf{CycE}$ **over** $\boldsymbol{t}_{\boldsymbol{f}}$ **Fig (4). Probability of** $\mathbf{CDK4/6}$ **over** $\boldsymbol{t}_{\boldsymbol{f}}$


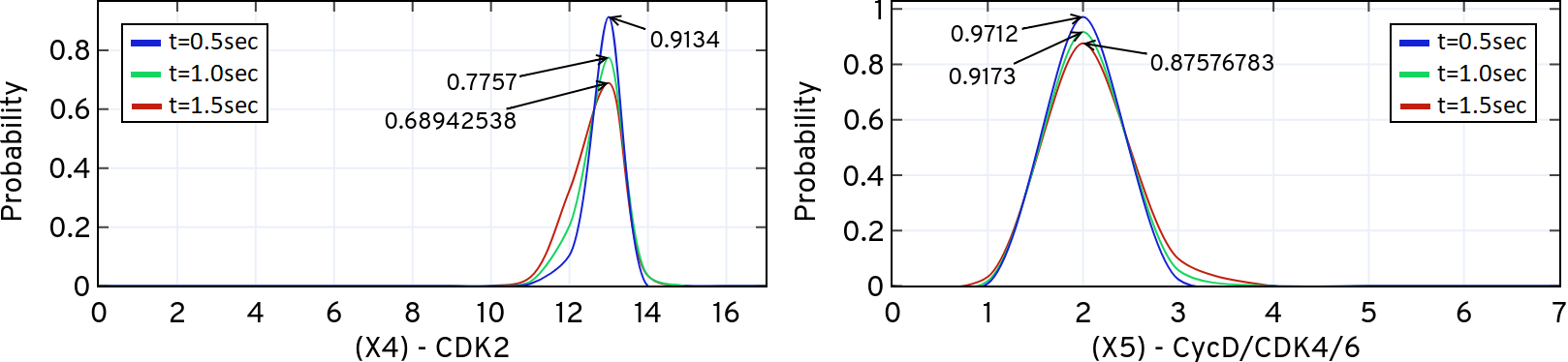


**Fig (5). Probability of** $\mathbf{CDK2}$ **over** $\boldsymbol{t}_{\boldsymbol{f}}$ **Fig (6). Probability of** $\mathbf{CycD/CDK4/6}$ **over** $\boldsymbol{t}_{\boldsymbol{f}}$


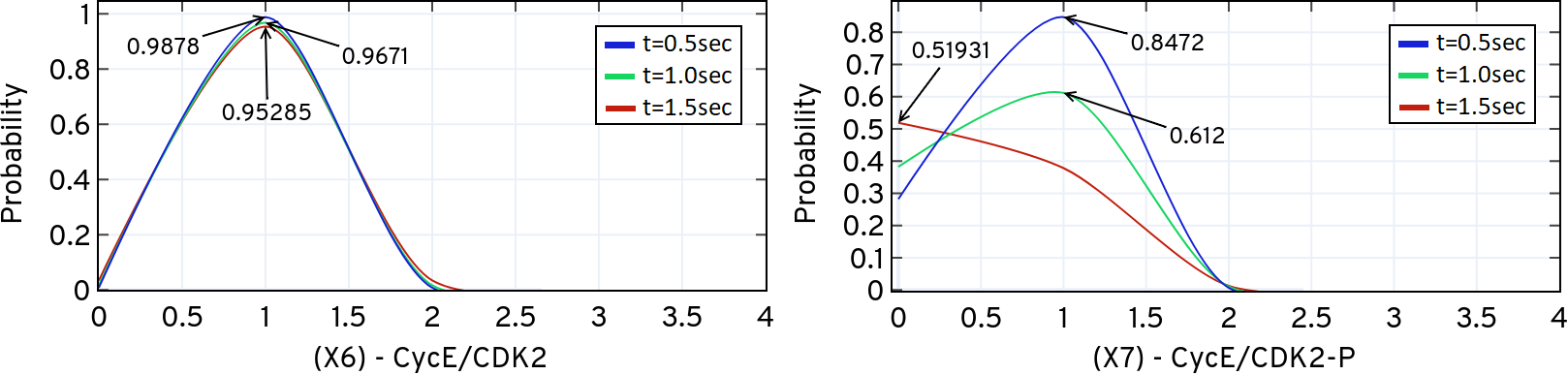


**Fig (7). Probability of** $\mathbf{CycE/CDK2}$ **over** $\boldsymbol{t}_{\boldsymbol{f}}$ **Fig (8). Probability of** $\mathbf{CycE/CDK2-P}$

**over** $\boldsymbol{t}_{\boldsymbol{f}}$


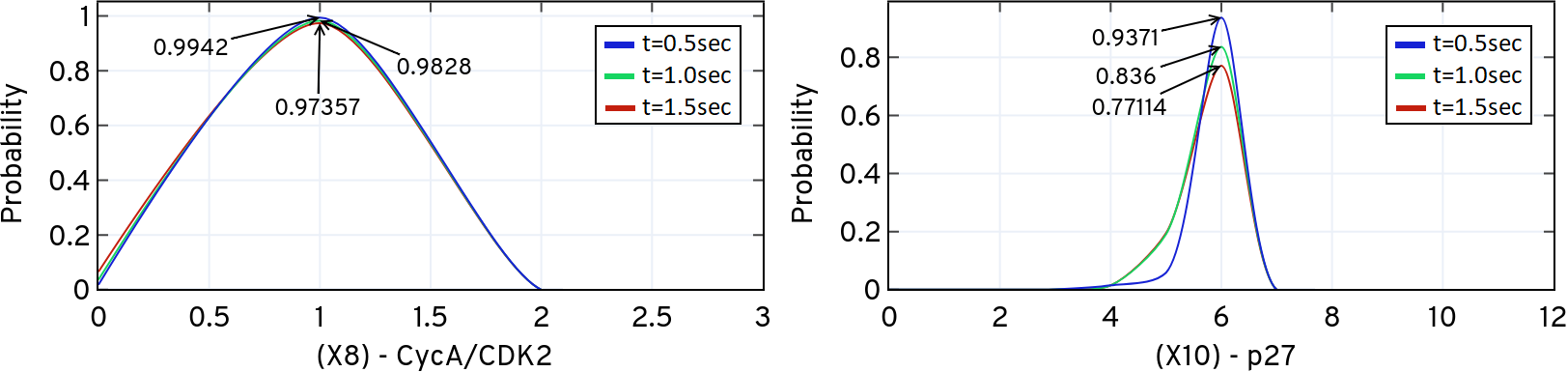


**Fig (9). Probability of** $\mathbf{CycA/CDK2}$ **over** $\boldsymbol{t}_{\boldsymbol{f}}$ **Fig (10). Probability of** $\mathbf{p}\boldsymbol{27}$ **over** $\boldsymbol{t}_{\boldsymbol{f}}$


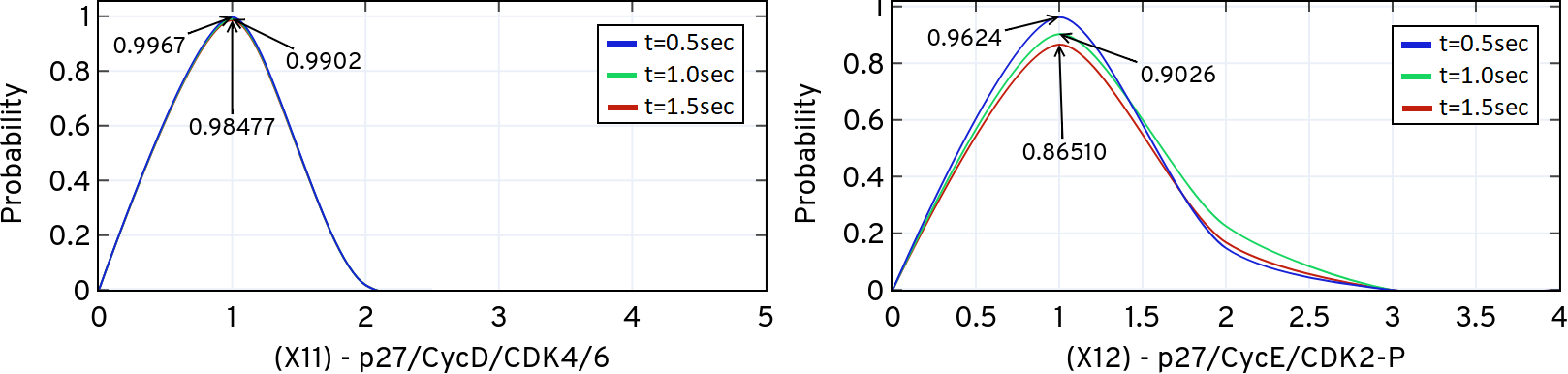


**Fig (11). Probability of** $\mathbf{p27/CycD/CDK4/6}$ **Fig (12). Probability of** $\mathbf{p27/CycE/CDK2-P}$

**over** $\boldsymbol{t}_{\boldsymbol{f}}$ **over** $\boldsymbol{t}_{\boldsymbol{f}}$


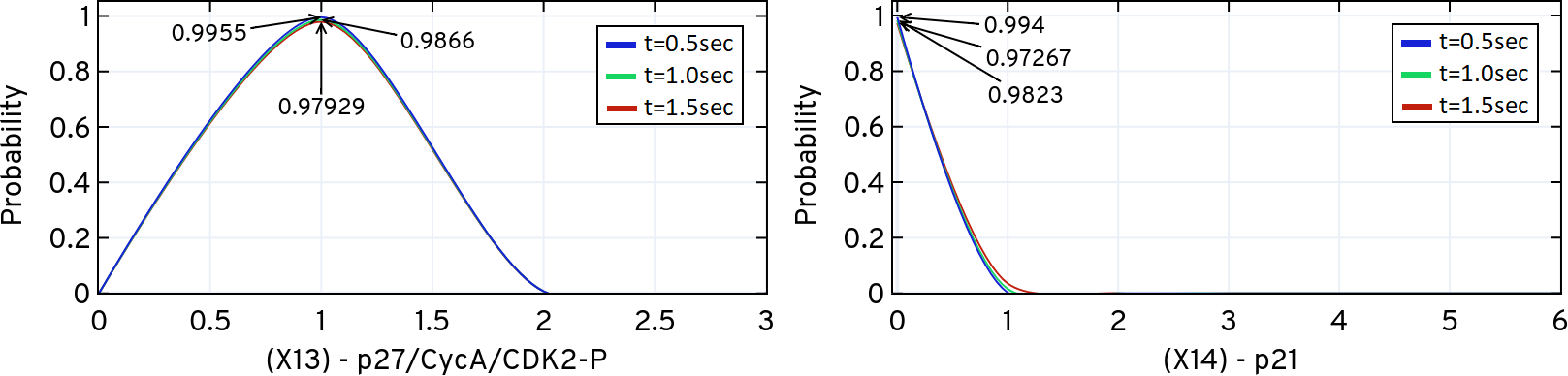


**Fig (13). Probability of** $\mathbf{p27/CycA/CDK2-P}$ **Fig (14). Probability of** $\mathbf{p21}$ **over** $\boldsymbol{t}_{\boldsymbol{f}}$

**over** $\boldsymbol{t}_{\boldsymbol{f}}$


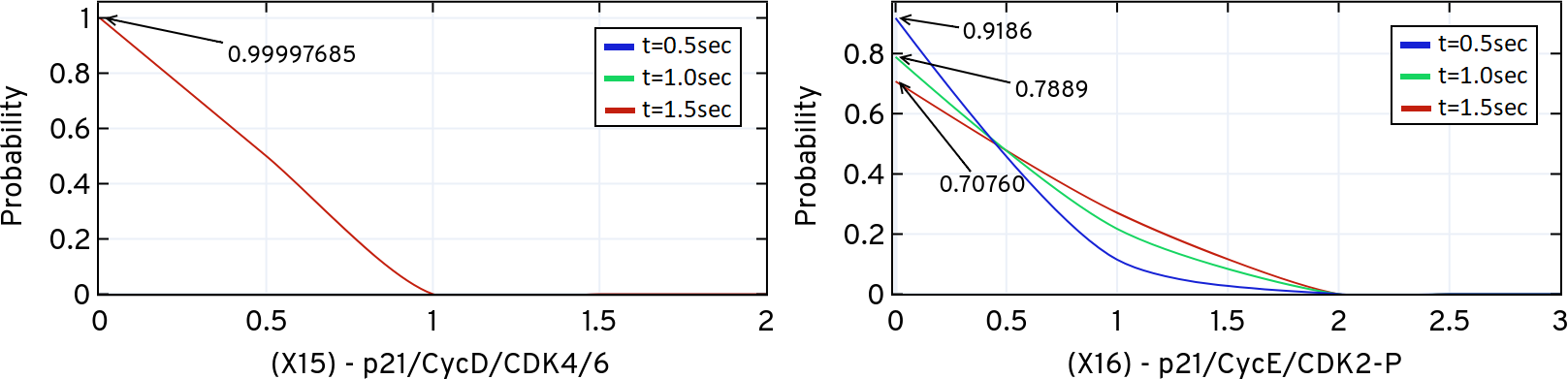


**Fig (15). Probability of** $\mathbf{p21/CycD/CDK4/6}$ **Fig (16). Probability of** $\mathbf{p21/CycE/CDK2-P}$

**over** $\boldsymbol{t}_{\boldsymbol{f}}$ **over** $\boldsymbol{t}_{\boldsymbol{f}}$


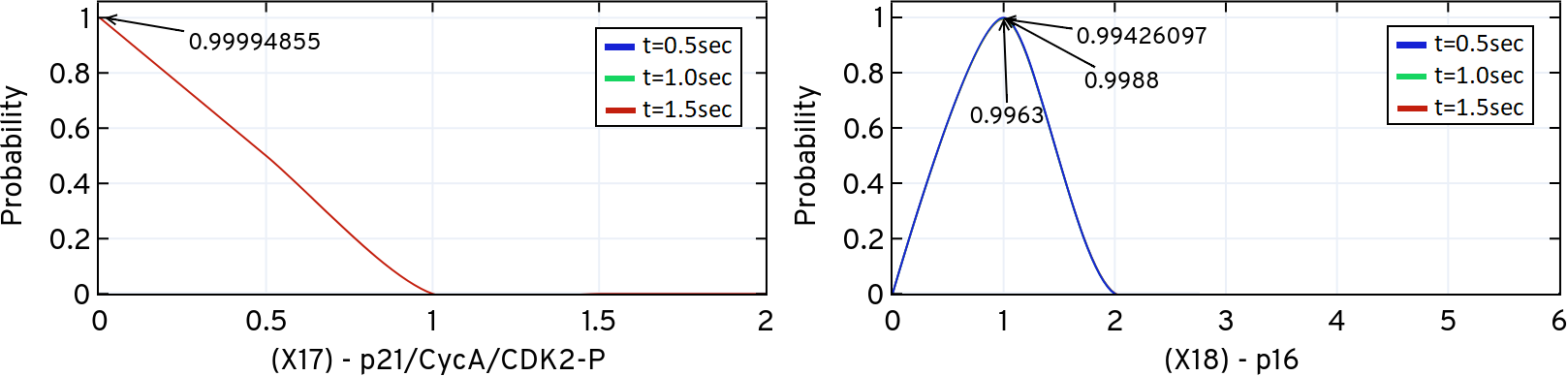


**Fig (17). Probability of** $\mathbf{p21/CycA/CDK2-P}$ **Fig (18). Probability of** $\mathbf{p16}$ **over** $\boldsymbol{t}_{\boldsymbol{f}}$

**over** $\boldsymbol{t}_{\boldsymbol{f}}$


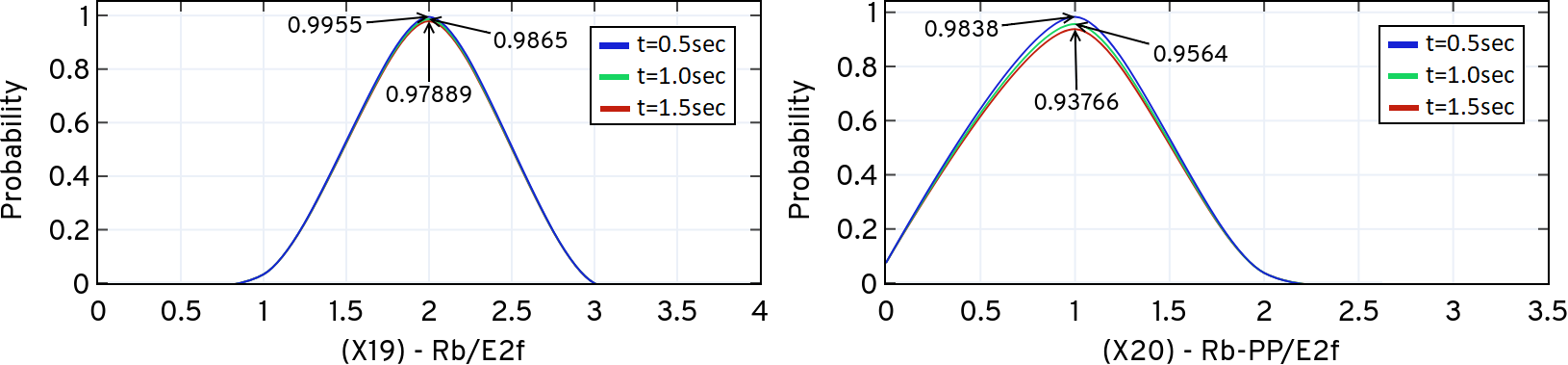


**Fig (19). Probability of** $\mathbf{Rb/E2f}$ **over** $\boldsymbol{t}_{\boldsymbol{f}}$ **Fig (20). Probability of** $\mathbf{Rb-PP/E2f}$ **over** $\boldsymbol{t}_{\boldsymbol{f}}$


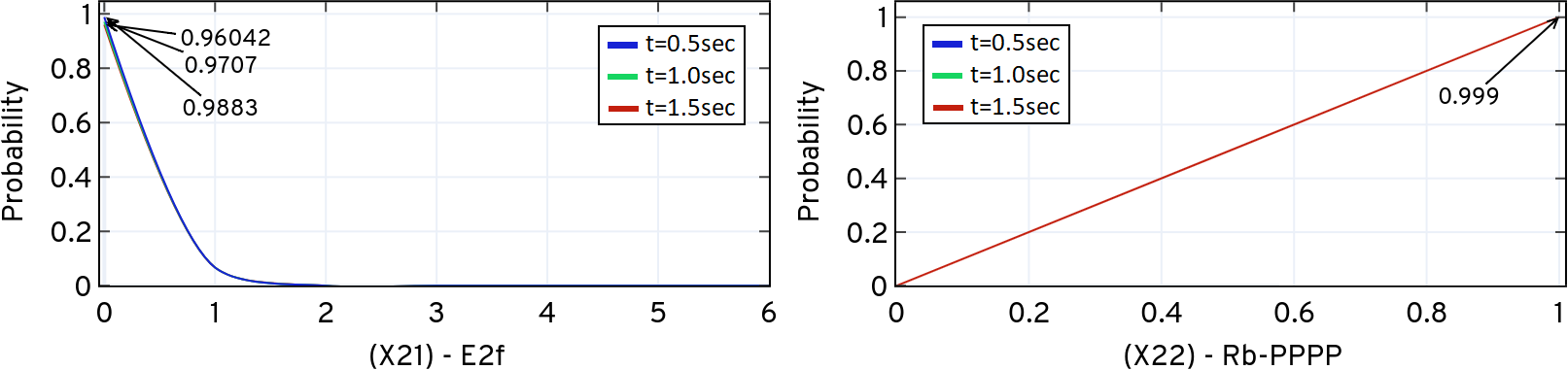


**Fig (21). Probability of** $\mathbf{E2f}$ **over** $\boldsymbol{t}_{\boldsymbol{f}}$ **Fig (22). Probability of** $\mathbf{Rb-PPPP}$ **over** $\boldsymbol{t}_{\boldsymbol{f}}$


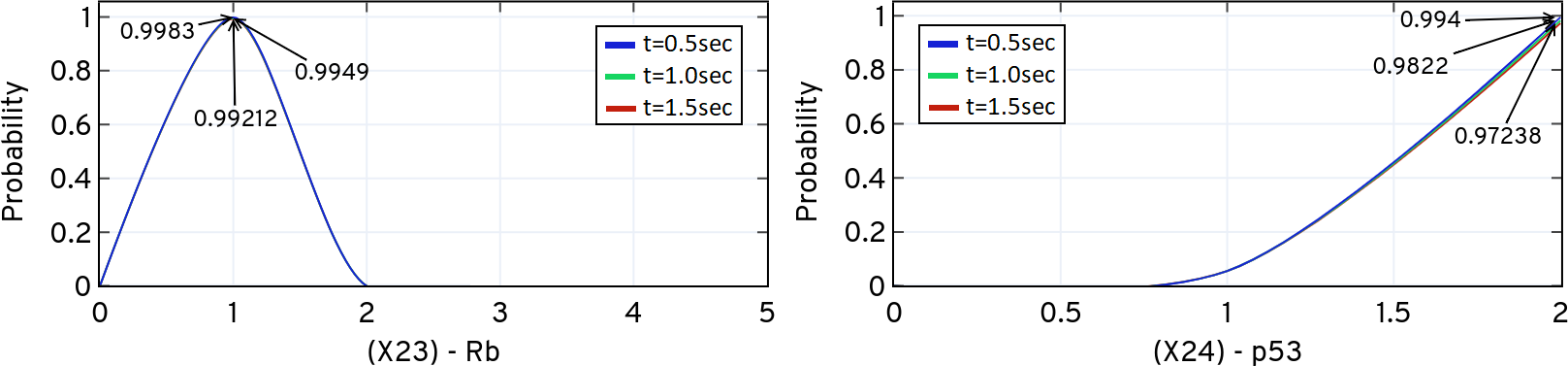


**Fig (23). Probability of** $\mathbf{Rb}$ **over** $\boldsymbol{t}_{\boldsymbol{f}}$ **Fig (24). Probability of** $\mathbf{p53}$ **over** $\boldsymbol{t}_{\boldsymbol{f}}$


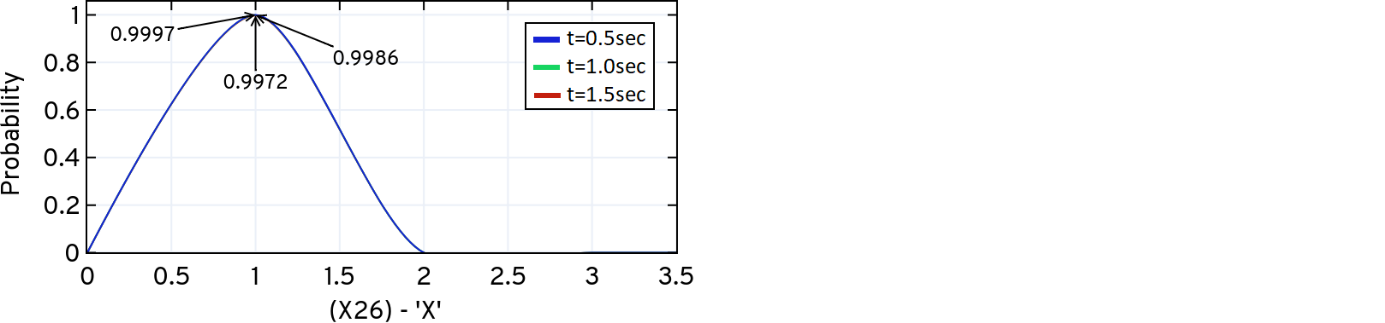


**Fig (25). Probability of** $\mathbf{'X'}$ **over** $\boldsymbol{t}_{\boldsymbol{f}}$

**Figure SI 19. Conditional probabilities of the G1/S model evaluated at** $\boldsymbol{t}_{\boldsymbol{f}}\boldsymbol{=1.5}$ ***sec*,** $\boldsymbol{t}_{\boldsymbol{step}}\boldsymbol{=0.1}$ **using** $\boldsymbol{ISP LOLAS}$**.**
